# Heterotrophic euglenid *Rhabdomonas costata* resembles its phototrophic relatives in many aspects of molecular and cell biology

**DOI:** 10.1038/s41598-021-92174-3

**Published:** 2021-06-22

**Authors:** Petr Soukal, Štěpánka Hrdá, Anna Karnkowska, Rafał Milanowski, Jana Szabová, Miluše Hradilová, Hynek Strnad, Čestmír Vlček, Ivan Čepička, Vladimír Hampl

**Affiliations:** 1 Department of Parasitology, BIOCEV, Faculty of Science, Charles University, Vestec, Czech Republic; 2 Institute of Evolutionary Biology, Faculty of Biology, Biological and Chemical Research Centre, University of Warsaw, Warsaw, Poland; 3 Institute of Molecular Genetics, Academy of Sciences of the Czech Republic, Prague, Czech Republic; 4 Department of Zoology, Faculty of Science, Charles University, Prague, Czech Republic

**Keywords:** Taxonomy, Transcriptomics, Evolution, Microbiology, Molecular biology, Comparative genomics, Whole genome amplification, Bioinformatics, Genomic analysis, DNA sequencing, Next-generation sequencing, RNA sequencing, Sequence annotation

## Abstract

Euglenids represent a group of protists with diverse modes of feeding. To date, only a partial genomic sequence of *Euglena gracilis* and transcriptomes of several phototrophic and secondarily osmotrophic species are available, while primarily heterotrophic euglenids are seriously undersampled. In this work, we begin to fill this gap by presenting genomic and transcriptomic drafts of a primary osmotroph, *Rhabdomonas costata*. The current genomic assembly length of 100 Mbp is 14× smaller than that of *E. gracilis*. Despite being too fragmented for comprehensive gene prediction it provided fragments of the mitochondrial genome and comparison of the transcriptomic and genomic data revealed features of its introns, including several candidates for nonconventional types. A set of 39,456 putative *R. costata* proteins was predicted from the transcriptome. Annotation of the mitochondrial core metabolism provides the first data on the facultatively anaerobic mitochondrion of *R. costata*, which in most respects resembles the mitochondrion of *E. gracilis* with a certain level of streamlining. *R. costata* can synthetise thiamine by enzymes of heterogenous provenances and haem by a mitochondrial-cytoplasmic C4 pathway with enzymes orthologous to those found in *E. gracilis*. The low percentage of green algae-affiliated genes supports the ancestrally osmotrophic status of this species.

## Introduction

Euglenida are a species-rich group (> 1500 described species) of unicellular eukaryotes^1^ classified into the phylum Euglenozoa and defined by both ultrastructural and molecular features. They possess one or two emergent flagella inserted in a paraflagellar pocket and reinforced by a paraflagellar rod. The surface of their cells is formed by a distinctive pellicle consisting of three layers—the cytoplasmic membrane, a proteinaceous belt supported by microtubules, and the vesicles of the endoplasmic reticulum. The pellicle enables some euglenids to move in a characteristic manner by undulated shifts in the shape of the cell, which is referred to as metaboly or euglenoid movement. The storage of carbohydrates as paramylon, a β-1,3-glucan, is also unique to euglenids ^1^.

All major types of eukaryotic nutrition are present in Euglenida—phagotrophy (eukaryovory and bacteriovory), osmotrophy, and phototrophy. Phagotrophic euglenids form several clades in the phylogenetic tree of this group, including several deepest branches^2,3^. Phototrophic euglenids (*Euglenophyceae*) which arose from one of these clades are by all means, the best-studied subgroup. Their cell contains a triple membrane-bound plastid derived from a secondary endosymbiosis of a green alga^4–6^. Since *Euglenophyceae* constitute a robust clade, it is assumed that this endosymbiosis occurred in the clade’s exclusive common ancestor^7^; however, early acquisition of a plastid in the Euglenozoa lineage has also been proposed to account for plant-like traits in trypanosomatids^8^. Although all known members of *Euglenophyceae* contain plastids, six species have lost the ability to photosynthesize and have become secondarily osmotrophic, of which the best-known is *Euglena longa* (formerly known as *Astasia longa*)^9^. Primarily osmotrophic euglenids form a distinct clade Aphagea^10^ branching within phagotrophic euglenids^2,3^. Members of the order *Rhabdomonadales* are distinguished from other members of the osmotrophic clade (i.e., *Distigma*, *Astasia*) by their lack of euglenoid movement. The genus *Rhabdomonas* contains 11 described species and is defined by one emergent flagellum, a non-flattened body, and a shallowly fluted periplast with six to nine steeply helical ridges. *R. costata* inhabits peaty waters, particularly with decaying leaves and needles.

Groups related to Euglenida are the poorly studied marine Symbiontida, Diplonemea, which have surprised scientists by their massive abundance in ocean waters^11,12^, and Kinetoplastea comprising many infamous parasites, among which species of *Trypanosoma* and *Leishmania* are some of the most studied protists^13^. Currently, mitochondrial and nuclear genomes of 72 species and strains of kinetoplastids have been sequenced^14^, but besides these there are no complete and well-annotated nuclear genome sequences available for Euglenozoa. Partial genome sequences of marine diplonemids have been obtained by single-cell approaches^11^. In Euglenida, genomic studies have as yet covered relatively well the iconic model flagellate *Euglena gracilis*, these yielding the chloroplast and mitochondrial genomes^15,16^ and a very fragmented and unannotated nuclear genome^17^. Genomic datasets from the remaining euglenids comprise exclusively the chloroplast genomes, 30 of which have been published until present^15,18–20^. Recently, the proteomes of *E. gracilis* plastid^21^ and mitochondrion^22^ have been characterised by mass spectrometry proteomics. Similar studies on the heterotrophic euglenids are completely lacking, which is a great pity because they would bring insights into the basic cellular processed of the ancestral forms in the group before the plastid endosymbiosis. Our genomic and transcriptomic investigation of primary osmotroph *Rhabdomonas costata* aims to start filling this gap.

## Results

### Microscopy and phylogenetic position

**Figure 1:**
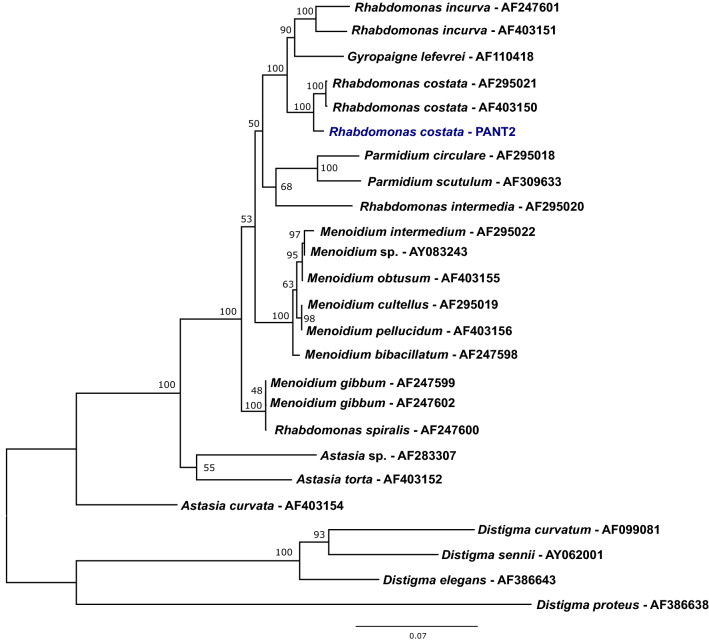
Phylogenetic tree of euglenids based on the 18S rRNA gene. The tree was constructed in IQ-TREE using the TIM2e + G4 model selected by Bayesian information criterion from a trimmed alignment containing 1569 nucleotide positions. The values at the nodes represent ultrafast bootstraps from 1000 repetitions, where above 50. The strain analysed in this study is shown in blue. The tree was rooted by the genus *Distigma*. The figure was created in FigTree v 1.4.4.

**Figure 2:**
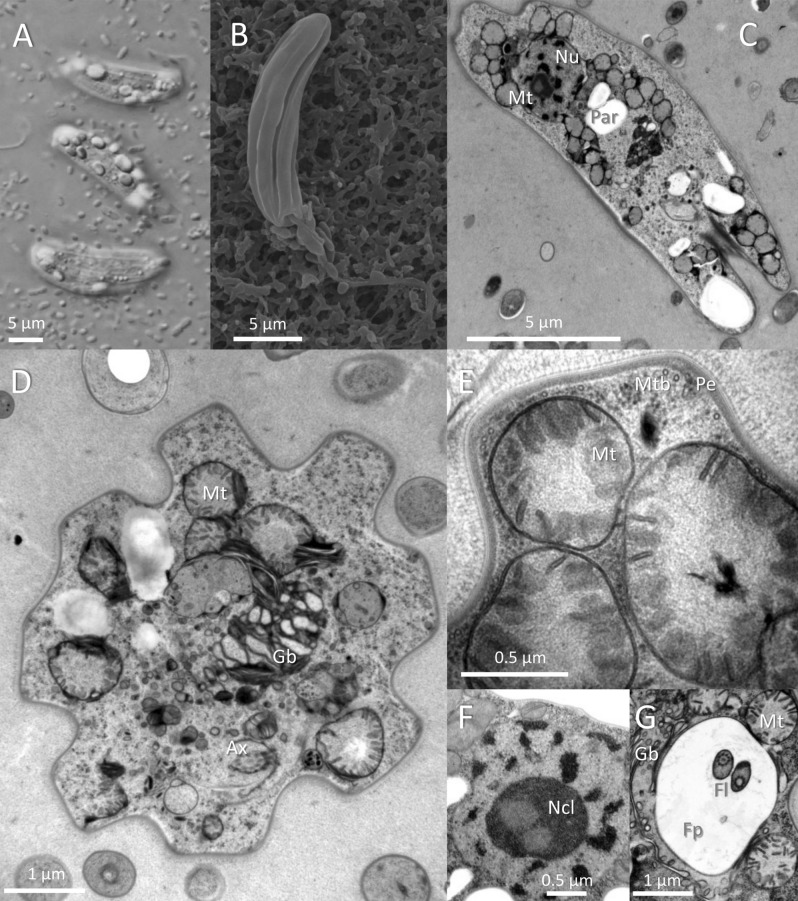
Microscopic investigation of *Rhabdomonas costata.* Cells in DIC contrast (**A**) with visible pellicular stripes and paramylon grains. SEM microscopy (**B**) of a cell showing surface invaginations and a flagellum inserted in the flagellar pocket. Longitudinal (**C**) and transverse (**D**) TEM sections and details of the pellicle and mitochondria (**E**), nucleus (**F**), and flagellar pocket with two flagella (**G**). Ax—axoneme, Gb—Golgi body, Fl—flagella, Fp—flagellar pocket, Mt—mitochondrial cross-sections, Mtb—subpellicular microtubules, Nu—nucleus, Ncl—nucleolus, Par—holes after paramylon grain, Pe—pellicle.

We established monoeukaryotic culture (isolate PANT2) of a heterotrophic euglenid from a freshwater pond. Based on the phylogenetic analysis of the gene for 18S rRNA (Fig. 1) and microscopic observations (Fig. 2), we identified the organism as *Rhabdomonas costata* (Korshikov) Pringsheim 1942. The cells were observed by light and electron microscopy (Fig. 2).

They possess two flagella inserted in the flagellar pocket, but only one extends beyond. The surface of the cell is formed into 7–9 ridges supported by the pellicle and microtubules. Conspicuous paramylon grains were observed in the cytoplasm, as were many mitochondrial cross-sections with discoidal cristae. The nucleus contains a large nucleolus and multiple heterochromatin regions (Fig. 2).

### General characteristics of the genome and transcriptome

We generated a genomic draft of *R. costata*, the basic metrics of which are summarised in Table 1. The assembly is very fragmented as it consists of 36,105 contigs above 1 kb, N50 being 1194 and the estimated coverage 10–20× based on the k-mer frequency and mapping of the reads to the genome. The total length of the assembly is 106.9 Mbp but the estimated genome size based on k-mer frequency reached 128 Mbp. The latter estimate must be treated with great caution given the fact that the reads were generated from the amplified sample. Another indication that the genome is quite incomplete is that only 43.5% of transcriptome reads mapped to the assembly. We did not proceed to gene prediction due to insufficient quality of the genomic draft.

**Table 1:** Parameters of the genomic and transcriptomic assemblies.

	Genome assembly	Transcriptome assembly
Number of reads Miseq	11,624,864	12,103,119
Number of reads Hiseq	82,258,718	0
Number of contigs	143,763	93,852
Number of contigs (≥ 500 bp)	82,982	NA
Number of contigs (≥ 1000 bp)	36,105	19,335
Number of contigs (≥ 5000 bp)	153	NA
Median contig length (bp)	661	430
Median contig length (≥ 500 bp)	924	NA
Max contig length (bp)	8093	17,913
Total length (bp)	106,888,161	66,880,466
Total length (≥ 500 bp)	94,209,074	NA
Total length (≥ 1000 bp)	58,674,546	NA
N50 (bp)	1194	NA
L50	25,493	NA
Fraction of GC (%)	51.63	58.25
Number of N's per 100 kbp	31.22	NA
Predicted putative proteins	NA	55,783
Unique proteins after decontamination	NA	39,456
Any homologue in NCBI nr (e-value ≤ 10^−5^)	NA	25,933

**Figure 3:**
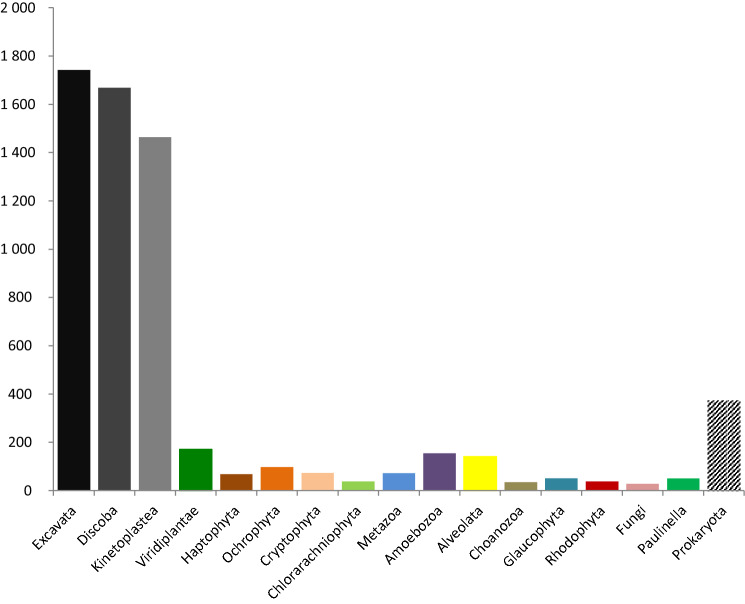
Graph summarising taxonomical affinities of the predicted proteins of *Rhabdomonas costata*. 3,129 protein phylogenies, in which *R. costata* was robustly (BS ≥ 75) placed into a taxonomically homogeneous clan, were sorted accordingly into taxonomic bins. Discoba and Kinetoplastea represent the subgroups of Excavata.

We sequenced a transcriptome and obtained 93,852 transcripts, from which we predicted 39,456 unique proteins after filtering prokaryotic contaminations and redundancy (Table 1). Completeness of the transcriptome measured by BUSCO (complete BUSCOs: 75.0%, fragmented BUSCOs: 12.5%, missing BUSCOs: 12.5%, n: 303) was satisfactory and comparable to published transcriptomes of euglenophytes (missing BUSCOs in *Euglena gracilis*, *E. longa*, and *Eutreptiella gymnastica* were 8.3%, 7.3%, and 39.3%, respectively), suggesting that this data set is sufficient for describing the selected features of *R. costata*. We annotated proteins automatically by BLAST against NCBI nr and KEGG^23^; only 25,933 proteins recovered a homologue (e-value < 10^−5^) in NCBI and 9,430 proteins received KEGG annotations (Supplementary Fig. S1 online). The taxonomic affiliations of *R. costata* proteins are summarized in Fig. 3. As expected, most proteins of the 3129 for which a relationship could be robustly established (bootstrap 75 or higher) affiliated with taxa belonging to Discoba (53%), 12% of the proteins branched with prokaryotes and 32% affiliated with any of the other small bins. The proteins that affiliated with prokaryotes may represent contamination, but some may represent *R. costata* proteins having strong affiliation to prokaryotic homologues due to increased divergence, absence of eukaryotic homologues in the nr database, or origin via horizontal gene transfer.

We have searched all 93,852 transcripts for the presence of the published sequence of the *R. costata* spliced leader sequence (ACATTACTGGGTGTAATCATTTTTTCG)^24^. Only 15.3% of transcripts contained the 10-nucleotides partial SL (CATTTTTTCG) or a longer fraction of the full-length SL at one of the ends. The longest part of the SL we were able to find was only 17 nucleotides long (out of the 27 published). More details and comparison with other euglenid transcriptomes are given in Supplementary Table S1.

### Intron characteristics

Although it was not suitable for gene prediction, we used the genomic assembly of *R. costata* for an analysis of the types of introns. Introns were detected by mapping the transcripts to genomic contigs. First, this mapping was done manually in genes for which the presence of introns has been reported in other euglenids^25^. We have identified 29 complete introns in these six genes (Table 2). Some gene regions were not covered by transcripts, and so the presence of additional introns in these genes cannot be excluded. All completely detected introns have conventional GT/AG boundaries. For 14 introns, only one end was found in the data, and these were marked as incomplete. The type of these introns could not be determined with certainty, but all of them have at least one end with conventional boundary.

**Table 2:** Introns identified in selected genes of *Rhabdomonas costata.*

Coded protein	Gene abb	Complete conventional introns	Incomplete introns *	ORF length (nt)	gDNA coverage (%)**
α-tubulin	*tubA*	2	0	1356	100
β-tubulin	*tubB*	4	0	1335	100
γ-tubulin	*tubG*	6	7	1680	76
Heat shock protein 90	*hsp90*	10	5	2112	68
GAPDH	*gapC*	3	1	1062	70
Fibrillarin	*nop1p*	4	1	903	85

*Only one intron boundary was found in the data, thus the intron type could not be determined with certainty.

**Part of ORF length mapped to gDNA. The number of introns may not be definitive in low-percentage coverage.

The positions of the introns in the two best-studied genes in this respect (*tubA* and *tubB*) were compared with their homologues in other euglenids (Supplementary Fig. S2 online). All *R. costata* introns were in the same positions as those described by Milanowski et al.^25^ in tubulin genes of the primary heterotroph *Menoidium bibacillatum*. The second conventional intron in *tubA* of the two heterotrophs is in a position identical to the intron in phototrophic euglenids. The positions of all other introns in the tubulin genes of heterotrophs and phototrophs are different. Only one of 15 introns in *hsp90* is in the same position as in *Euglena agilis hsp90*; on the other hand, the gene for *R. costata* fibrillarin shares 3 of 4 intron sites with the gene of *E. gracilis* (not shown)*.*

**Figure 4:**
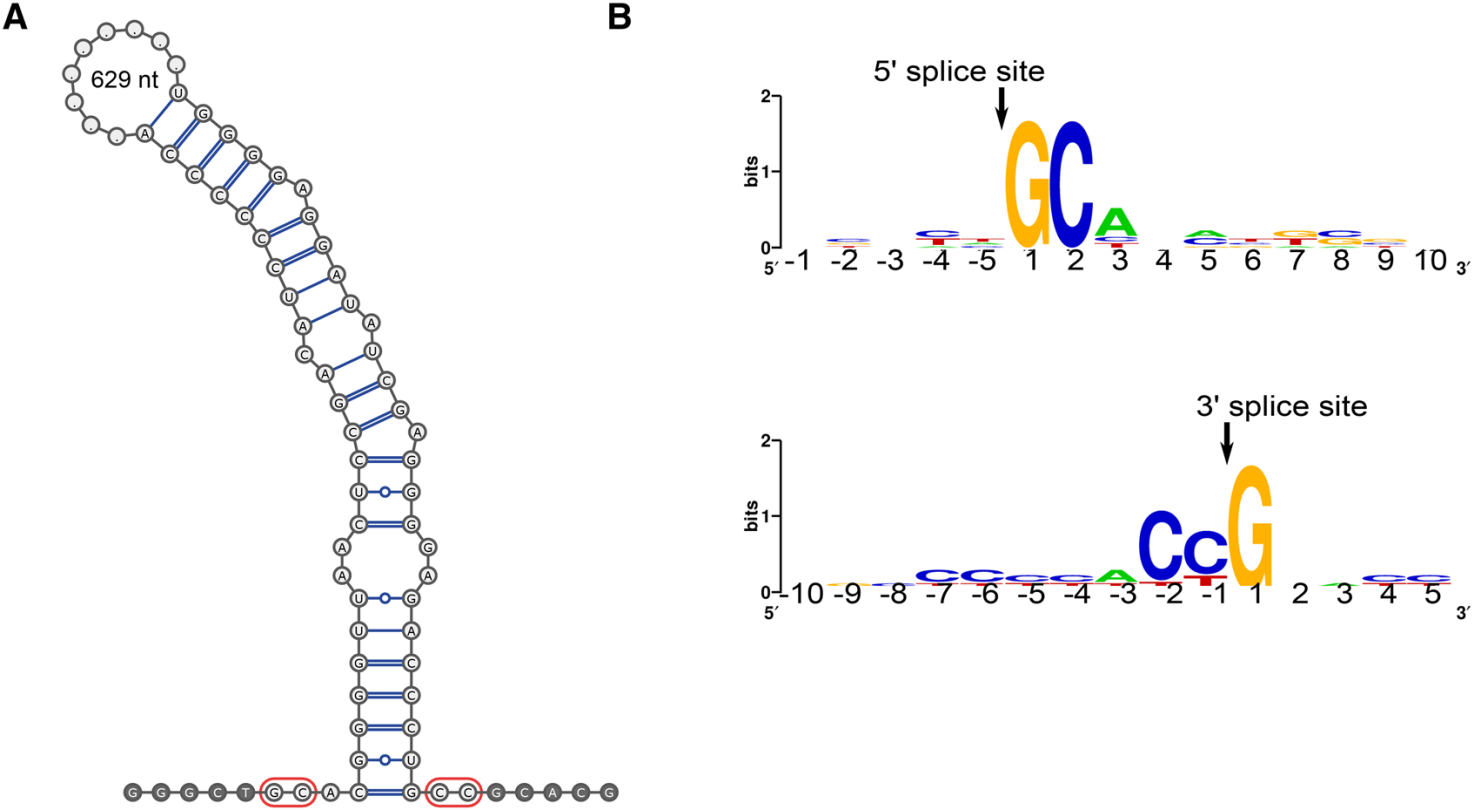
Example of the nonconventional intron of *R. costata*. The secondary structure of a nonconventional intron from NODE_718 (**A**), and the sequence logo of the boundaries of seven putative nonconventional introns detected in *R. costata* (**B**).

We also predicted the putative introns by mapping the transcriptome to the genome assembly (Supplementary Tables S2 and S3 online), revealing 105 contigs containing putative introns with nonconventional boundaries (not GT(GC)/AG) (Supplementary Table S4 online). Of these, 26 represented genes with homologues in NCBI detected by blastx (Supplementary Table S5 online). These were manually inspected. The manual inspection revealed seven cases of putative nonconventional introns with boundaries confirmed by a transcript and reads mapping (Supplementary Table S6 online), of which four are very likely nonconventional introns as no alternative transcripts were observed. One example intron in the hypothetical protein encoded in the genomic contig NODE_718 and the transcript TR27401 is shown in Fig. 4.

### Mitochondrial genome contigs

Contigs containing sequences homologous to all seven protein-coding genes reported from the *E. gracilis* mitochondrial genome were detected in the genome assembly (Supplementary Table S7 online). In the cases of four genes, the contigs were manually curated and by mapping of reads and merging they were prolonged to cover the complete genes for cytochrome c oxidase subunit 1 (cox1) (NODE_56929 + NODE_75143), cytochrome c oxidase subunit 2 (cox2) (NODE_56949), cytochrome b (cob) (NODE_51506 + NODE_82781), and partial NADH dehydrogenase subunit 4 (nad4) (NODE_12379 + NODE_77849). Translation of ORFs using the genetic code of *E. gracilis* mitochondrion (translation code 4, UGA encoding Trp) left many UAG stop codons in positions, which were occupied by tyrosine in homologues of most other euglenophytes and euglenozoans (Supplementary Fig. S3 online). There is no indication of RNA editing and introns across these genes. All these observations suggest that these four contigs represent parts of mt genome, which uses a variant of genetic code different from *E. gracilis* mitochondrion. We were unable to complement any other putative genes of *R. costata* mitochondrion.

### Mitochondrial proteome

We used the set of proteins predicted from the transcriptome to in silico determine the mitochondrial proteome. The mitochondria of *R. costata* strain PANT2 exhibit well-developed cristae (Fig. 2e), yet the organism is able to survive short-term (one month) cultivation in complete anaerobiosis. From the 1538 proteins of the predicted mitochondrial proteome, 1,107 were assigned to functional categories adopted from KEGG (Supplementary Table S8 online, Supplementary Fig. S4 online). 1274 proteins have orthologues in the experimentally established and manually curated proteome of the *E. gracilis* mitochondrion^22^. In accordance with the presence of mitochondrial genome, the mitoproteome set contains a repertoire of over 140 proteins involved in DNA and RNA metabolism, ribosome biogenesis, and translation (Supplementary Table S8 online). These include two of the already published DNA polymerases I^26^.

Twenty aminoacyl-tRNA synthetases, i.e. enzymes charging the tRNA with corresponding amino acid, fell among the putative mitochondrial proteins. We have decided to investigate this group of enzymes in more details in order to establish their relationships to homologues in other euglenids and their evolution within the group. We used the advantage of the fact that the localisation of these enzymes has been predicted in silico for *Euglena longa*^27^ and established using proteomics in *E. gracilis*^21,22^. We identified 30 *R. costata* transcripts covering all 20 proteinogenic amino acids and reconstructed their phylogenies (Supplementary Table S9, Supplementary Figs. S5–S23 online). Using relationships to annotated *Euglena* spp. homologues and the mitochondrial targeting predictions as hints, we tried to establish whether the enzymes function in the mitochondria or cytosol of *R. costata* (summarised in Supplementary Table S9 online). While we were not able to confirm the presence of mitochondrial aminoacyl-tRNA synthetases for only three amino acids (tyr, arg, trp), the set clearly lacks cytosolic aminoacyl-tRNA synthetases for at least nine amino acids (ala, gly, his, ile, leu, lys, pro, ser, val). Such bias can hardly be explained by the incompleteness of the data and it rather suggests that these enzymes are dually localised and charge tRNAs in both compartments as was already shown for many organism including *Trypanosoma brucei*, where this holds for an almost complete set^28^. The evolutionary history of some of the enzymes was noteworthy. In the cases of cysteinyl-tRNA synthetase and phenylalanyl-tRNA synthetases (Supplementary Figs. S9 and S17), *R. costata* homologues appear sister to the clade of both mitochondrial and chloroplast orthologues in euglenophytes suggesting that the gene for mitochondrial enzyme has been duplicated after the plastid was acquired and one copy was repurposed for translation into the plastid. The situation in threonyl-tRNA synthetases (Supplementary Fig. S20) was similar, but the phylogeny suggests gene duplication already in the common ancestor of *R. costata* and euglenophytes, i.e. before the plastid acquisition. Finally, both tyrosyl-tRNA synthetases identified in *R. costata* are clearly unrelated to enzymes of euglenophytes. This may be related to the probable codon shift observed in *R. costata* mitochondrial genome. Unfortunately, none of these proteins has been predicted to localise into the mitochondrion, but it should be noted that one of them is heavily truncated at the N-terminus making the phylogenetic inference difficult and targeting the prediction impossible.

**Figure 5:**
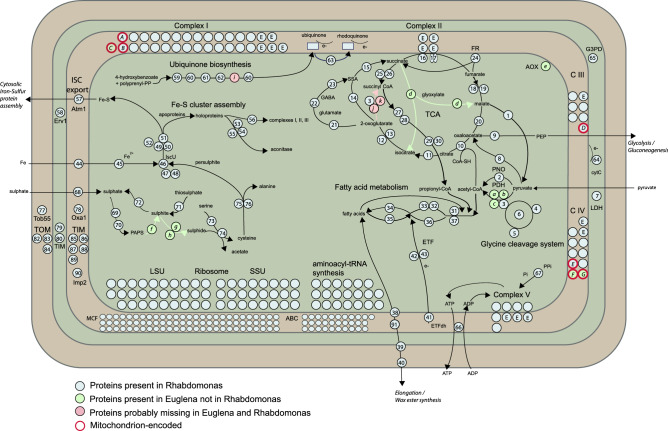
Metabolic map of the *R. costata* mitochondrion. The map is based on *in silico* prediction of the mitochondrial proteome from the transcriptomic dataset. Blue circles represent enzymes present in *R. costata* with homologues in *E. gracilis*, light green circles represent enzymes absent in *R. costata* but present in *E. gracilis,* and pink circles represent typical eukaryotic enzymes missing in both euglenids. The figure allows for comparison, inspired by Ebenezer et al. 2019. Abr.: E in complexes—Euglenozoa specific subunits, Lowercase letters—enzyme absent in *R. costata* mitochondrion: a/ pyruvate dehydrogenase E1 component alpha subunit, b/ pyruvate dehydrogenase E1 component beta subunit, c/pyruvate dehydrogenase E2 component, d/ bifunctional glyoxylate cycle enzyme malate synthase/isocitrate lyase, e/ alternative oxidase, f/ phosphoadenosine phosphosulphate reductase, g/ sulphite reductase (NADPH) flavoprotein alpha-component, h/ sulphite reductase (NADPH) hemoprotein beta-component, i / 3-demethoxyubiquinol 3-hydroxylase, j/ 2-oxoglutarate dehydrogenase E1 component, k/ 2-oxoglutarate dehydrogenase E2 component. Capital letters—mitochondrial encoded enzymes: A/ cytochrome c oxidase subunit 1, B/ cytochrome c oxidase subunit 2, C/ cytochrome c oxidase subunit 3, D/ cytochrome b, E/ NADH dehydrogenase subunit 4, F/ NADH dehydrogenase subunit 1, G/ NADH dehydrogenase subunit 5. The numbers correspond to the designations in Supplementary Table S8 online and the main text.

Predicted functions and metabolic pathways of the mitochondrion are summarised in Fig. 5 and for reader’s convenience the protein numbers and letters from the Fig. 5 are referred in the following text. Pyruvate and malate are probable substrates for energy metabolism. A malic enzyme (nr. 1) catalyses the oxidative decarboxylation of malate to pyruvate. Pyruvate: NADH oxidoreductase (PNO, nr. 2) is the only enzyme in the transcriptome with activity for the oxidative decarboxylation of pyruvate to acetyl-CoA. It is present in five transcripts that are not identical in sequence, representing at least three different versions of the protein. The canonical mitochondrial pyruvate dehydrogenase complex (PDH, letters a-c) is apparently absent, as only the E3 subunit (dihydrolipoamide dehydrogenase; nr. 3) was recovered in the dataset. Transcript RCo000646 for subunit E1 is likely a contamination, as it has a 95% identity with the bacterium *Magnetospirillum aberrantis* according to the blastp. The subunit E3 is also a component of other mitochondrial enzyme complexes, and in *R. costata* it is probably involved in the glycine cleavage system. Pyruvate can alternatively be shuttled into the TCA cycle via oxaloacetate by pyruvate carboxylase (nr. 8) or reduced to L-/D-lactate by lactate dehydrogenases (nr. 7). The presence of phosphoenolpyruvate carboxykinase (nr. 9) allows production of phosphoenolpyruvate, a substrate for gluconeogenesis. There are also three similar copies of 1,3-β-D-glucan synthase (RCo003309, RCo003310, RCo022891). This enzyme is orthologous to the *E. gracilis* glucan synthase-like 2 protein, which is responsible for the synthesis of paramylon^29^ and has been reported in the mitochondrial proteome of *E. gracilis*^22^. Unlike *E. gracilis* proteins, we consider this protein cytosolic, because the probability of its mitochondrial localisation based on prediction software was low. This is consistent with the cytosolic localization of paramylon grains in the cytoplasm of *R. costata* (Fig. 2). The enzyme endo-1,3(4)-β-glucanase, which is potentially involved in the degradation of paramylon, is also present (RCo017523, RCo019147, RCo047076, RCo017521, RCo018319, and RCo023166) and *in silico* predicted to localise in the cytosol.

The TCA cycle seems complete. The 2-oxoglutarate dehydrogenase complex (letters j-k) is absent, but this step is bypassed by enzymes 2-oxoglutarate decarboxylase (nr. 14) and succinate-semialdehyde dehydrogenase (nr. 15). Both subunits of succinyl-CoA synthetase are present (nrs. 25 and 26) and this enzyme probably works in the direction of succinyl-CoA formation, thus leading to fatty acid synthesis. The GABA shunt (nrs. 21–23) is present, but the glyoxylate cycle enzymes (letter d) are absent. Succinate dehydrogenase (SDH, Complex II) is similar in subunit composition to kinetoplastid SDH^30^. In total, six subunits were found, including the conserved eukaryotic subunits SDH1-2 (SDHA-B) and Euglenozoa-specific subunits 6–9^30^. The FeS cluster-containing subunit SDH2 (nr. 17) splits into two polypeptides (N- and C- terminus), similar to that in trypanosomes and *E. gracilis*^31^. The Euglenozoa-specific subunits SDH5, 10 and 11 are missing from the data, as well as from the *E. gracilis* transcriptome^17^. Other components of the respiratory chain detected in the transcriptome include Complex I (29 subunits including 5 Euglenozoa-specific), Complex III (6 subunits), complex IV (8 subunits), complex V - F_o_F_1_ ATPase (subunits of F_1_ part α, β, γ, δ and OSCP and three Euglenozoa-specific, subunits of F_0_ other 8 subunits), and electron-transferring-flavoprotein dehydrogenase (nr. 41). The alternative oxidase (AOX, letter e) that was reported in other Euglenozoa, is absent from the transcriptome and was not detected by PCR with specific primers. Three types of membrane-associated electron carriers are present—cytochrome c, ubiquinone (UQ; most enzymes involved in its synthesis are present in the transcriptome, nrs. 59–62), and rhodoquinone (RQ), which is formed from ubiquinone by rhodoquinone methyltransferase (nr. 63). Soluble electron-transferring flavoprotein (ETF; nrs. 42 and 43) may serve as an electron donor for fatty acid metabolism.

The ability to synthesise RQ provides *R. costata* with the opportunity to transfer electrons from Complex I via Complex II to fumarate, but the same reaction can be performed by FAD-dependent fumarate reductase (nr. 24), which uses ubiquinol for fumarate reduction. The succinate produced is the substrate of succinyl-CoA synthetase producing succinyl-CoA, which may enter the synthesis of wax esters described in *E. gracilis*. *R. costata* contains all enzymes needed for this process. Propionyl-CoA, the first substrate needed for wax ester synthesis, is produced by methylmalonyl-CoA mutase (nr. 27), methylmalonyl-CoA epimerase (nr. 28), and propionyl-CoA carboxylase α and β (nrs. 29 and 30). The condensation of propionyl-CoA and acetyl-CoA can be, in principle, catalysed by acetyl-CoA acyltransferase (nr. 31 and 37) instead of the missing α-ketoacyl synthase. *R. costata* contains 3-hydroxyacyl-CoA dehydrogenase (nr. 32) and enoyl-CoA hydratase (nr. 33), and bifunctional protein enoyl-CoA hydratase / long-chain 3-hydroxyacyl-CoA dehydrogenase (nr. 36) as well as enzymes needed for the reduction of trans-enoyl-CoA: trans-2-enoyl-CoA reductase (nr. 34), acyl-CoA dehydrogenase (nr. 35) and ETF (nrs. 42–43), which can provide electrons. The pathway further proceeds outside the mitochondrion, where carnitine O-palmitoyltransferases 1 and 2 (nrs. 38 and 39) and the carnitine/acylcarnitine translocase (nr. 91) export the acyl-CoA. Neither fatty acyl-CoA reductase (an ER enzyme) nor wax ester synthase (a cytosolic enzyme) was detected; however, 17 transcripts encoding a bifunctional enzyme ester synthase/diacylglycerol acyltransferase (WSD), robustly branching with their orthologues in *E. gracilis*, were detected (Supplementary Fig. S24 online). This enzyme was firstly characterised in *Acinetobacter calcoaceticus*^32^ and later demonstrated as the dominant enzyme for the wax ester synthesis in *Euglena gracilis*^33^. Notably, two of the *E. gracilis* proteins closely related to *R. costata* homologues (BAV82975.1 and BAV82978.1) seem to play pivotal role in this process^33^.

The organelle may be able to import sulphate via a putative transporter (nr. 68), although the identity of this protein is uncertain. It also contains enzymes needed for sulphate activation, sulphate adenylyltransferase (nr. 69) and adenylylsulphate kinase (CysC; nr. 70), to produce phosphoadenosyl-5’-phosphosulphate (PAPS). The enzymes that metabolise inorganic sulphur compounds, thiosulphate sulphur transferase and sulphite oxidase, are present (nrs. 71 and 72); however, the enzymes necessary for the production of sulphide were not detected. Still, transcripts of the sulphide-dependent enzyme, cysteine synthase (nr. 74), are present, as is the L-serine producing serine O-acetyltransferase (nr. 73). The mitochondrion also contains a rich set of enzymes for the early and late synthesis of FeS clusters, including the mitochondrial export system.

The predicted mitochondrial proteome contains enzymes involved in the synthesis and metabolism of 10 proteinogenic amino acids (S, C, T, G, A, V, L, I, Q, and P). A complete glycine cleavage system and serine/glycine hydroxymethyl transferase, which are involved in the folate one-carbon pool, are also present (nrs. 3–6). The set contains 136 entries involved in metabolite and ion transport across membranes, of which 31 are ABC transporters (including Atm1, involved in FeS cluster export, nr. 57) and 75 are mitochondrial carrier family proteins (solute carrier family 25) that cluster into 57 distinct clades (Supplementary Fig. S25 online), 34 of these are sister to homologues in euglenophytes, another 9 to homologues in other euglenozoans.

More than 30 transcripts of proteins putatively involved in protein transport and maturation were detected. These encode four outer membrane proteins—the translocation pore Tom40 (nr. 84), its insertase Tob55 (nr. 77), and Euglenozoa specific proteins Atom69 (nr. 82) and Atom46 (nr. 83)—as well as two distinct homologues of small Tims (nrs. 79 and 80) localised to the intermembrane space, and seven proteins localised in the inner membrane—inner membrane protease subunit 2 (nr. 90), Tim22 (nr. 85), Tim17 (nr. 86), Tim44 (nr. 87), Tim16 (nr. 88), Pam16 (nr. 89), and Oxa1 (nr. 78). Homologues of both subunits of mitochondrial processing peptidase were detected (RCo000876 and RCo039561); however, the β-subunit may also be a part of Complex III. Soluble chaperones, Mge1 (nr. 49) and Hsp60 (RCo005383 and RCo005843), are present. 17 different transcripts for the chaperone Hsp70 were detected of which two (RCo049920 and RCo045932, nr. 50) robustly branch within the mitochondrial clade (Supplementary Fig. S26 online) likely representing the mtHSP70 orthologues.

Finally, we decided to determine the phylogenetic origin of key mitochondrial enzymes known from *E. gracilis*, which were not detected in *R. costata*—namely PDH (letters a-c), bifunctional glyoxylate cycle enzyme malate synthase/isocitrate lyase (MS/ICL, letter d), AOX (letter e), and sulphite reductase (letters g-h). A taxon rich phylogenetic tree for the alpha subunit of the PDH (E1 component) clearly shows that euglenophyte proteins cluster robustly with kinetoplastids, and so represent ancestral forms, which were lost in *R. costata* (Supplementary Fig. S27). The MS/ICL fusion protein has been reported only from *E. gracilis* and *Acanthamoeba castelanii*, while fusion in the opposite orientation is known from *Caenorhabditis elegans*^34,35^. Our searches indicated that euglenophytes share this fusion with three chrysophytes (Ochromonadales sp. CCMP2298, Hydrurales sp. CCMP1899 and Poterioochromonas sp. BG1), and a kinetoplastid (Prokinetoplastina sp. PhF6) and these organisms cluster in both phylogenies constructed from individual MS and ICL parts (Supplementary Figs. S28–S29). The evolutionary history of this enzyme fusion is potentially interesting and likely involved eukaryote-to-eukaryote HGT, but it is hard to draw any strong conclusion based on the current sampling. The AOX enzymes are known for their complex history^36^ and there are two unrelated AOX homologues in Euglenophyta (Supplementary Fig. S30). A richer clade restricted to genus *Euglena*, whose *E. gracilis* members were reported from mitochondrial proteome, branches sister to a large predominantly algal clade. The second smaller clade composed of homologues from *E. gracilis* (enriched in plastid proteome), *E. mutabilis* and *Eutreptiella gymnastica* sits close to kinetoplastid homologues but its sisters are AOX from Phaeophyta and eustigmatophyte *Nanochloropsis*. The latter AOX homologues may represent ancestral type in euglenids, which has been redirected to plastid and also horizontally transferred to species of phaeophytes and eustigmatophytes. The reductive part of the sulphate metabolism composed of phosphoadenosine phosphosulphate reductase, sulphite reductase alpha- (CysJ) and beta-component (CysI) is clearly absent in the *R. costata* data set and the origin of *E. gracilis* enzymes is unclear and likely not vertical from the ancestor of Euglenozoa (Supplementary Figs. S31–S33).

### Thiamine metabolism

**Figure 6:**
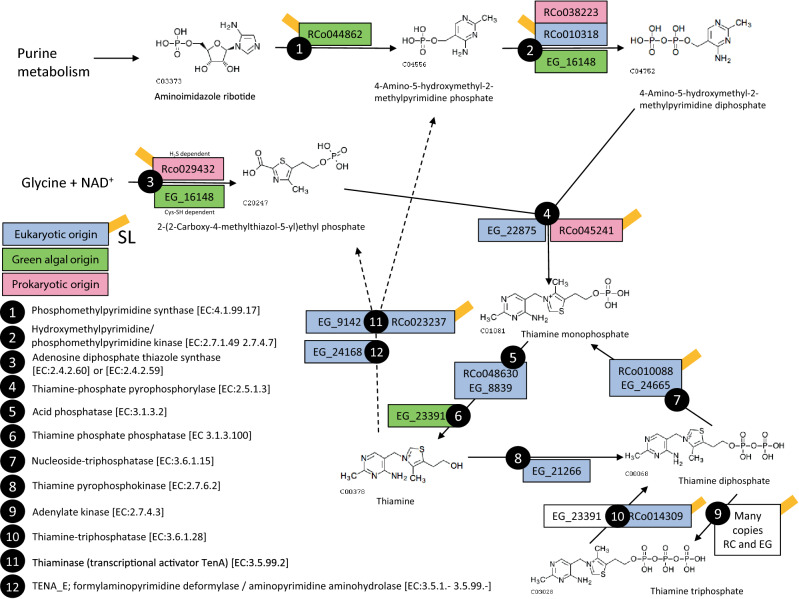
Map of the thiamine metabolism of *R. costata*. The enzymes identified in the transcriptome of *R. costata* are indicated in rectangles above or to the right from the arrows (opposite from their *E. gracilis* homologues). The colour scheme indicates the phylogenetic origin of these enzymes and the presence of SL longer than nine nucleotides is marked by orange rectangles. The pathway map and compound drawings are based on KEGG^23^.

Thiamine diphosphate (TDP) is a cofactor of several essential enzymes of which at least PNO and 2-oxoglutarate decarboxylase are present in *R. costata*. It is known that *E. gracilis* is auxotrophic in thiamine because it is unable to synthesise 4-amino-5-hydroxymethyl-2-methylpyrimidine phosphate, one of the two substrates necessary for its synthesis^37^. We have investigated this pathway in *R. costata* (Fig. 6, Supplementary Table S10 online) and revealed that it unexpectedly does contain phosphomethylpyrimidine synthase catalysing the first reaction absent in *E. gracilis*. The transcript contains euglenid SL and protein branches within eukaryotic clade with weakly supported sister relationship to green algae (Supplementary Fig. S34), therefore it is unlikely a contaminant. Brief inspection of the KEGG metabolic map for purine metabolism indicates that *R. costata* should be capable of synthesis of aminoimidazole ribotide, the substrate for this reaction. As apparent from Fig. 6, the transcriptome data for *R. costata* contain at least partial sequences of enzymes for the whole pathway leading to thiamine monophosphate (TMP) and thiamine (i. e. reactions 1–5 in Fig. 6). Interestingly the pathway is an evolutionary mosaic and besides acid phosphatase and nucleoside-triphosphatase (steps 5 and 7), *R. costata* enzymes are unrelated to that of *E. gracilis* (Supplementary Figs. S6 and S34–S45). Some of them branch with prokaryotes, but the presence of SLs suggests *R. costata* origin of the sequence. A good example of this is adenosine diphosphate thiazole synthase (step 3), which in *E. gracilis* is a cysteine dependent and robustly related to *Pyramimonas parkae* suggesting the origin from plastid endosymbiont (Supplementary Fig. S36), while *R. costata* protein is related to methanogenic archea and uses sulphide as the source of sulphur.

The transformations between thiamine and its phosphates are mostly catalysed by orthologous enzymes in *E. gracilis* and *R. costata*. Interestingly, *R. costata* does not contain thyamine pyrophosphokinase, a major enzyme for TDP production in *E. gracilis*^38^. Furthermore, both species contain nucleoside triphosphatase (reaction 7), which to our knowledge catalyses only the reaction in direction towards TMP, and so it is questionable whether this may provide a way for the TDP synthesis. Obviously, the incompleteness of the transcriptome data set may lead to omission of the enzyme catalysing the final reaction of TDP cofactor synthesis. Finally, *R. costata* as well as *E. gracilis* are capable of recycling thiamine to the substrates of its synthesis by thiaminase (reaction 11).

### Tetrapyrrole synthesis pathways

**Figure 7:**
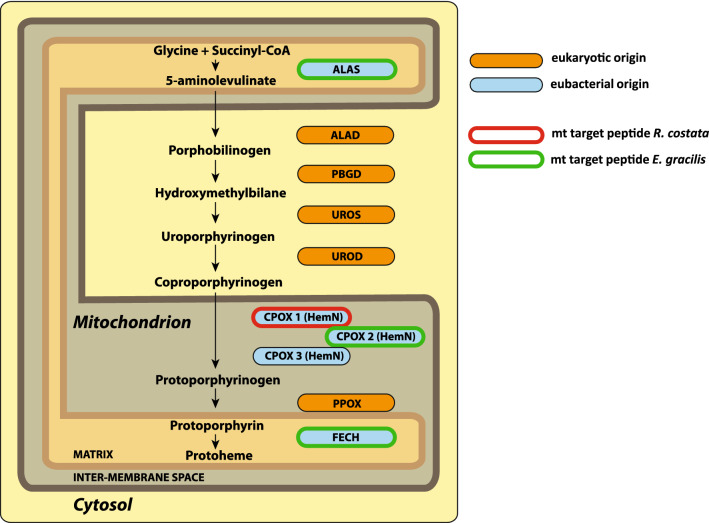
Origins and subcellular localisations of tetrapyrrole synthesis enzymes in *R. costata*. Predicted localisation of the enzyme is indicated by its position in the diagram. Phylogenetic origin is indicated by colour (orange—eukaryotic origin, blue—eubacterial origin). Presence of mitochondrial targeting peptide (TP) is indicated by frame (red frame—high TP value in *R. costata*, green frame—high TP value in euglenophytes).

We found transcripts for the full set of haem biosynthesis enzymes in the transcriptome (Fig. 7). All enzymes formed clades with *E. gracilis* mitochondrial-cytosolic C4 pathway enzymes with various statistical supports and were most likely present in their common ancestor. The mitochondrial 5-aminolevulinate synthase (ALAS; RCo053079) branches within the eukaryotic clade and appears closely related to α-proteobacteria, suggesting a mitochondrial origin (Supplementary Fig. S46 online). Although ALAS should localize in the mitochondrion, *in silico* prediction for this localisation gives probability below the 0.5 threshold. The following four steps take place in the cytosol and the pathway ends in the mitochondrion. Porphobilinogen synthase (ALAD; RCo046560), and porphobilinogen deaminase (PBGD; RCo016092) homologues are closely related to homologues from another bacterivorous euglenid (*Distigma* sp.), and together with the cytosolic isoforms of *E. gracilis*, they branch within eukaryotic genes (Supplementary Figs. S47 and S48 online). *R. costata* uroporphyrinogen synthase (UROS; RCo031923) branches with photosynthetic euglenids, and the clade is weakly supported sister group to oomycetes (Supplementary Fig. S49 online). Although this protein was annotated as a plastidial form in *E. gracilis*, it is probably cytosolic or dual-localized in both the cytosol and plastid. Cytosolic localization is supported by the presence of a second, so far unnoticed and putatively plastidial UROS homolog in the transcriptome of *E. gracilis* (EG_transcript_17485), which robustly branches with green algae and cyanobacteria, though it lacks a clear plastidial targeting signal. The next enzyme, uroporphyrinogen decarboxylase (UROD), has three annotated isoforms in *E. gracilis*, and *Eutreptiella gymnastica*. One of them is plastidial and has not originated from green algae but more likely from cryptophytes. The other two are probably originally cytosolic and branch within the eukaryotic clade. These isoforms seem to originate from an ancient gene duplication, and we found only one isoform (RCo052619) in *R. costata* (Supplementary Fig. S50 online).

The most complicated situation is in the case of coproporphyrinogen oxidase (CPOX). *E. gracilis* has at least eight isoforms. Five of them are plastidial, oxygen-dependent CPOX (HemF), and no homologue was found in *R. costata*. Three other isoforms of *E. gracilis* belong to the phylogenetically distant oxygen-independent CPOX (HemN) clade, where they occupy different positions (Supplementary Fig. S51 online). Two *E. gracilis* isoforms group together with *R. costata* (RCo019415, and RCo045123) homologues and branch within the α-proteobacterial clade, suggesting their origin from HGT. These two isoforms have a clear mitochondrial targeting signal. The third one is more divergent, and the *R. costata* homologue (RCo029168) is not closely related to the *E. gracilis* one, but rather to α-proteobacteria homologues. *R. costata* has several other homologues without specific mitochondrial targeting signal branching at various places in the tree. A putatively mitochondrial isoform of protoporphyrinogen oxidase (PPOX; RCo026828) branches within a well-supported eukaryotic clade together with *Distigma* sp. and *E. gracilis* (Supplementary Fig. S52 online), but the targeting prediction is weak. This contrasts with *E. gracilis,* and *E. gymnastica* PPOX plastid isoforms, which are placed within plastid proteins from various secondary red algae. Lastly, the mitochondrial isoform of ferrochelatase (FECH) is not derived from eukaryotes; instead, euglenid sequences, including the *R. costata* homologue (RCo012206), branch within bacterial clades and were probably obtained by HGT (Supplementary Fig. S53 online). Consistent with the probable absence of a plastid, there are no traces of the plastidial C5 pathway in *R. costata*.

## Discussion

In this work, we contribute to the understanding of nonphotosynthetic euglenids by presenting draft genome and transcriptome assemblies of *Rhabdomonas costata*. Although the genome assembly is very fragmented and incomplete, we demonstrate its usefulness by deducing new information about intron composition in *R. costata* and we were able to reconstruct four pieces of its mitochondrial genome. We believe that the fragmentation is an artefact of uneven whole genome amplification (WGA) in the DNA sample preparation but other reasons (e.g. presence of repeats) cannot be excluded at this point. It should be mentioned that the level of fragmentation is comparable to the published draft genome of *Euglena gracilis*^17^ and the total number of contigs in the assembly is lower in *R. costata* (143,763 vs. 2,066,288). Similar to *E. gracilis*, the *R. costata* transcriptome is richer in GC compared to the genome sequence—58% vs. 51%, respectively. In total, the genome assembly is 106 Mbp in length, which, considering its completeness estimated from transcriptome mapping (43.5%), gives a haploid genome size estimate of approx. 250 Mbp. The analysis based on k-mer frequency predicts genome size 128 Mbp but the prediction may be biased due to uneven amplification of the genome during sample preparation. Although we have only imprecise estimates of the genome size or *R. costata*, they are both smaller than half of the value estimated for *E. gracilis*^17^.

The quality of the transcriptome dataset was sufficient for functional annotation. The number of non-redundant predicted proteins (39,456) is comparable to *E. gracilis* (36,526)^17^ and from the BUSCO measure of completeness (12.5% missing), we infer that the protein set is reasonably complete. Mature euglenid transcripts often contain splice leader (SL) sequences acquired by trans-splicing^24^. However, it is likely that not all transcripts require SL for successful translation^39^ and in the transcriptomes of *E. gracilis*, and *E. longa*, only 54% and 48.5% of transcripts, respectively, have been reported to contain at least a fragment of SL^27,40^ somewhere in their sequence. This could be to some extent caused by truncation of the N-termini. We applied a stricter rule for SL identification, in which the SL was only searched for by an exact match at ends of the transcripts. This search obviously revealed a lower fraction of SL-containing transcripts (Supplementary Table S1 online), but the values for *R. costata* (e.g. 15.3% transcripts with SL longer than 9 nucleotides) were within the range of other euglenid transcriptomes.

Almost all eukaryotic genomes, including that of *E. gracilis*, contain introns that are removed by the ribonucleoprotein complex, the spliceosome. These conventional introns have the consensus sequence GT(GC)/AG at their ends, and they are excised by two sequential transesterifications. They have been described in *E. gracilis* in genes encoding 13 proteins^25,39,41–45^. However, the genes of euglenids also contain nonconventional introns variable in length and with no clear pattern of the nucleotide sequence at the exon/intron junction. They form the stem-loop RNA structures, and their excision is probably independent of the spliceosome, taking place after the excision of spliceosomal introns^25,46^. Besides these main types of introns, so-called intermediate introns that combine the features of both types have been reported^25^.

The genome of *R. costata* seems to be relatively intron rich. We have confirmed the presence of introns in all genes for which introns have been investigated in other euglenids^25,39,41,43^, and our automatic search for introns in the fragmented genome assembly revealed hundreds of putative introns. Seven of these introns are likely nonconventional. The presence of nonconventional introns in *R. costata* is expected, as they have been reported from several euglenid species^25^ and from a marine diplonemid^11^.

The phylogenetic affiliation of predicted *R. costata* proteins is similar to that assessed for *E. gracilis*^17^, with the notable but expected difference in the fraction of genes affiliated with Viridiplantae. The fraction is much higher in *E. gracilis* than in *R. costata* (14% as compared to 5%) and reflects the symbiotic history of *E. gracilis*, the plastid of which originated from a green algal endosymbiont*.*

By manual curation we were able to extract four contigs from the genomic assembly that very likely represent parts of the mitochondrial genome. These contigs encode cox1, cox2, cytb and nad4 genes known also from the mitochondrial genome of *E. gracilis*^16^. In silico translation of these proteins and their alignment with homologues in other Euglenozoa suggests that the mitochondrion of *R. costata* uses a rare variant of genetic code assigning UGA stop codons to tryptophan like in *E. gracilis* but also UAG stop codons to tyrosine. The latter may relate to the fact that the inventory of tyrosine-tRNA synthetases of *R. costata* is different from that of *E. gracilis*. Reassignment of UAG to tyrosine is relatively rare and was reported recently from mitochondria of some labyrinthulids^47^. The combination of UGA encoding tryptophan and UAG encoding tyrosine was reported to our knowledge only from poriferan *Clathrina clathrus* in which it is combined with reassignment of CGN codons to glycine^48^.

The *in silico* predicted proteome of the *R. costata* mitochondrion is smaller in size than the experimental proteome of *E. gracilis*—1554 in *R. costata* vs. approx. 2,500 in *E. gracilis*^22^. This difference may be real, but it may also reflect the data set completeness and/or the procedure used to generate the set of proteins in the mitochondrial proteome. Direct orthology comparison showed that, of the 1782 experimentally verified proteins of the *E. gracilis* mitochondrion^22^, 1083 have at least one orthologue in *R. costata,* and in total, 1606 *R. costata* proteins are orthologous to this set. The mitochondrion of *E. gracilis* bears a unique combination of metabolic features. It contains a set of enzymes for the facultatively anaerobic metabolism that, in the presence of oxygen, metabolises pyruvate or malate by the pyruvate dehydrogenase complex, followed by a slightly modified TCA cycle, and then the full set of respiratory complexes, including the alternative oxidase also described from kinetoplastids^49^. In the absence of oxygen, pyruvate is oxidatively decarboxylated by pyruvate:NADH oxidoreductase (PNO), and the mitochondrial NADH is recycled by respiratory Complex I and rhodoquinone-dependent fumarate reductase, producing succinate, which is used for the synthesis of propionyl-CoA. The latter is condensed with acetyl-CoA into fatty acids and wax esters that are stored in the cytoplasm at high concentrations^50^. These are recycled under aerobic conditions for ATP production or for the synthesis of organic compounds through a functional glyoxylate cycle, uniquely localised in the mitochondrion^51^.

The biochemistry of the *R. costata* mitochondrion resembles in several aspects that of the *E. gracilis* organelle, but it seems to be more streamlined. It contains a complete TCA cycle with the euglenid-specific bypass of 2-oxoglutarate decarboxylase. It also uses rhodoquinone to reverse the electron flow under low oxygen conditions to fumarate as the final electron acceptor, and the produced succinate is then consumed during the synthesis of wax monoesters. Unlike *E. gracilis*, the *R. costata* mitochondrion uses only PNO for oxidative decarboxylation of pyruvate, and it does not contain an alternative oxidase in its electron transport chain, which is consistent with our inability to amplify these transcripts by PCR. Intriguingly, the *R. costata* mitochondrion does not contain enzymes of the glyoxylate cycle, a shortcut of the TCA cycle, which is used to generate four-carbon molecules from the acetyl-CoA released after the degradation of lipids and wax esters. How this is solved in *R. costata* remains to be elucidated. Phylogenetic analyses of the enzymes present in *E. gracilis* but absent from *R. costata* mitochondrion suggest that while PDH complex was ancestrally present in euglenids and subsequently lost in *R. costata*, MS/ICL protein of glyoxalate cycle has been acquired by euglenophytes from unknown source. The phylogenetic history of AOX is complicated and suggests switches of localisations between mitochondria and plastids.

An interesting difference between *E. gracilis* and *R. costata* lies in sulphate metabolism. *E. gracilis* is apparently capable of assimilating sulphate into cysteine in the mitochondrion^22,52^ and sulphite reductase has been detected in its chloroplast fraction^21^. In contrast, *R. costata* can only activate sulphate to the form of PAPS, an important coenzyme in sulphotransferase reactions, but the enzymes necessary for the internal production of sulphide from PAPS, were not detected. Still, the presence of sulphide-dependent cysteine synthase suggests that *R. costata* may be able to synthesise cysteine from sulphide putatively produced from PAPS by unclear mechanism or taken up from the anaerobic environment.

TDP is a cofactor of several essential enzymes many of them being localised in the mitochondrion. Unlike *E. gracilis* that needs to uptake thiamine from the environment for its growth, *R. costata* is probably capable of its synthesis. Enzymes of the synthetic pathway compose an evolutionary mosaic, adenosine diphosphate thiazole synthase is again and unusually for a eukaryote sulphide dependent but the presence of SLs supports they originate from *R. costata* transcriptome rather than from contaminants. The enzyme catalysing the phosphorylation step to TDP is uncertain as is the localisation of the whole pathway of which only tree enzymes qualified into predicted mitochondrial proteome. No known thiamine transporter has been detected.

Haem ranks among the essential cofactors in cellular metabolism because it is involved in many key biochemical processes. In most heterotrophic eukaryotes, the haem synthesis C4 pathway involves eight steps that are localised partially in the mitochondrion and partially in the cytosol. The first step is the condensation of glycine and succinyl-CoA into 5-amino-levulinate by ALAS in the mitochondrial matrix. The next four (or five) steps take place in the cytosol. The pathway ends in the mitochondrion with two reactions that take place in the intermembrane space, and the final reaction occurring in the mitochondrial matrix^53^. In most eukaryotes with a plastid, an alternative C5 pathway is present, and all steps are localised in the plastid. In this case, 5-amino-levulinate is synthesised from glutamic acid by three consecutive enzymes: glutamyl-tRNA synthetase (GltX), glutamyl-tRNA reductase (GTR), and glutamate-1-semialdehyde 2, 1-aminomutase (GSA-AT). The pathway then follows the same steps as the classical pathway, but it is localised in the plastid and catalysed by enzymes of mostly cyanobacterial origin^54,55^. Unexpectedly, *E. gracilis* and the chlorarachniophyte *Bigelowiella natans* (i.e. algae with complex plastids that originated from green algae) have both pathways, the mosaic evolutionary origin of their enzymes reflecting the complex evolutionary history of these eukaryotes^54,56–58^.

The *R. costata* transcriptome contains a complete set of enzymes of the mitochondrial/cytosolic C4 pathway that are orthologous to *E. gracilis* enzymes. Comparing these two euglenids helped to reveal that one UROS and two UROD isoforms of *E. gracilis* examined so far are probably cytosolic C4 pathway enzymes, as they all have *R. costata* orthologues. Interestingly, we recovered an unnoticed UROS homologue from the *E. gracilis* transcriptome related to green algae and putatively involved in the C5 pathway in its plastid. While several plastidial isoforms of the oxygen-dependent CPOX (HemF) in *E. gracilis* have two distinct origins in the eukaryotic kingdom, the newly discovered isoforms of the oxygen independent CPOX (HemN) that function in the mitochondrion have α-proteobacterial origin. The complete absence of the plastidial C5 pathway for tetrapyrrole synthesis illustrates the overall limited number of transcripts affiliated to green algae, which is consistent with the absence of a chloroplast in *R. costata* and supports the osmotrophic lifestyle of this euglenid as its primary state.

## Methods

### Strain origin and cultivation

*Rhabdomonas costata* strain PANT2 was isolated from a freshwater sediment sample collected ca 40 km south of Poconé municipality, Mato Grosso, Brazil (16°37′S, 56°44′W) and grown in monoeukaryotic culture together with a non-characterised mixture of bacteria in a standard 802 Sonneborn's *Paramecium* medium (ATCC medium #802) at room temperature and subcultured approximately once every 3 or 4 weeks. For the purpose of this project, we prepared a presumably clonal lineage by serial dilution that was used for DNA and RNA extraction.

### Microscopy

The DIC microscopy was performed on living cells, using an Olympus BX51 microscope with an Olympus DP70 camera. For the scanning electron microscopy, the pelleted cells were dropped on filter paper and fixed with 2.5% glutaraldehyde in 0.1 M cacodylate buffer for 24 h. Further processing was done by a service laboratory. The samples were observed using a JEOL JSM-6380LV microscope (JEOL, Akishima, Japan). For transmission electron microscopy, the pelleted cells were fixed in 2.5% glutaraldehyde in 0.1 M cacodylate buffer, postfixed with OsO_4_ and the ultrathin sections were contrasted by uranyl acetate and observed using a JEOL JEM-1011 microscope.

### Isolation of DNA

We used two methods to obtain high-quality, non-contaminated DNA of *R. costata*. (1) The culture cells were sorted by FACS with the value of the drop diameter equal to 70 µm (length of the cell is around 25 µm). Around 2000 positive drops, most of them containing a single cell of *R. costata,* were collected and used for DNA extraction. (2) By a combination of FACS and laser microdissection, in which 69 drops from FACS were subsequently used for microdissection of individual cells, from which DNA was extracted. Both samples were then subjected to whole genome amplification (WGA; Sigma-Aldrich WGA4-10Rxn) to increase the amount of DNA. After amplification, the samples contained 27.7 µg and 32.0 µg of DNA, respectively. PCR with general prokaryotic primers for 16S rRNA had produced a specific product when sample 1 was used as a template, but no product with sample 2 (not shown). This supported our expectation that while sample 1 is still contaminated by bacterial DNA, sample 2 is likely contamination-free.

### Genome sequencing and assembly

Both samples were sequenced on the Illumina platform. Initially, two MiSeq runs of sample 2 (l = 250 bp) produced 3.9 Gbp of sequences. Unfortunately, we were not able to produce a reasonable assembly from these data. Therefore, we also sequenced sample 1 using HiSeq (l = 100 bp) and obtained 8.2 Gbp of data. The raw reads from the sequencing of both samples were assembled by SPAdes v3.7.0 into 143,763 contigs (36,105 contigs longer than 1000 bp). Mapping of genomic reads to the genome sequence was done with bowtie2^59^. The coverage estimation based on k-mer frequencies was estimated with khmer^60^. Extension of mitochondrial genomic contigs has been done by read mapping and manual curation using Geneious Prime 2020.2.2.

### RNA isolation, transcriptome sequencing and assembly

RNA was isolated three times using three slightly different approaches from three different specimens of our *R. costata* clonal lineage: (1) Direct isolation of mRNA (Dynabeads mRNA Direct Kit, including polyA-selection; Thermo Fisher Scientific, Waltham, MA, USA) from which the library was prepared, including a polyA-selection step; (2) Isolation of total RNA (GeneAll Hybrid-R RNA purification kit; GeneAll Biotechnology, Seoul, South Korea) followed by mRNA purification (Dynabeads mRNA Direct Kit) from which the library was prepared, including a polyA-selection step; and (3) Isolation of total RNA (GeneAll Hybrid-R RNA purification kit) and from 4 µg of the total RNA, the library was prepared according to the standard TruSeq Stranded mRNA Sample Preparation Guide including a polyA-selection step. Unlike the first two approaches, the latter approach included only one step of polyA selection. The total RNA was always quantified using a Quantus Fluorometer (Promega, Madison, WI, USA) and its quality was checked with an Agilent Bioanalyzer (Agilent Technologies, Santa Clara, CA). All three samples were sequenced on an Illumina MiSeq instrument (Illumina, San Diego, CA, USA) using 150 base-length read chemistry in the paired-end mode. As no principal differences in bacterial contaminations among the three libraries were observed, they were assembled with Trinity v2.0.6 using default parameters and 93,852 contigs were created. We used Transdecoder for basal protein prediction and obtained 55,783 putative proteins. The software package CD-HIT v.6^61^ was applied with the default threshold 90% to remove redundancy. The set was further partially decontaminated by removing proteins with the highest similarity to bacteria *Tolumonas auensis*, strain DSM 9187. Another set of probable contaminants was filtered out based on the GC content and codon adaptation index CAI^62^. In both cases we took advantage of the fact that using phylogenetic pipeline described below, we have selected from the transcriptome sets of 2718 and 441 transcripts with robust eukaryotic and prokaryotic affiliations, respectively. GC content was calculated for the coding sequences of these transcript and compared. Histogram in Supplementary Fig. S54 online shows that GC content of transcripts with eukaryotic affiliation does not drop below 0.45, unlike those with prokaryotic affiliation which likely contain contaminants. CAI for each transcript was evaluated using a reference codon usage table calculated from 2718 transcripts with eukaryotic affiliation. All calculations have been performed on Galaxy platform (usegalagy.org). Similarly to the GC content, histogram in Supplementary Fig. S55 online shows that CAI of transcripts with eukaryotic affiliation does not drop below 0.45. From this benchmarking we assume that transcripts with GC or CAI below 0.45 are probable contaminants. We applied the most conservative approach and removed 129 transcripts which dropped in both measures under the 0.45 threshold. None of these transcripts had a eukaryotic hit on NCBI with e-value below 10^−5^. The final data set after decontamination contained 39,456 non-redundant amino acid sequences.

### Prediction and characterisation of introns

Introns were automatically predicted by mapping the assembled transcripts to genome contigs using Exonerate (version est2genome 2.2.0). 1164 introns with predicted nonconventional boundaries were selected. Transcript reads were mapped to these contigs by STAR, resulting in 105 contigs in which nonconventional boundaries were supported. Out of these, 26 contigs have good and well-annotated hits on NCBI (e-value < 10^−5^) and for these contigs the intron boundaries were manually inspected. The RNA secondary structures were drawn using Varna 3.9^63^ and logo by WebLogo^64^.

For selected genes (*tubA*, *tubB*, *tubG*, *hsp90*, *gapC* and *nop1p*), the introns were checked by aligning transcripts to genome contigs. The position of these introns was compared to other euglenids.

### Phylogenetic ancestry of *R. costata* proteins

Ancestry of proteins was assessed with the same methodology as in Ebenezer et al.^17^. Briefly, homologues with e-value < 10^−2^ were retrieved from a custom database containing 207 taxa (additional file 3 in Ebenezer et al.^17^), aligned by MAFFT 7.273 with default parameters^65^ and trimmed in trimAl 1.2 with default parameters^66^. 13,696 alignments with more than 3 taxa and longer than 74 amino acid residues were used for tree reconstruction in RAxML v8.1.17 with 100 rapid bootstraps^67^ in Metacentrum (The National Grid Infrastructure in the Czech Republic). Custom scripts (Python 3.7) were used to sort the trees into bins based on the taxonomic affiliation of the clan in which *R. costata* branched. In 3,129 cases, the tree was included in a taxonomically uniform bin because it contained a bipartition supported by bootstrap 75 or higher comprised of *R. costata* and members of only one other defined taxonomic group.

### Prediction of the mitochondrial proteome

The proteome of the mitochondrion was predicted using the following procedure. (1) The complete set of proteins predicted from the transcriptome was BLAST-searched against the MitoMiner database, and 7,985 proteins with e-value < 10^−4^ were selected. To lower the redundancy, only the best *R. costata* hit for each protein included in the MitoMiner database was kept, yielding a set of 1,501 proteins. For every protein, the probability of mitochondrial targeting was predicted using TargetP^68^ and MitoFates^69^ tools. Only proteins with probability of targeting equal to or higher than 0.5 in one or both tools were kept, producing 265 candidates. (2) This initial set was enriched by *R. costata* orthologues of proteins enriched in the mitochondrial fraction of *E gracilis*^22^. Orthology was established using the OrthoMCL software package ^70^. In total, 1,275 proteins fulfilled this criterion, 122 of which were included in the previous step. (3) An additional 121 proteins were included in the list as their presence in mitochondria is very likely—those that are typically part of the respiration chain and protein import complexes, as well as mitochondrial carrier family proteins (Solute carrier family 25). For every gene, the presence of a partial splice leader (SL) at the 5’ terminus and affiliation to prokaryotes in the custom database was established. All proteins mentioned above contained either a partial SL or their best hit was a eukaryote; 179 putative candidates that did not fulfil these criteria were removed during the process of candidate selection. For each entry, the KEGG ID was assigned using the single-directional best hit method in KAAS^71^ or transferred from *E. gracilis* homologue annotation, and the best BLAST hit was identified using blastp against the NCBI nr database. The final predicted mitoproteome consists of 1,538 proteins.

The conspicuous absence of an alternative oxidase in the dataset was verified by gene-specific PCR using degenerate primers based on sequence information from *E. gracilis* (forward primer: GARGARGCNGARAAYGARAGRATGCA; reverse primer GCRAANGTRTGRTTNACRTSNCGRTG), with *E.* *gracilis* and *Eutreptiella gymnastica* gDNAs as positive controls.

### Phylogenies

The partial sequence for the 18S rRNA gene was amplified using primers EPA-23 (5’- GTCATATGCTTYKTTCAAGGRCTAAGCC-3’), and EPA-2286 (5’- TCACCTACARCWACCTTGTTACGAC -3’) according to Müllner et al.^72^ and sequenced using internal primers. Phylogenetic trees of the gene for 18S rRNA and proteins of interest were generated by the following procedure. *R. costata* 18S rRNA/protein(s) and their homologs were downloaded from databases (NCBI and/or EukProt). In the case of the tetrapyrrole biosynthesis pathway, the dataset of Lakey and Triemer^58^ was used as the seed and enriched by *E. gracilis* and *R. costata* transcripts as well as their best NCBI blast hits and the best hits from the local database. All entries were aligned by MAFFT^65^, using the automated strategy, automatically trimmed (BMGE^73^ or TrimAl^66^), and manually inspected. The phylogeny, including 1,000 ultrafast bootstraps, was inferred in IQ-TREE 2.0^74^ using the BIC best-selected model (specified in the legends). Scripts were used in some cases to semi-automatise the process.

## Supplementary Information

Supplementary Figures S1-S4.

Supplementary Figures S5-S23.

Supplementary Figures S24-S33.

Supplementary Figures S34-S45.

Supplementary Figures S46-S53.

Supplementary Figures 54-S55.

Supplementary Tables S1-S10.

## Data Availability

The transcriptomic and genomic reads are available in GenBank under the BioProject ID PRJNA550357, the assemblies and predicted proteins are available in the Zenodo repository (https://zenodo.org/record/4683932#.YHXB0ugzZPY). The sequence of the gene for 18S rRNA is deposited under GenBank accession nr. MW113742. The mitochondrion genome contigs are available under GenBank accession numbers MW837143-6.
