# Supplementary material for: Heterotrophic euglenid Rhabdomonas costata resembles its phototrophic relatives in many aspects of molecular and cell biology
Source: Sci Rep. 2021 Jun 22;11:13070. doi: 10.1038/s41598-021-92174-3 (PMC8219788; doi:10.1038/s41598-021-92174-3)
Supplement: Supplementary file 1 — Supplementary Figures S1-S4. [file 41598_2021_92174_MOESM1_ESM.pdf]

Fig. S1: KEGG Functional categories of predicted proteins of *R. costata*. 9,430 proteins (24 % of all) were ascribed to functional categories according to KEGG with some modifications.

Fig. S2: Comparison of the positions of introns in tubulin α and β genes in four euglenids. Heterotrophic euglenids *Rhabdomonas costata* and *Menoidium bibacillatum* (orange lines) and phototrophic euglenids *Euglena gracilis* and *Euglena agilis* (green lines). Introns shared between heterotrophs – orange ellipses, phototrophs – green ellipses, all four euglenids – brown ellipse. The figure was created in Geneious Prime 2020.2.2.

Fig. S3: Alignments of cox1, cox2, cytb and nad4 genes encoded in the contigs originating from the mitochondrial genome with their homologues in Euglenozoa. Black stars in the *R. costata* sequence indicate stop codons translated using UAG stop codons according to translation table 4. The figure was created in Geneious Prime 2020.2.2.

Fig. S4: KEGG functional categories of proteins predicted to the mitochondrial proteome of *R. costata*. 1,017 proteins of the mitochondrial proteome were ascribed to functional categories according to KEGG with some modifications.

Fig. S1

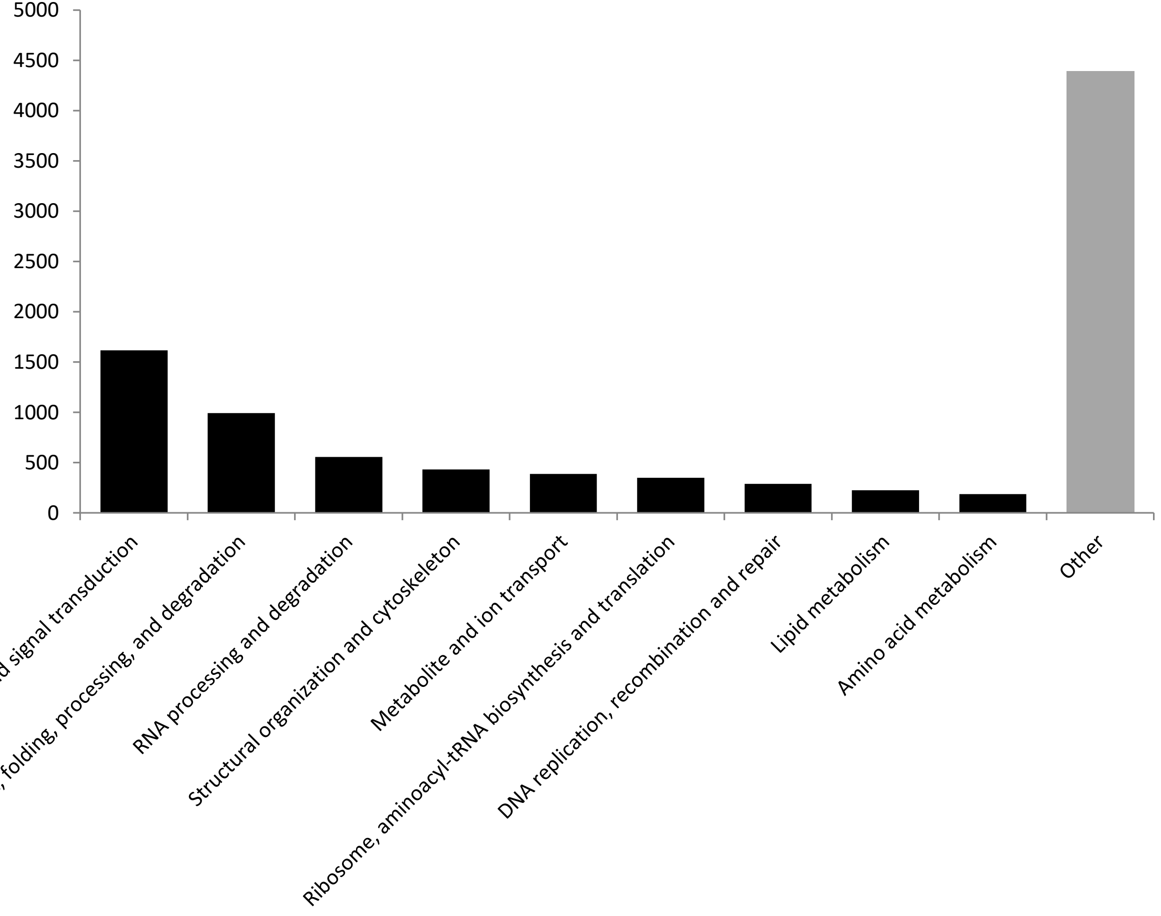
5000
4500
4000
3500
3000
2500
2000
1500
1000
500
0
Regulation and signal transduction
Protein transport, folding, processing, and degradation
RNA processing and degradation
Structural organization and cytoskeleton
Metabolite and ion transport
Ribosome, aminoacyl-tRNA biosynthesis and translation
DNA replication, recombination and repair
Lipid metabolism
Amino acid metabolism
Other

Fig. S2

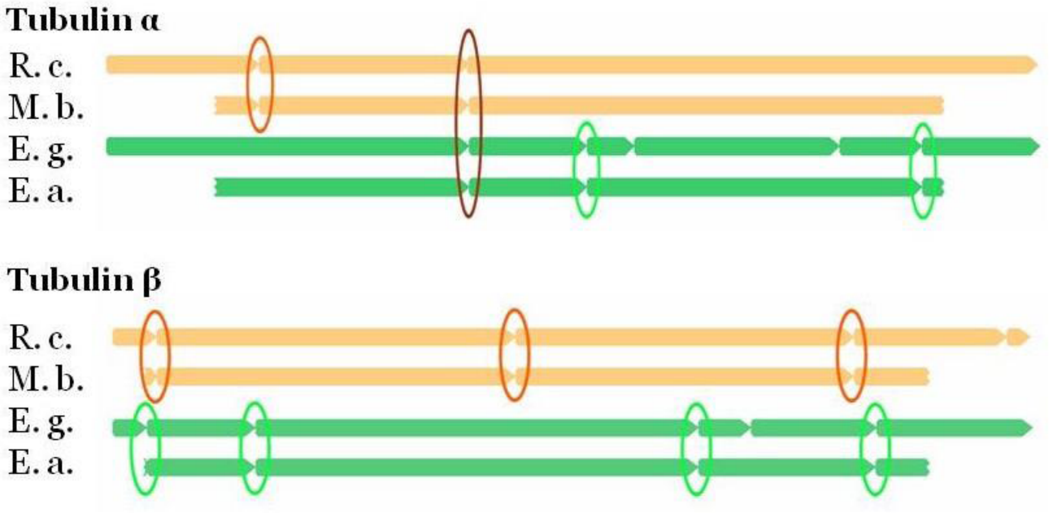

Fig. S3a

Cytochrome c oxidase subunit 1 (mitochondrion)

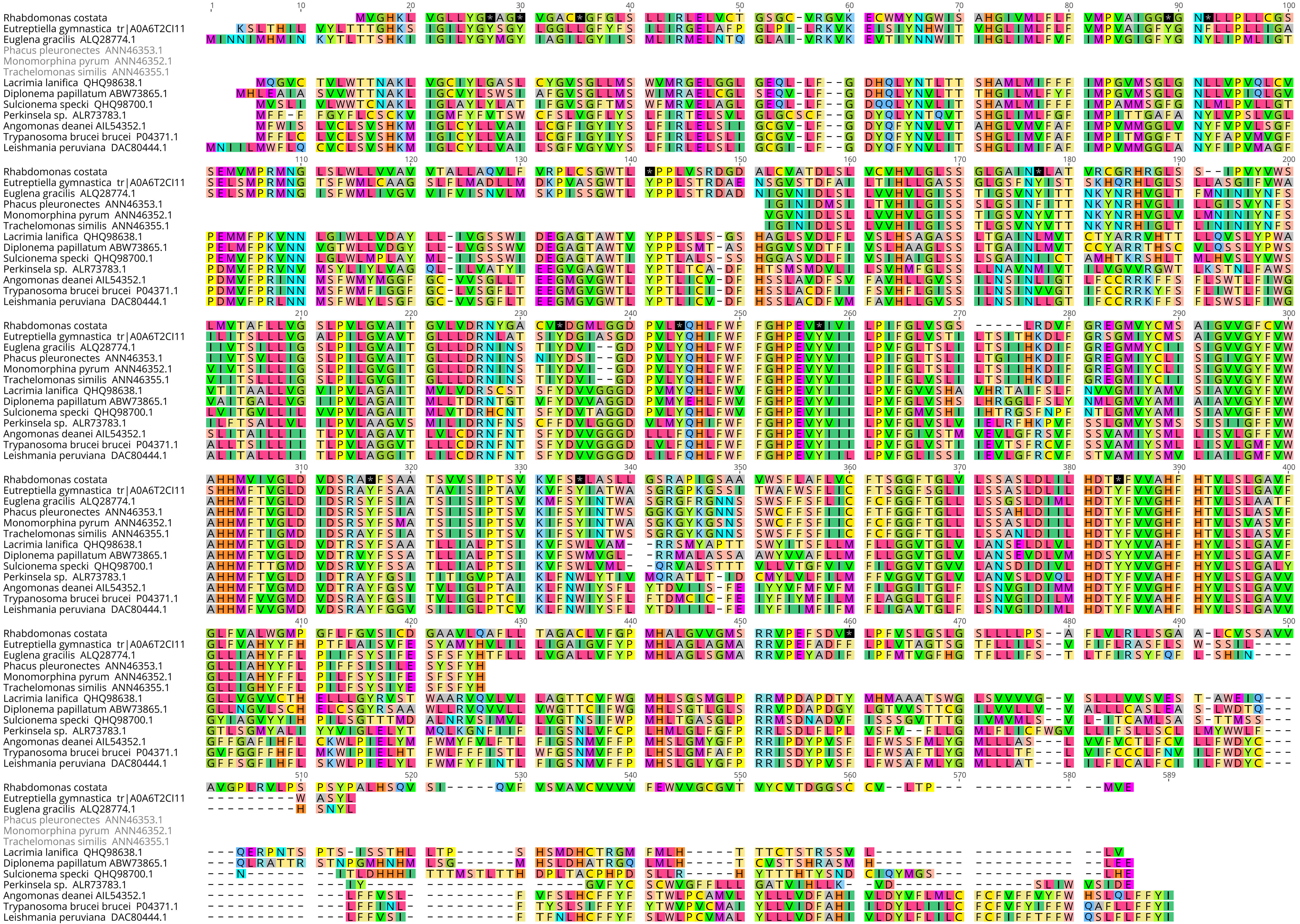

Fig. S3b

Cytochrome c oxidase subunit 2 (mitochondrion)

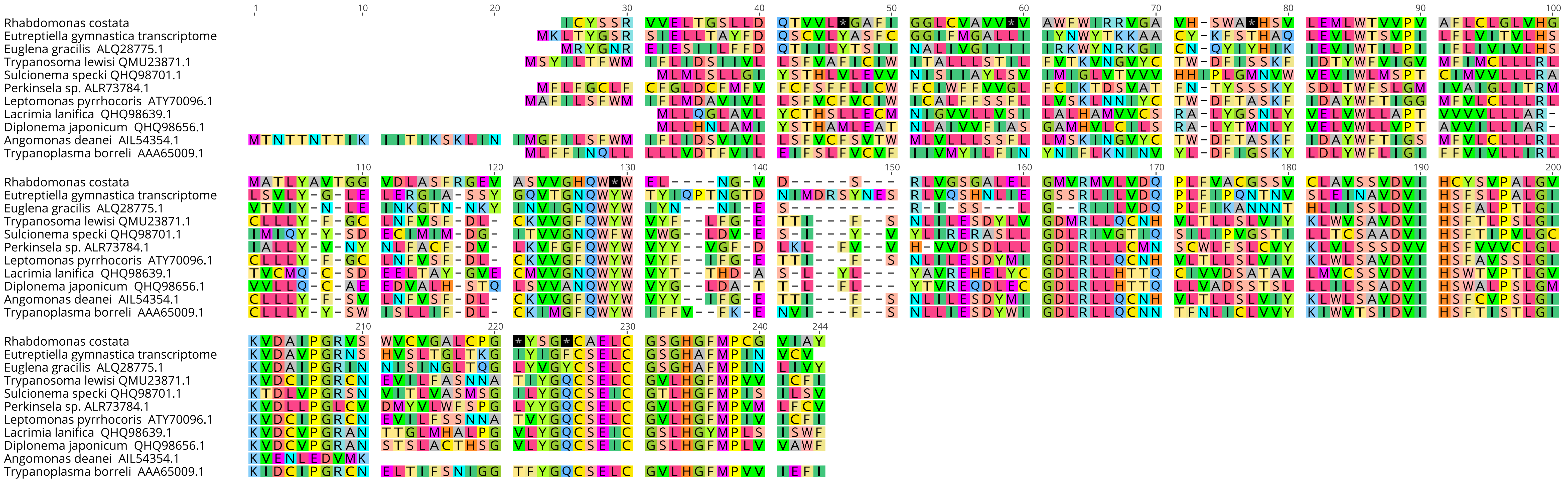

Fig. S3c

Cytochrome b (mitochondrion)

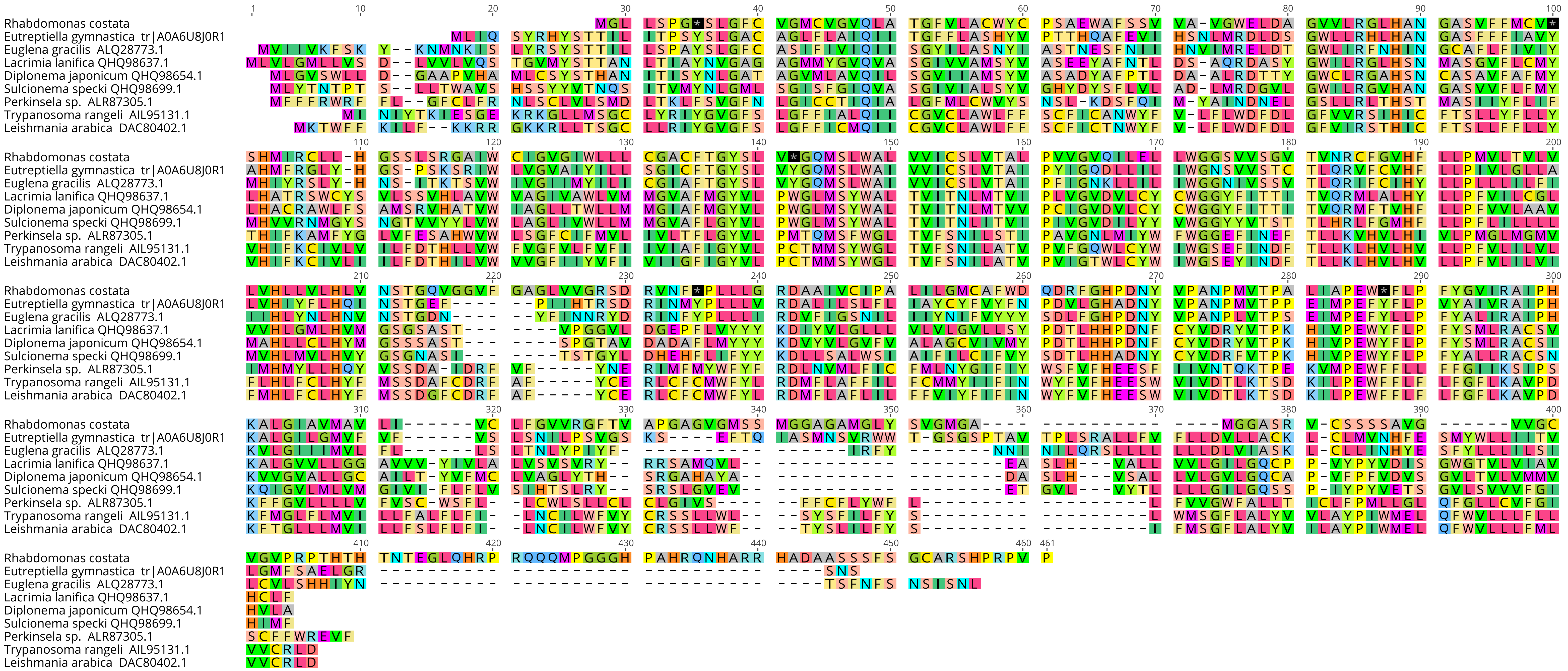

Fig. S3d

NADH dehydrogenase subunit 4 (mitochondrion)

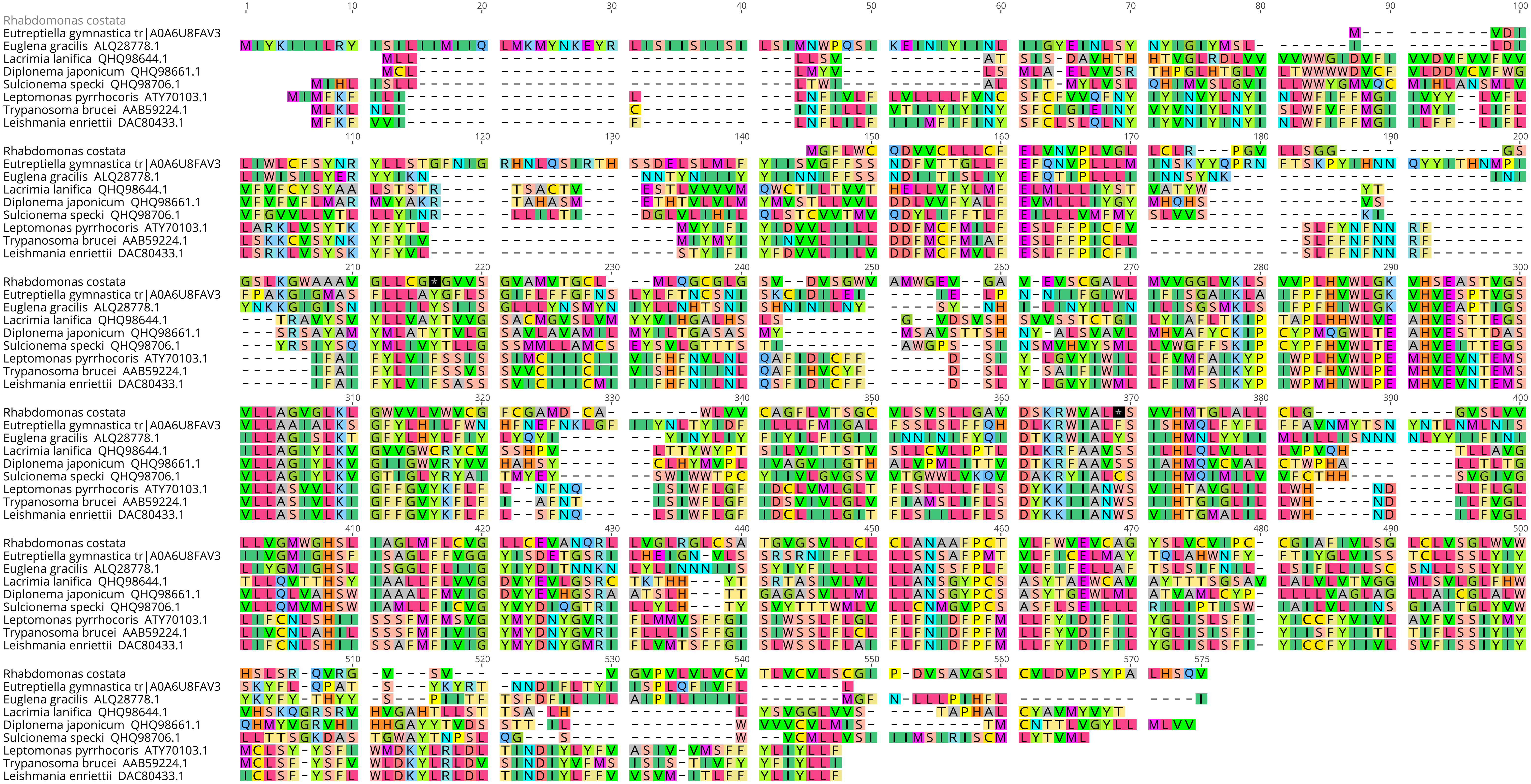

Fig. S4

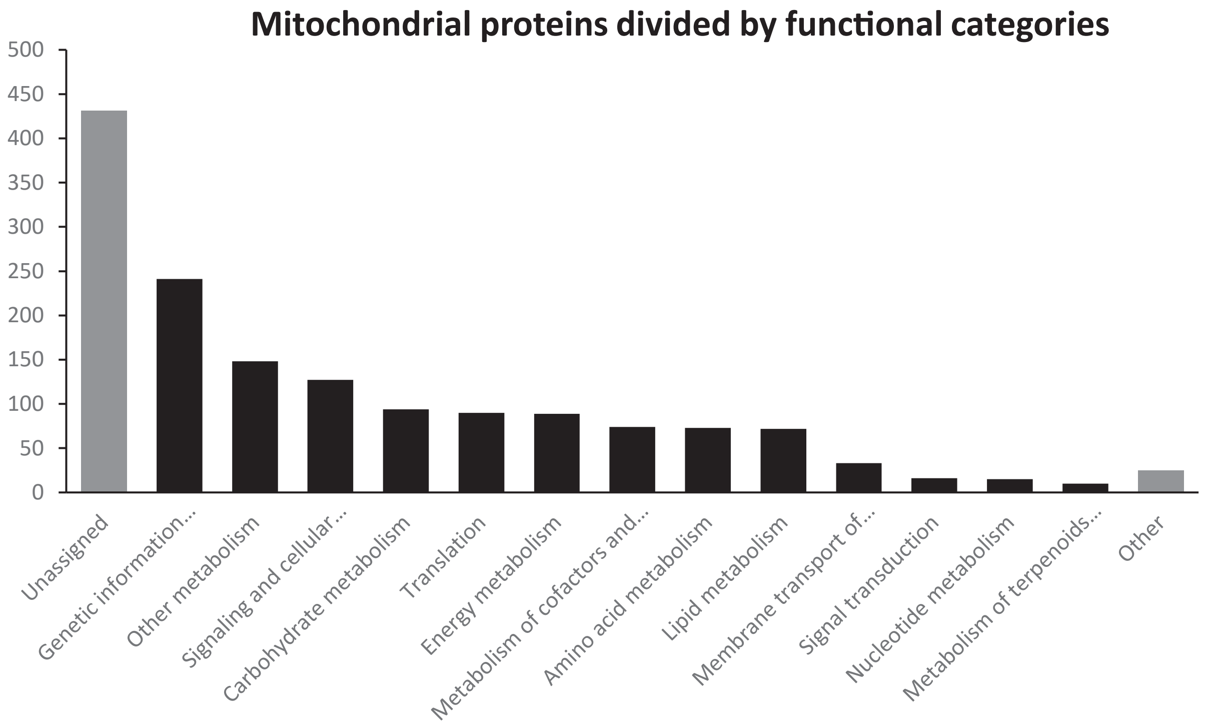
Mitochondrial proteins divided by functional categories
500
450
400
350
300
250
200
150
100
50
0
Unassigned
Genetic information...
Other metabolism
Signaling and cellular...
Carbohydrate metabolism
Translation
Energy metabolism
Metabolism of cofactors and...
Amino acid metabolism
Lipid metabolism
Membrane transport of...
Signal transduction
Nucleotide metabolism
Metabolism of terpenoids...
Other
