# Supplementary material for: Heterotrophic euglenid Rhabdomonas costata resembles its phototrophic relatives in many aspects of molecular and cell biology
Source: Sci Rep. 2021 Jun 22;11:13070. doi: 10.1038/s41598-021-92174-3 (PMC8219788; doi:10.1038/s41598-021-92174-3)

Figs. S5-S23: Phylogenetic trees of aminoacyl-tRNA synthetases. The tree of *R. costata* and homologues from NCBI and EukProt database (https://www.biorxiv.org/content/10.1101/2020.06.30.180687v1) was constructed in IQ-TREE using the model finder-selected model from a TrimAl-trimmed and manually inspected alignment. The values at the nodes represent ultrafast bootstraps from 1,000 repetitions. Euglenophyte sequences are shown in green, *Rhabdomonas costata* sequences in blue, other euglenozoa in red. The supplementary figures are referred in Supplementary table S9. Please note that the sequence ID numbers starting EP0… are arbitrary and do not correspond to the transcriptome database of respective species. The figure was created in FigTree v 1.4.4.

Fig. S5: Alanyl-tRNA synthetase

Fig. S6: Arginyl-tRNA synthetase

Fig. S7: Asparaginyl-tRNA synthetase

Fig. S8: Aspartyl-tRNA synthetase

Fig. S9: Cysteinyl-tRNA synthetase

Fig. S10: Glutamyl-tRNA synthetase/glutaminyl-tRNA synthetase

Fig. S11: Glycyl-tRNA synthetase

Fig. S12: Histidyl-tRNA synthetase

Fig. S13: Isoleucyl-tRNA synthetase

Fig. S14: Leucyl-tRNA synthetase

Fig. S15: Lysyl-tRNA synthetase

Fig. S16: Methionyl-tRNA synthetase

Fig. S17: Phenylalanyl-tRNA synthetase

Fig. S18: Prolyl-tRNA synthetase

Fig. S19: Seryl-tRNA synthetase

Fig. S20: Threonyl-tRNA synthetase

Fig. S21: Tryptophanyl tRNA synthetase

Fig. S22: Tyrosyl-tRNA synthetase

Fig. S23: Valyl-tRNA synthetase

Fig. S5: Alanyl-tRNA synthetase

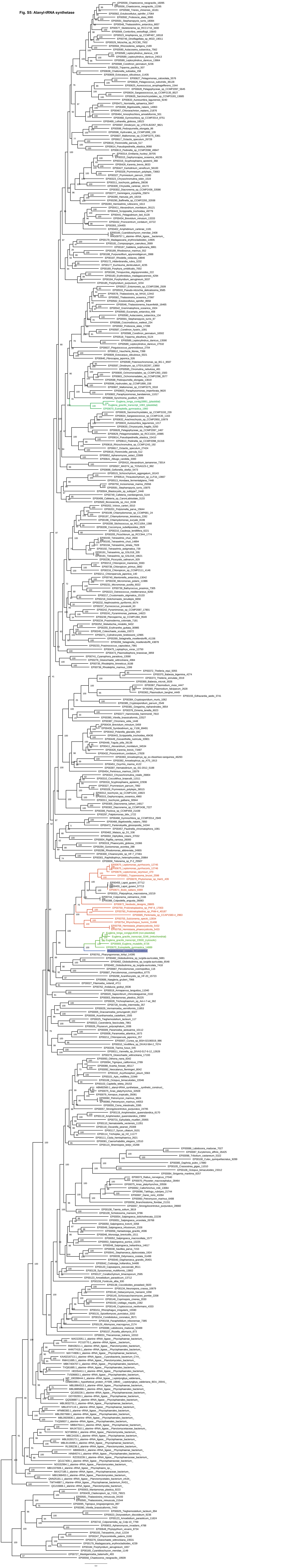

Fig. S6: Arginyl-tRNA synthetase

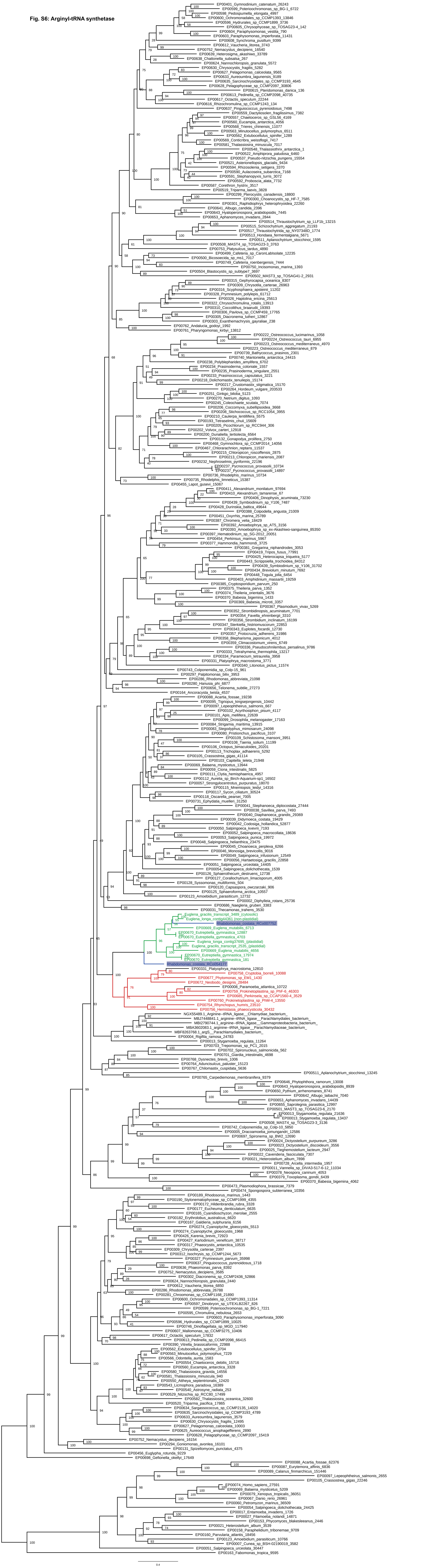

Fig. S7: Asparaginyl-tRNA synthetase

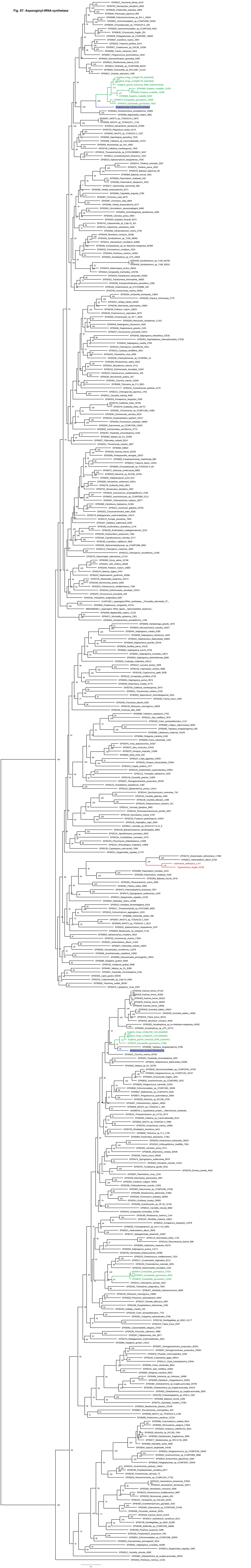

Fig. S8: Aspartyl-tRNA synthetase

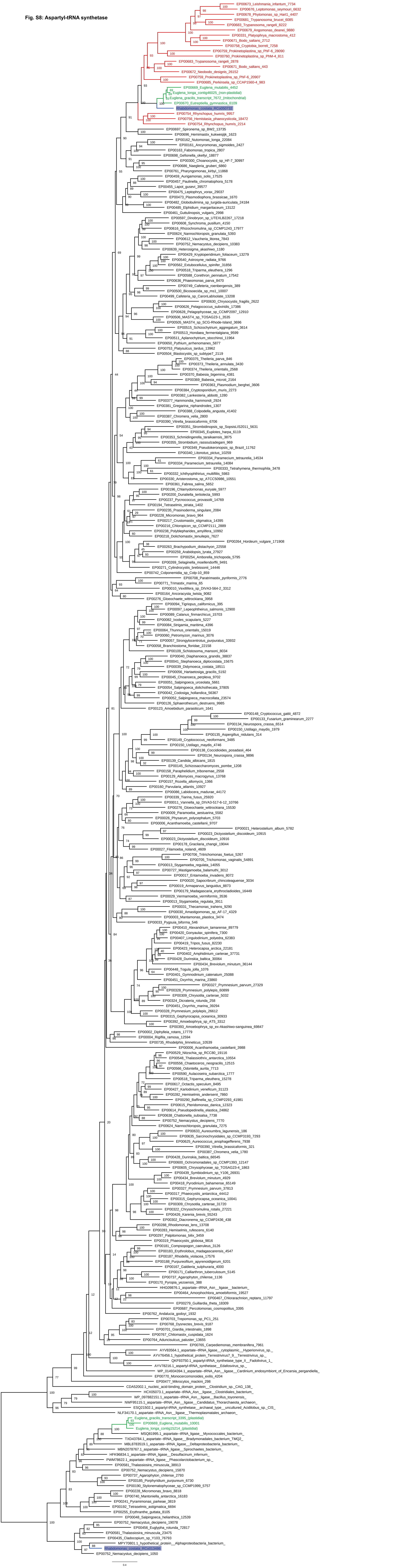

Fig. S9: Cysteinyl-tRNA synthetase

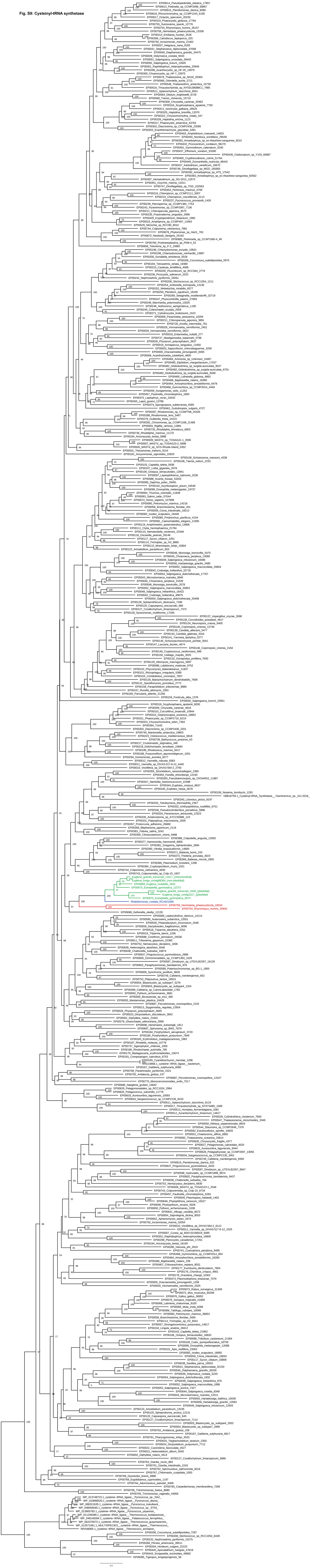

Fig. S10: Glutamyl-tRNA synthetase/glutaminyl-tRNA synthetase

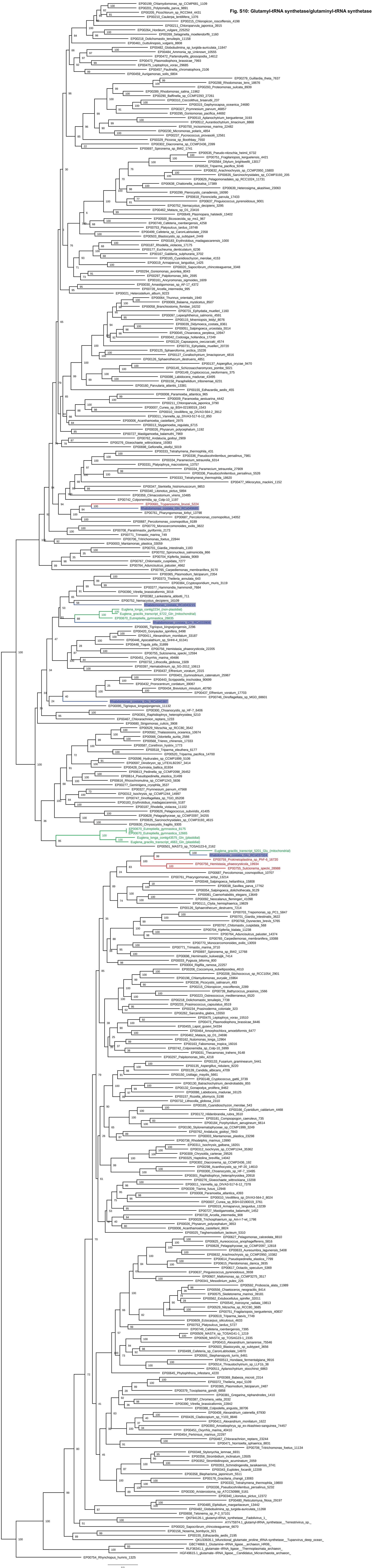

Fig. S11: Glycyl-tRNA synthetase

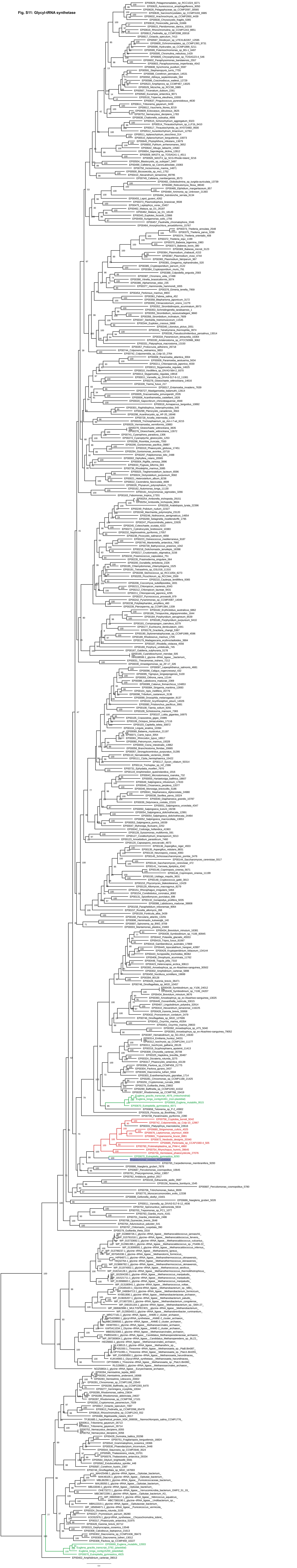

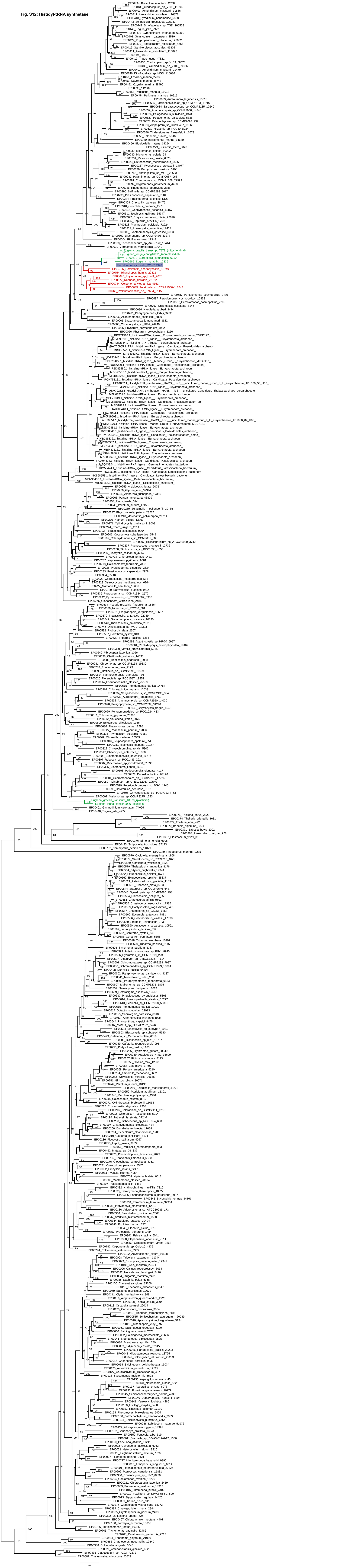

Fig. S12: Histidyl-tRNA synthetase

Fig. S13: Isoleucyl-tRNA synthetase

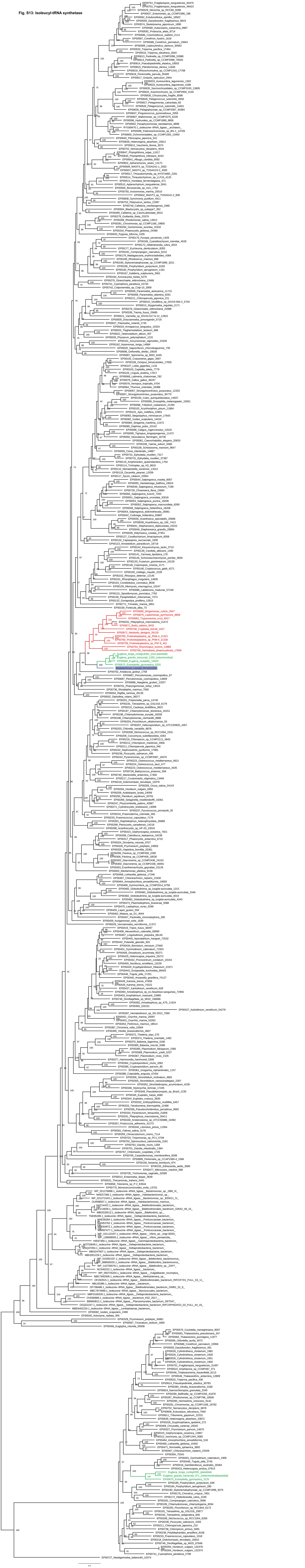

Fig. S14: Leucyl-tRNA synthetase

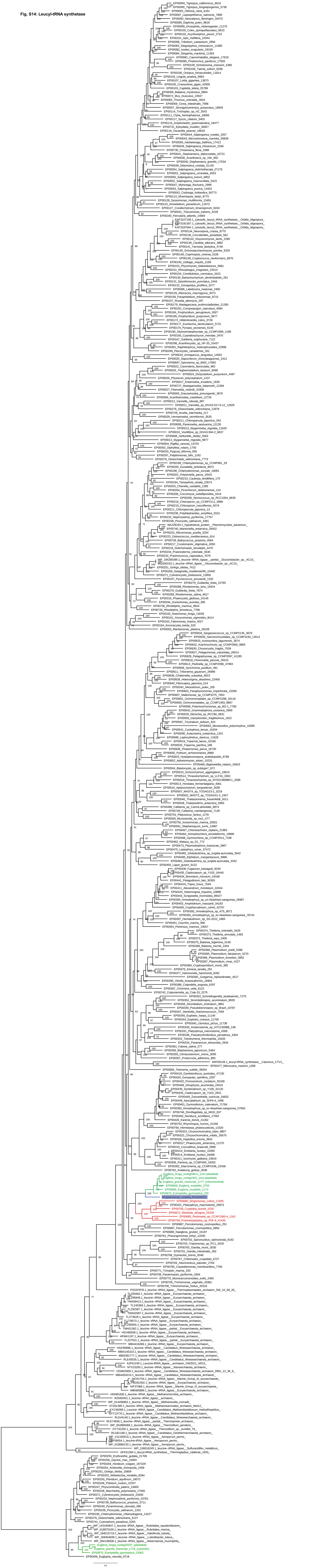

Fig. S15: Lysyl-tRNA synthetase

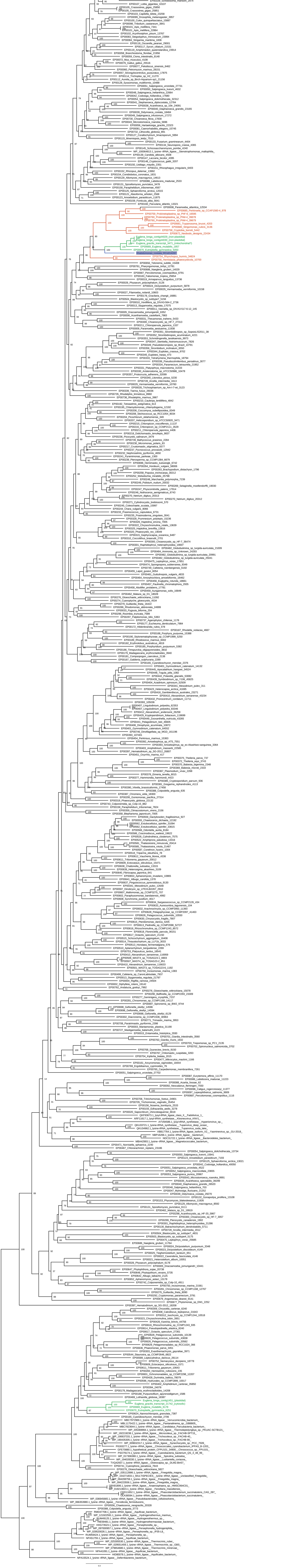

Fig. S16: Methionyl-tRNA synthetase

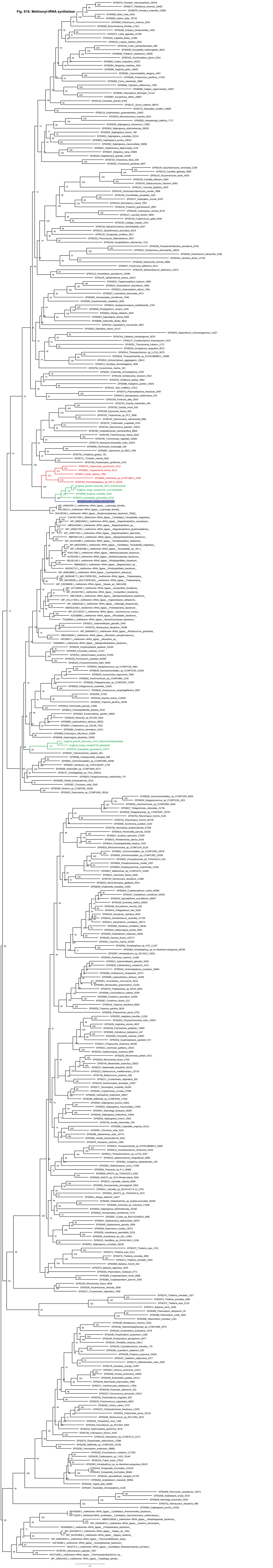

Fig. S17: Phenylalanyl-tRNA synthetase

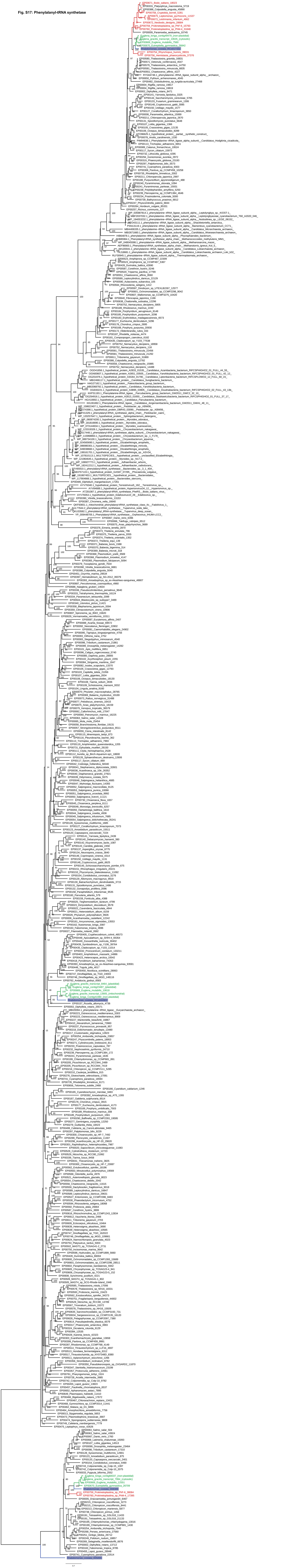

Fig. S18: Prolyl-tRNA synthetase

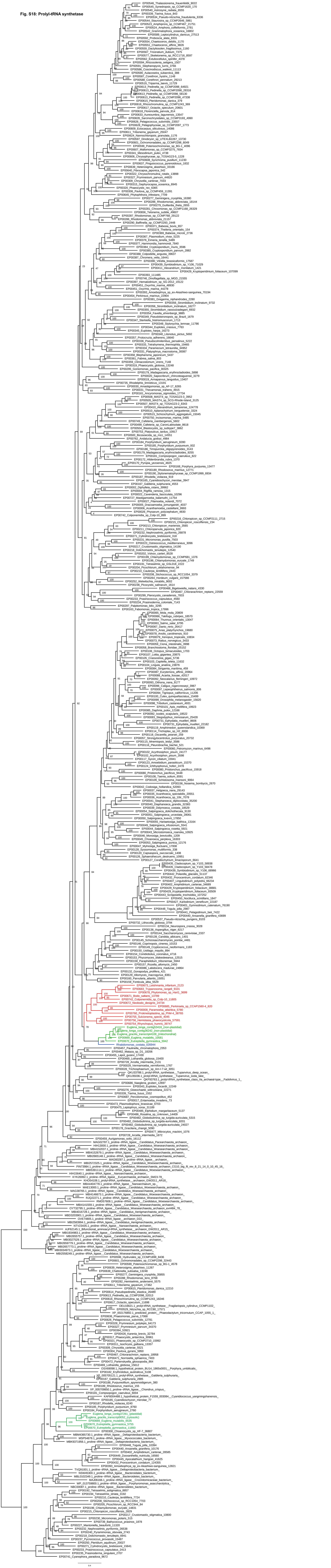

Fig. S19: Seryl-tRNA synthetase

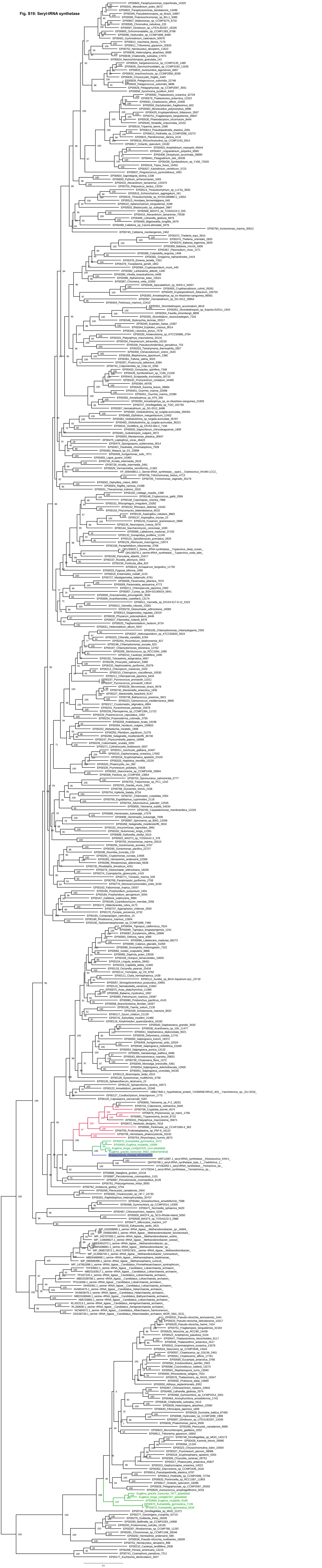

Fig. S20: Threonyl-tRNA synthetase

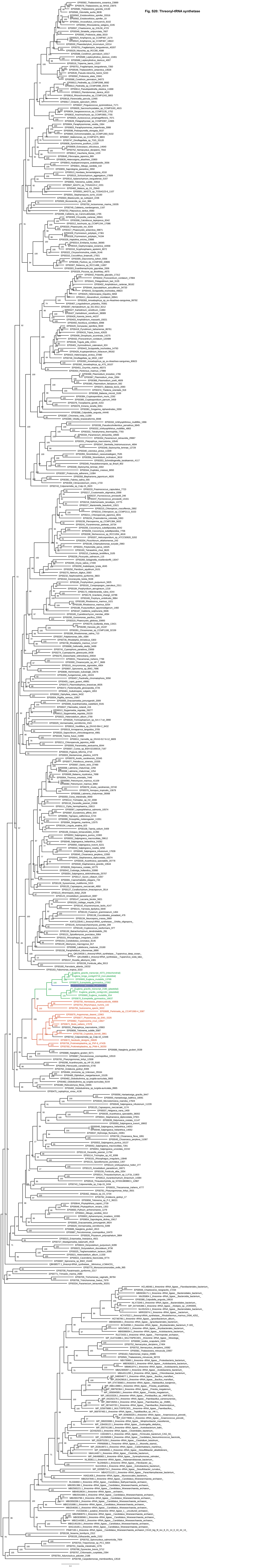

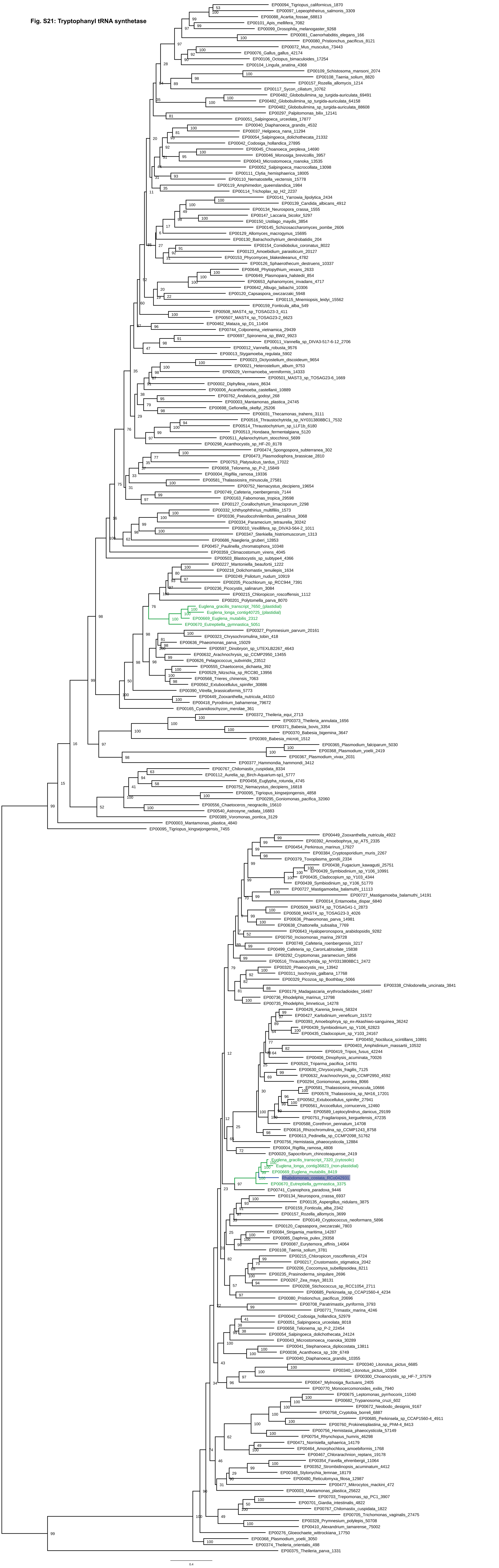

Fig. S21: Tryptophanyl tRNA synthetase

Fig. S22: Tyrosyl-tRNA synthetase

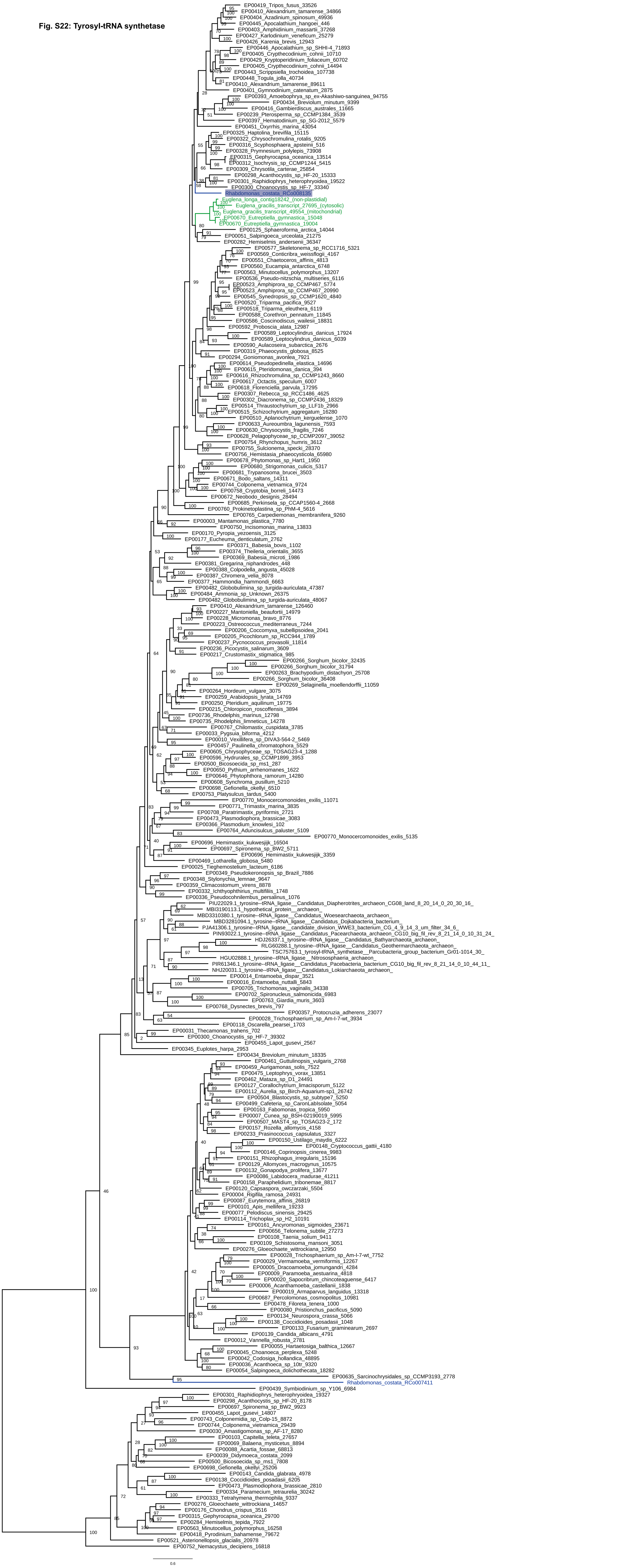

Fig. S23: Valyl-tRNA synthetase

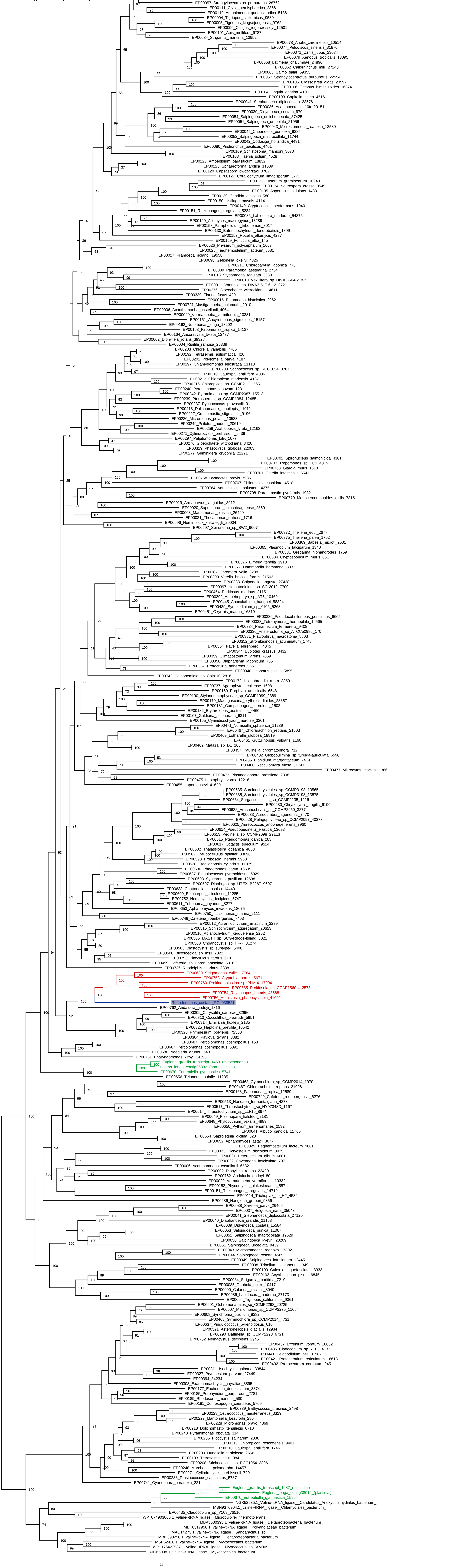

Supplement: Supplementary file 2 — Supplementary Figures S5-S23. [file 41598_2021_92174_MOESM2_ESM.pdf]
