# Supplementary material for: Heterotrophic euglenid Rhabdomonas costata resembles its phototrophic relatives in many aspects of molecular and cell biology
Source: Sci Rep. 2021 Jun 22;11:13070. doi: 10.1038/s41598-021-92174-3 (PMC8219788; doi:10.1038/s41598-021-92174-3)
Supplement: Supplementary file 4 — Supplementary Figures S34-S45. [file 41598_2021_92174_MOESM4_ESM.pdf]

Figs. S34-S45: Phylogenetic trees of enzymes involved in thiamine metabolism.

The tree of *R. costata* and homologues from NCBI and EukProt database (https://www.biorxiv.org/content/10.1101/2020.06.30.180687v1) was constructed in IQ-TREE using the model finder-selected model from a TrimAl-trimmed and manually inspected alignment. The values at the nodes represent ultrafast bootstraps from 1,000 repetitions. Euglenophyte sequences are shown in green, *Rhabdomonas costata* sequences in blue, other euglenozoa in red. The supplementary figures are referred in Supplementary table S10. Please note that the sequence ID numbers starting EP0… are arbitrary and do not correspond to the transcriptome database of respective species. The figure was created in FigTree v 1.4.4.

Fig. S34: Phosphomethylpyrimidine synthase [EC:4.1.99.17]
Fig. S35: Hydroxymethylpyrimidine/phosphomethylpyrimidine kinase [EC:2.7.1.49 /2.7.4.7]
Fig. S36: Cysteine-dependent adenosine diphosphate thiazole synthase [EC:2.4.2.60]
Fig. S37: Thiamine-phosphate pyrophosphorylase [EC:2.5.1.3]
Fig. S38: Acid phosphatase [EC:3.1.3.2]
Fig. S39: Thiamine phosphate phosphatase [EC 3.1.3.100]
Fig. S40: Nucleoside-triphosphatase [EC:3.6.1.15]
Fig. S41: Thiamine pyrophosphokinase [EC:2.7.6.2]
Fig. S42: Adenylate kinase [EC:2.7.4.3]
Fig. S43: Thiamine-triphosphatase [EC:3.6.1.28]
Fig. S44: Thiaminase (transcriptional activator TenA) [EC:3.5.99.2]
Fig. S45: TENA_E; formylaminopyrimidine deformylase /aminopyrimidine aminohydrolase [EC:3.5.1.- /3.5.99.-]

Fig. S34

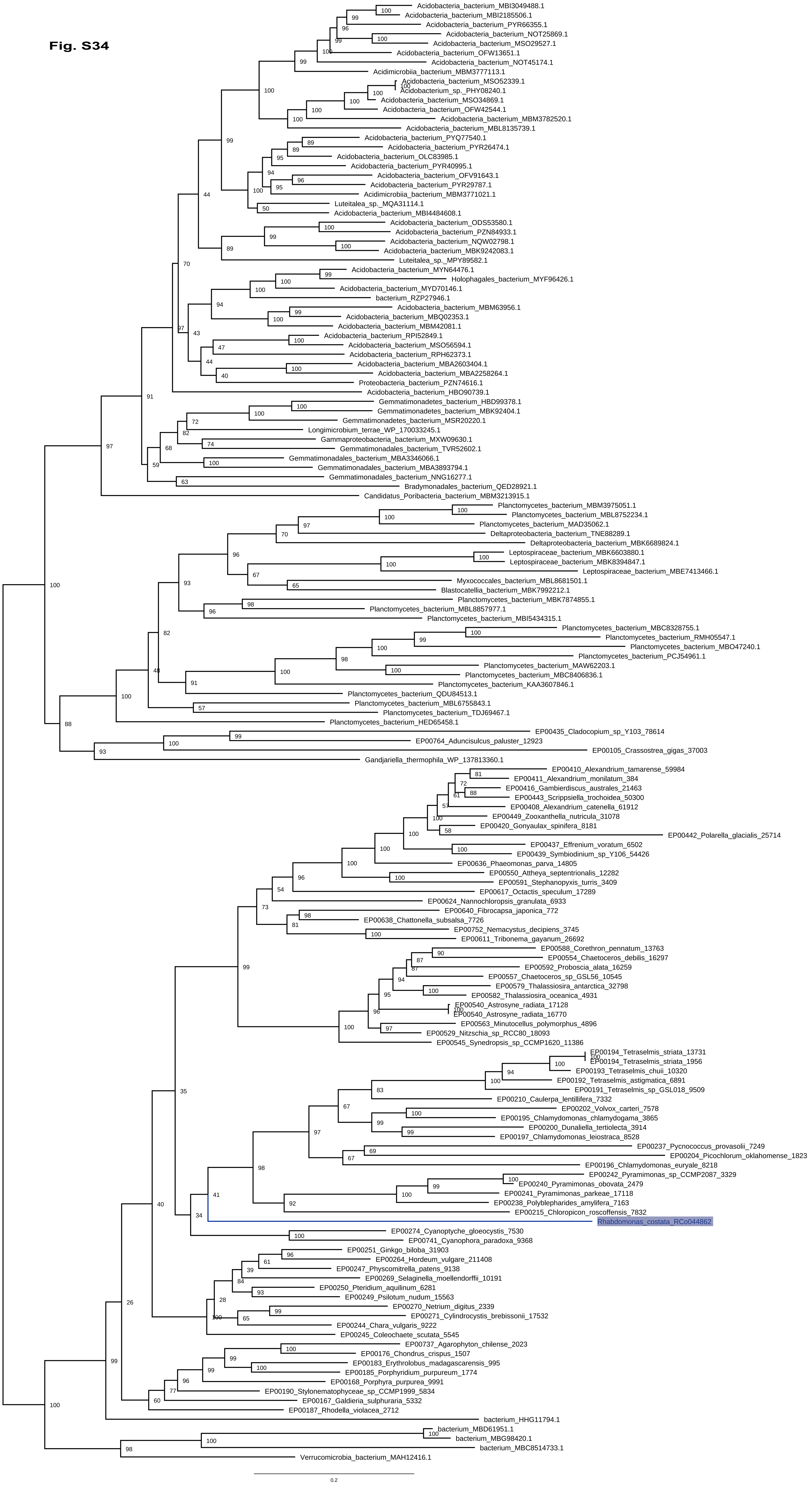
Acidobacteria_bacterium_MBI3049488.1
Acidobacteria_bacterium_MBI2185506.1
Acidobacteria_bacterium_PYR66355.1
Acidobacteria_bacterium_NOT25869.1
Acidobacteria_bacterium_MSO29527.1
Acidobacteria_bacterium_OFW13651.1
Acidobacteria_bacterium_NOT45174.1
Acidimicrobiia_bacterium_MBM3777113.1
Acidobacteria_bacterium_MSO52339.1
Acidobacterium_sp._PHY08240.1
Acidobacteria_bacterium_MSO34869.1
Acidobacteria_bacterium_OFW42544.1
Acidobacteria_bacterium_MBM3782520.1
Acidobacteria_bacterium_MBL8135739.1
Acidobacteria_bacterium_PYQ77540.1
Acidobacteria_bacterium_PYR26474.1
Acidobacteria_bacterium_OLC83985.1
Acidobacteria_bacterium_PYR40995.1
Acidobacteria_bacterium_OFV91643.1
Acidobacteria_bacterium_PYR29787.1
Acidimicrobiia_bacterium_MBM3771021.1
Luteitalea_sp._MQA31114.1
Acidobacteria_bacterium_MBI4484608.1
Acidobacteria_bacterium_ODS53580.1
Acidobacteria_bacterium_PZN84933.1
Acidobacteria_bacterium_NQW02798.1
Acidobacteria_bacterium_MBK9242083.1
Luteitalea_sp._MPY89582.1
Acidobacteria_bacterium_MYN64476.1
Holophagales_bacterium_MYF96426.1
Acidobacteria_bacterium_MYD70146.1
bacterium_RZP27946.1
Acidobacteria_bacterium_MBM63956.1
Acidobacteria_bacterium_MBQ02353.1
Acidobacteria_bacterium_MBM42081.1
Acidobacteria_bacterium_RPI52849.1
Acidobacteria_bacterium_MSO56594.1
Acidobacteria_bacterium_RPH62373.1
Acidobacteria_bacterium_MBA2603404.1
Acidobacteria_bacterium_MBA2258264.1
Proteobacteria_bacterium_PZN74616.1
Acidobacteria_bacterium_HBO90739.1
Gemmatimonadetes_bacterium_HBD99378.1
Gemmatimonadetes_bacterium_MBK92404.1
Gemmatimonadetes_bacterium_MSR20220.1
Longimicrobium_terrae_WP_170033245.1
Gammaproteobacteria_bacterium_MXW09630.1
Gemmatimonadales_bacterium_TVR52602.1
Gemmatimonadales_bacterium_MBA3346066.1
Gemmatimonadales_bacterium_MBA3893794.1
Gemmatimonadales_bacterium_NNG16277.1
Bradymonadales_bacterium_QED28921.1
Candidatus_Poribacteria_bacterium_MBM3213915.1
Planctomycetes_bacterium_MBM3975051.1
Planctomycetes_bacterium_MBL8752234.1
Planctomycetes_bacterium_MAD35062.1
Deltaproteobacteria_bacterium_TNE88289.1
Deltaproteobacteria_bacterium_MBK6689824.1
Leptospiraceae_bacterium_MBK6603880.1
Leptospiraceae_bacterium_MBK8394847.1
Leptospiraceae_bacterium_MBE7413466.1
Myxococcales_bacterium_MBL8681501.1
Blastocatellia_bacterium_MBK7992212.1
Planctomycetes_bacterium_MBK7874855.1
Planctomycetes_bacterium_MBL8857977.1
Planctomycetes_bacterium_MBI5434315.1
Planctomycetes_bacterium_MBC8328755.1
Planctomycetes_bacterium_RMH05547.1
Planctomycetes_bacterium_MBO47240.1
Planctomycetes_bacterium_PCJ54961.1
Planctomycetes_bacterium_MAW62203.1
Planctomycetes_bacterium_MBC8406836.1
Planctomycetes_bacterium_KAA3607846.1
Planctomycetes_bacterium_QDU84513.1
Planctomycetes_bacterium_MBL6755843.1
Planctomycetes_bacterium_TDJ69467.1
Planctomycetes_bacterium_HED65458.1
EP00435_Cladocopium_sp_Y103_78614
EP00764_Aduncisulcus_paluster_12923
EP00105_Crassostrea_gigas_37003
Gandjariella_thermophila_WP_137813360.1
EP00410_Alexandrium_tamarense_59984
EP00411_Alexandrium_monilatum_384
EP00416_Gambierdiscus_australes_21463
EP00443_Scrippsiella_trochoidea_50300
EP00408_Alexandrium_catenella_61912
EP00449_Zooxanthella_nutricula_31078
EP00420_Gonyaulax_spinifera_8181
EP00442_Polarella_glacialis_25714
EP00437_Effrenium_voratum_6502
EP00439_Symbiodinium_sp_Y106_54426
EP00636_Phaeomonas_parva_14805
EP00550_Attheya_septentrionalis_12282
EP00591_Stephanopyxis_turris_3409
EP00617_Octactis_speculum_17289
EP00624_Nannochloropsis_granulata_6933
EP00640_Fibrocapsa_japonica_772
EP00638_Chattonella_subsalsa_7726
EP00752_Nemacystus_decipiens_3745
EP00611_Tribonema_gayanum_26692
EP00588_Corethron_pennatum_13763
EP00554_Chaetoceros_debilis_16297
EP00592_Proboscia_alata_16259
EP00557_Chaetoceros_sp_GSL56_10545
EP00579_Thalassiosira_antarctica_32798
EP00582_Thalassiosira_oceanica_4931
EP00540_Astrosyne_radiata_17128
EP00540_Astrosyne_radiata_16770
EP00563_Minutocellus_polymorphus_4896
EP00529_Nitzschia_sp_RCC80_18093
EP00545_Synedropsis_sp_CCMP1620_11386
EP00194_Tetraselmis_striata_13731
EP00194_Tetraselmis_striata_1956
EP00193_Tetraselmis_chuii_10320
EP00192_Tetraselmis_astigmatica_6891
EP00191_Tetraselmis_sp_GSL018_9509
EP00210_Caulerpa_lentillifera_7332
EP00202_Volvox_carteri_7578
EP00195_Chlamydomonas_chlamydogama_3865
EP00200_Dunaliella_tertiolecta_3914
EP00197_Chlamydomonas_leiostraca_8528
EP00237_Pycnococcus_provasolii_7249
EP00204_Picochlorum_oklahomense_1823
EP00196_Chlamydomonas_euryale_8218
EP00242_Pyramimonas_sp_CCMP2087_3329
EP00240_Pyramimonas_obovata_2479
EP00241_Pyramimonas_parkeae_17118
EP00238_Polyblepharides_amylifera_7163
EP00215_Chloropicon_roscoffensis_7832
Rhabdomonas_costata_RCo044862
EP00274_Cyanoptyche_gloeocystis_7530
EP00741_Cyanophora_paradoxa_9368
EP00251_Ginkgo_biloba_31903
EP00264_Hordeum_vulgare_211408
EP00247_Physcomitrella_patens_9138
EP00269_Selaginella_moellendorffii_10191
EP00250_Pteridium_aquilinum_6281
EP00249_Psilotum_nudum_15563
EP00270_Netrium_digitus_2339
EP00271_Cylindrocystis_brebissonii_17532
EP00244_Chara_vulgaris_9222
EP00245_Coleochaete_scutata_5545
EP00737_Agarophyton_chilense_2023
EP00176_Chondrus_crispus_1507
EP00183_Erythrolobus_madagascarensis_995
EP00185_Porphyridium_purpureum_1774
EP00168_Porphyra_purpurea_9991
EP00190_Stylonematophyceae_sp_CCMP1999_5834
EP00167_Galdieria_sulphuraria_5332
EP00187_Rhodella_violacea_2712
bacterium_HHG11794.1
bacterium_MBD61951.1
bacterium_MBG98420.1
bacterium_MBC8514733.1
Verrucomicrobia_bacterium_MAH12416.1
0.2

Fig. S35

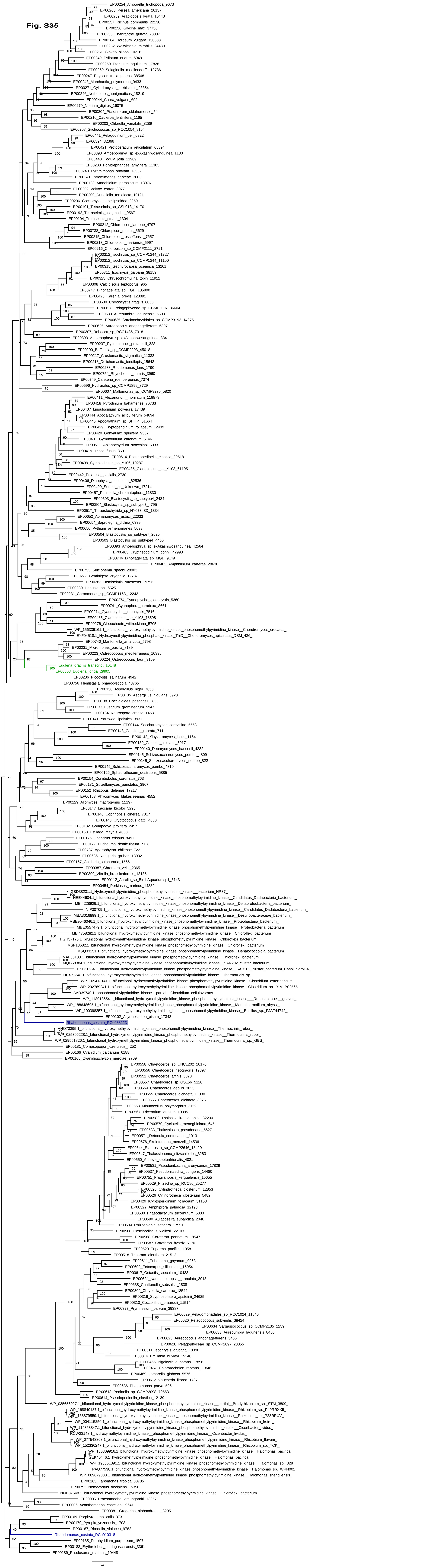

EP00254_Amborella_trichopoda_9673
EP00268_Persea_americana_26137
EP00259_Arabidopsis_lyrata_16443
EP00257_Ricinus_communis_22138
EP00256_Glycine_max_37736
EP00255_Erythranthe_guttata_23007
EP00264_Hordeum_vulgare_150588
EP00252_Welwitschia_mirabilis_24480
EP00251_Ginkgo_biloba_10216
EP00249_Psilotum_nudum_6949
EP00250_Pteridium_aquilinum_17828
EP00269_Selaginella_moellendorffii_12786
EP00247_Physcomitrella_patens_38568
EP00248_Marchantia_polymorpha_9433
EP00271_Cylindrocystis_brebissonii_23354
EP00246_Nothoceros_aenigmaticus_18219
EP00244_Chara_vulgaris_692
EP00270_Netrium_digitus_16075
EP00204_Picochlorum_oklahomense_54
EP00210_Caulerpa_lentillifera_1165
EP00203_Chlorella_variabilis_3289
EP00208_Stichococcus_sp_RCC1054_8164
EP00441_Pelagodinium_beii_6322
EP00394_32366
EP00421_Protoceratium_reticulatum_65394
EP00393_Amoebophrya_sp_exAkashiwosanguinea_1130
EP00448_Togula_jolla_11989
EP00238_Polyblepharides_amylifera_11383
EP00240_Pyramimonas_obovata_13552
EP00241_Pyramimonas_parkeae_3663
EP00123_Amoebidium_parasiticum_18976
EP00202_Volvox_carteri_3077
EP00200_Dunaliella_tertiolecta_10121
EP00206_Coccomyxa_subellipsoidea_2250
EP00191_Tetraselmis_sp_GSL018_14170
EP00192_Tetraselmis_astigmatica_9567
EP00194_Tetraselmis_striata_13041
EP00212_Chloropicon_laureae_4797
EP00738_Chloropicon_primus_5629
EP00215_Chloropicon_roscoffensis_7657
EP00213_Chloropicon_mariensis_5997
EP00216_Chloropicon_sp_CCMP2111_2721
EP00312_Isochrysis_sp_CCMP1244_31727
EP00312_Isochrysis_sp_CCMP1244_11150
EP00315_Gephyrocapsa_oceanica_13261
EP00311_Isochrysis_galbana_38159
EP00323_Chrysochromulina_tobin_11912
EP00308_Calcidiscus_leptoporus_965
EP00747_Dinoflagellata_sp_TGD_185890
EP00426_Karenia_brevis_120091
EP00630_Chrysocystis_fragilis_8033
EP00628_Pelagophyceae_sp_CCMP2097_36604
EP00633_Aureoumbra_lagunensis_6503
EP00635_Sarcinochrysidales_sp_CCMP3193_14275
EP00625_Aureococcus_anophagefferens_6807
EP00307_Rebecca_sp_RCC1486_7318
EP00393_Amoebophrya_sp_exAkashiwosanguinea_834
EP00237_Pycnococcus_provasolii_328
EP00290_Baffinella_sp_CCMP2293_45018
EP00217_Crustomastix_stigmatica_11332
EP00218_Dolichomastix_tenuilepis_15643
EP00288_Rhodomonas_lens_1790
EP00754_Rhynchopus_humris_3960
EP00749_Cafeteria_roenbergensis_7374
EP00596_Hydrurales_sp_CCMP1899_3729
EP00607_Mallomonas_sp_CCMP3275_5820
EP00411_Alexandrium_monilatum_119873
EP00418_Pyrodinium_bahamense_76733
EP00407_Lingulodinium_polyedra_17439
EP00444_Apocalathium_aciculiferum_54694
EP00446_Apocalathium_sp_SHHI4_51664
EP00429_Kryptoperidinium_foliaceum_12439
EP00420_Gonyaulax_spinifera_9557
EP00401_Gymnodinium_catenatum_5146
EP00511_Aplanochytrium_stocchinoi_6033
EP00419_Tripos_fusus_85011
EP00614_Pseudopedinella_elastica_29518
EP00439_Symbiodinium_sp_Y106_10287
EP00435_Cladocopium_sp_Y103_61195
EP00442_Polarella_glacialis_2730
EP00406_Dinophysis_acuminata_82536
EP00490_Sorites_sp_Unknown_17214
EP00457_Paulinella_chromatophora_11830
EP00503_Blastocystis_sp_subtype4_2484
EP00504_Blastocystis_sp_subtype7_4795
EP00517_Thraustochytrida_sp_NY07348D_1334
EP00652_Aphanomyces_astaci_22033
EP00654_Saprolegnia_diclina_6339
EP00650_Pythium_arrhenomanes_5093
EP00504_Blastocystis_sp_subtype7_2625
EP00503_Blastocystis_sp_subtype4_4466
EP00393_Amoebophrya_sp_exAkashiwosanguinea_42564
EP00405_Crypthecodinium_cohnii_42993
EP00746_Dinoflagellata_sp_MGD_9149
EP00402_Amphidinium_carterae_28630
EP00755_Sulcionema_specki_28903
EP00277_Geminigera_cryophila_12737
EP00283_Hemiselmis_rufescens_19756
EP00280_Hanusia_phi_6525
EP00281_Chroomonas_sp_CCMP1168_12243
EP00274_Cyanoptyche_gloeocystis_5360
EP00741_Cyanophora_paradoxa_8661
EP00274_Cyanoptyche_gloeocystis_7516
EP00435_Cladocopium_sp_Y103_78598
EP00276_Gloeochaete_wittrockiana_5705
WP_156339161.1_bifunctional_hydroxymethylpyrimidine_kinase_phosphomethylpyrimidine_kinase__Chondromyces_crocatus_
EYF04518.1_Hydroxymethylpyrimidine_phosphate_kinase_ThiD__Chondromyces_apiculatus_DSM_436_
EP00740_Mantoniella_antarctica_5798
EP00231_Micromonas_pusilla_8189
EP00223_Ostreococcus_mediterraneus_10396
EP00224_Ostreococcus_tauri_3159
Euglena_gracilis_transcript_16148
EP00668_Euglena_longa_29905
EP00236_Picocystis_salinarum_4942
EP00756_Hemistasia_phaeocysticola_43765
EP00136_Aspergillus_niger_7833
EP00135_Aspergillus_nidulans_5928
EP00138_Coccidioides_posadasii_2833
EP00133_Fusarium_graminearum_5947
EP00134_Neurospora_crassa_1463
EP00141_Yarrowia_lipolytica_3931
EP00144_Saccharomyces_cerevisiae_5553
EP00143_Candida_glabrata_711
EP00142_Kluyveromyces_lactis_1164
EP00139_Candida_albicans_5017
EP00140_Debaryomyces_hansenii_4232
EP00145_Schizosaccharomyces_pombe_4809
EP00145_Schizosaccharomyces_pombe_822
EP00145_Schizosaccharomyces_pombe_4810
EP00126_Sphaerothecum_destruens_5885
EP00154_Conidiobolus_coronatus_763
EP00131_Spizellomyces_punctatus_3907
EP00152_Rhizopus_delemar_17217
EP00153_Phycomyces_blakesleeanus_4552
EP00129_Allomyces_macrogynus_11197
EP00147_Laccaria_bicolor_5298
EP00146_Coprinopsis_cinerea_7817
EP00148_Cryptococcus_gattii_4850
EP00132_Gonapodya_prolifera_2457
EP00150_Ustilago_maydis_4053
EP00176_Chondrus_crispus_8491
EP00177_Eucheuma_denticulatum_7128
EP00737_Agarophyton_chilense_722
EP00686_Naegleria_gruberi_13032
EP00167_Galdieria_sulphuraria_1566
EP00387_Chromera_velia_2365
EP00390_Vitrella_brassicaformis_13135
EP00112_Aurelia_sp_BirchAquariumsp1_5143
EP00454_Perkinsus_marinus_14882
GBD38231.1_Hydroxymethylpyrimidine_phosphomethylpyrimidine_kinase__bacterium_HR37_
HEE44604.1_bifunctional_hydroxymethylpyrimidine_kinase_phosphomethylpyrimidine_kinase__Candidatus_Dadabacteria_bacterium_
MBI4228929.1_bifunctional_hydroxymethylpyrimidine_kinase_phosphomethylpyrimidine_kinase__Deltaproteobacteria_bacterium_
NIP30709.1_bifunctional_hydroxymethylpyrimidine_kinase_phosphomethylpyrimidine_kinase__Candidatus_Dadabacteria_bacterium_
MBA3016899.1_bifunctional_hydroxymethylpyrimidine_kinase_phosphomethylpyrimidine_kinase__Desulfobacteraceae_bacterium_
MBE9546046.1_bifunctional_hydroxymethylpyrimidine_kinase_phosphomethylpyrimidine_kinase__Proteobacteria_bacterium_
MBE0557479.1_bifunctional_hydroxymethylpyrimidine_kinase_phosphomethylpyrimidine_kinase__Proteobacteria_bacterium_
MBI4758282.1_bifunctional_hydroxymethylpyrimidine_kinase_phosphomethylpyrimidine_kinase__Chloroflexi_bacterium_
HGH57175.1_bifunctional_hydroxymethylpyrimidine_kinase_phosphomethylpyrimidine_kinase__Chloroflexi_bacterium_
MSP13682.1_bifunctional_hydroxymethylpyrimidine_kinase_phosphomethylpyrimidine_kinase__Chloroflexi_bacterium_
MSQ33151.1_bifunctional_hydroxymethylpyrimidine_kinase_phosphomethylpyrimidine_kinase__Dehalococcoidia_bacterium_
MAF53188.1_bifunctional_hydroxymethylpyrimidine_kinase_phosphomethylpyrimidine_kinase__Chloroflexi_bacterium_
MQG68384.1_bifunctional_hydroxymethylpyrimidine_kinase_phosphomethylpyrimidine_kinase__SAR202_cluster_bacterium_
PKB61654.1_bifunctional_hydroxymethylpyrimidine_kinase_phosphomethylpyrimidine_kinase__SAR202_cluster_bacterium_CaspChloroG4_
HEX71348.1_bifunctional_hydroxymethylpyrimidine_kinase_phosphomethylpyrimidine_kinase__Thermorudis_sp._
WP_165413141.1_bifunctional_hydroxymethylpyrimidine_kinase_phosphomethylpyrimidine_kinase__Clostridium_estertheticum_
WP_202769241.1_bifunctional_hydroxymethylpyrimidine_kinase_phosphomethylpyrimidine_kinase__Clostridium_sp._YIM_B02565_
AAD39740.1_phosphomethylpyrimidine_kinase__partial__Clostridium_cellulovorans_
WP_118013654.1_bifunctional_hydroxymethylpyrimidine_kinase_phosphomethylpyrimidine_kinase__Ruminococcus__gnavus_
WP_188648695.1_bifunctional_hydroxymethylpyrimidine_kinase_phosphomethylpyrimidine_kinase__Marinithermofilum_abyssi_
WP_100398357.1_bifunctional_hydroxymethylpyrimidine_kinase_phosphomethylpyrimidine_kinase__Bacillus_sp._FJAT44742_
EP00102_Acyrthosiphon_pisum_17343
Rhabdomonas_costata_RCo0038223
HHO73395.1_bifunctional_hydroxymethylpyrimidine_kinase_phosphomethylpyrimidine_kinase__Thermocrinis_ruber_
WP_025306228.1_bifunctional_hydroxymethylpyrimidine_kinase_phosphomethylpyrimidine_kinase__Thermocrinis_ruber_
WP_029551826.1_bifunctional_hydroxymethylpyrimidine_kinase_phosphomethylpyrimidine_kinase__Thermocrinis_sp._GBS_
EP00181_Compsopogon_caeruleus_4252
EP00166_Cyanidium_caldarium_6188
EP00165_Cyanidioschyzon_merolae_2769
EP00558_Chaetoceros_sp_UNC1202_10170
EP00556_Chaetoceros_neogracilis_19397
EP00551_Chaetoceros_affinis_5873
EP00557_Chaetoceros_sp_GSL56_5120
EP00554_Chaetoceros_debilis_3023
EP00555_Chaetoceros_dichaeta_11330
EP00555_Chaetoceros_dichaeta_8875
EP00563_Minutocellus_polymorphus_3159
EP00567_Triceratium_dubium_10395
EP00582_Thalassiosira_oceanica_32200
EP00570_Cyclotella_meneghiniana_645
EP00583_Thalassiosira_pseudonana_5627
EP00581_Detonula_confervacea_10131
EP00576_Skeletonema_menzelii_14536
EP00544_Staurosira_sp_CCMP2646_13420
EP00547_Thalassionema_nitzschioides_3283
EP00550_Attheya_septentrionalis_4021
EP00531_Pseudonitzschia_arenysensis_17829
EP00537_Pseudonitzschia_pungens_14480
EP00751_Fragilariopsis_kerguelensis_15655
EP00529_Nitzschia_sp_RCC80_25277
EP00526_Cylindrotheca_closterium_12853
EP00526_Cylindrotheca_closterium_5482
EP00429_Kryptoperidinium_foliaceum_31168
EP00522_Amphiprora_paludosa_12193
EP00530_Phaeodactylum_tricornutum_5383
EP00590_Aulacoseira_subarctica_2346
EP00594_Rhizosolenia_setigera_17951
EP00586_Coscinodiscus_wailesii_22103
EP00588_Corethron_pennatum_18547
EP00587_Corethron_hystrix_5170
EP00520_Triparma_pacifica_1058
EP00518_Triparma_eleuthera_21512
EP00611_Tribonema_gayanum_9968
EP00609_Ectocarpus_siliculosus_16054
EP00617_Octactis_speculum_10433
EP00624_Nannochloropsis_granulata_3913
EP00638_Chattonella_subsalsa_1838
EP00309_Chrysotila_carterae_18542
EP00316_Scyphosphaera_apsteinii_24625
EP00310_Coccolithus_braarudii_11514
EP00327_Prymnesium_parvum_39387
EP00626_Pelagomonadales_sp_RCC1024_11846
EP00629_Pelagococcus_subviridis_39424
EP00634_Sargassococcus_sp_CCMP2135_1259
EP00633_Aureoumbra_lagunensis_8450
EP00625_Aureococcus_anophagefferens_5456
EP00628_Pelagophyceae_sp_CCMP2097_28355
EP00311_Isochrysis_galbana_18396
EP00314_Emiliania_huxleyi_15140
EP00466_Bigelowiella_natans_17856
EP00467_Chlorarachnion_reptans_11846
EP00469_Lotharella_globosa_5576
EP00612_Vaucheria_litorea_1787
EP00636_Phaeomonas_parva_596
EP00613_Pedinella_sp_CCMP2098_70553
EP00614_Pseudopedinella_elastica_12139
WP_035656927.1_bifunctional_hydroxymethylpyrimidine_kinase_phosphomethylpyrimidine_kinase__partial__Bradyrhizobium_sp._STM_3809_
WP_168840187.1_bifunctional_hydroxymethylpyrimidine_kinase_phosphomethylpyrimidine_kinase__Rhizobium_sp._P40RRXXII_
WP_168879559.1_bifunctional_hydroxymethylpyrimidine_kinase_phosphomethylpyrimidine_kinase__Rhizobium_sp._P28RRXV_
WP_204115250.1_bifunctional_hydroxymethylpyrimidine_kinase_phosphomethylpyrimidine_kinase__Rhizobium_freirei_
WP_114363847.1_bifunctional_hydroxymethylpyrimidine_kinase_phosphomethylpyrimidine_kinase__Ciceribacter_lividus_
RCW23148.1_hydroxymethylpyrimidine_kinase_phosphomethylpyrimidine_kinase__Ciceribacter_lividus_
WP_077548008.1_bifunctional_hydroxymethylpyrimidine_kinase_phosphomethylpyrimidine_kinase__Rhizobium_flavum_
WP_152336247.1_bifunctional_hydroxymethylpyrimidine_kinase_phosphomethylpyrimidine_kinase__Rhizobium_sp._TCK_
WP_186809916.1_bifunctional_hydroxymethylpyrimidine_kinase_phosphomethylpyrimidine_kinase__Halomonas_pacifica_
GGK46446.1_hydroxymethylpyrimidine_phosphomethylpyrimidine_kinase__Halomonas_pacifica_
WP_195861391.1_bifunctional_hydroxymethylpyrimidine_kinase_phosphomethylpyrimidine_kinase__Halomonas_sp._328_
PAU77538.1_bifunctional_hydroxymethylpyrimidine_kinase_phosphomethylpyrimidine_kinase__Halomonas_sp._WRN001_
WP_089679080.1_bifunctional_hydroxymethylpyrimidine_kinase_phosphomethylpyrimidine_kinase__Halomonas_shengliensis_
EP00163_Fabomonas_tropica_33785
EP00752_Nemacystus_decipiens_15358
NMB87548.1_bifunctional_hydroxymethylpyrimidine_kinase_phosphomethylpyrimidine_kinase__Chloroflexi_bacterium_
EP00005_Dracoamoeba_jomungandrii_13257
EP00006_Acanthamoeba_castellanii_9641
EP00381_Gregarina_niphandrodes_3205
EP00169_Porphyra_umbilicalis_373
EP00170_Pyropia_yezoensis_1703
EP00187_Rhodella_violacea_9782
Rhabdomonas_costata_RCo010318
EP00185_Porphyridium_purpureum_1507
EP00183_Erythrolobus_madagascarensis_3361
EP00189_Rhodosorus_marinus_10448
0.3

Fig. S36

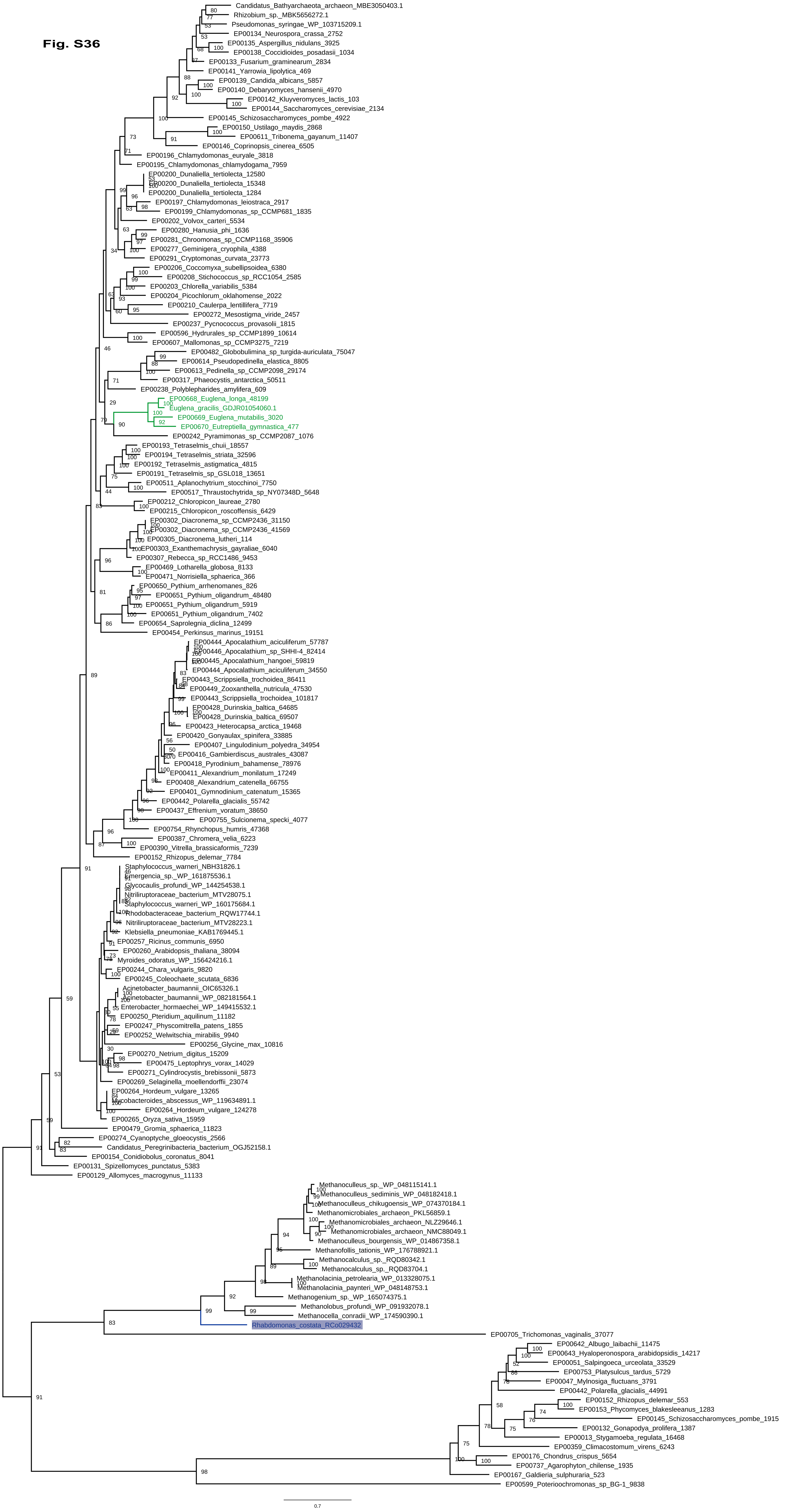

Fig. S37

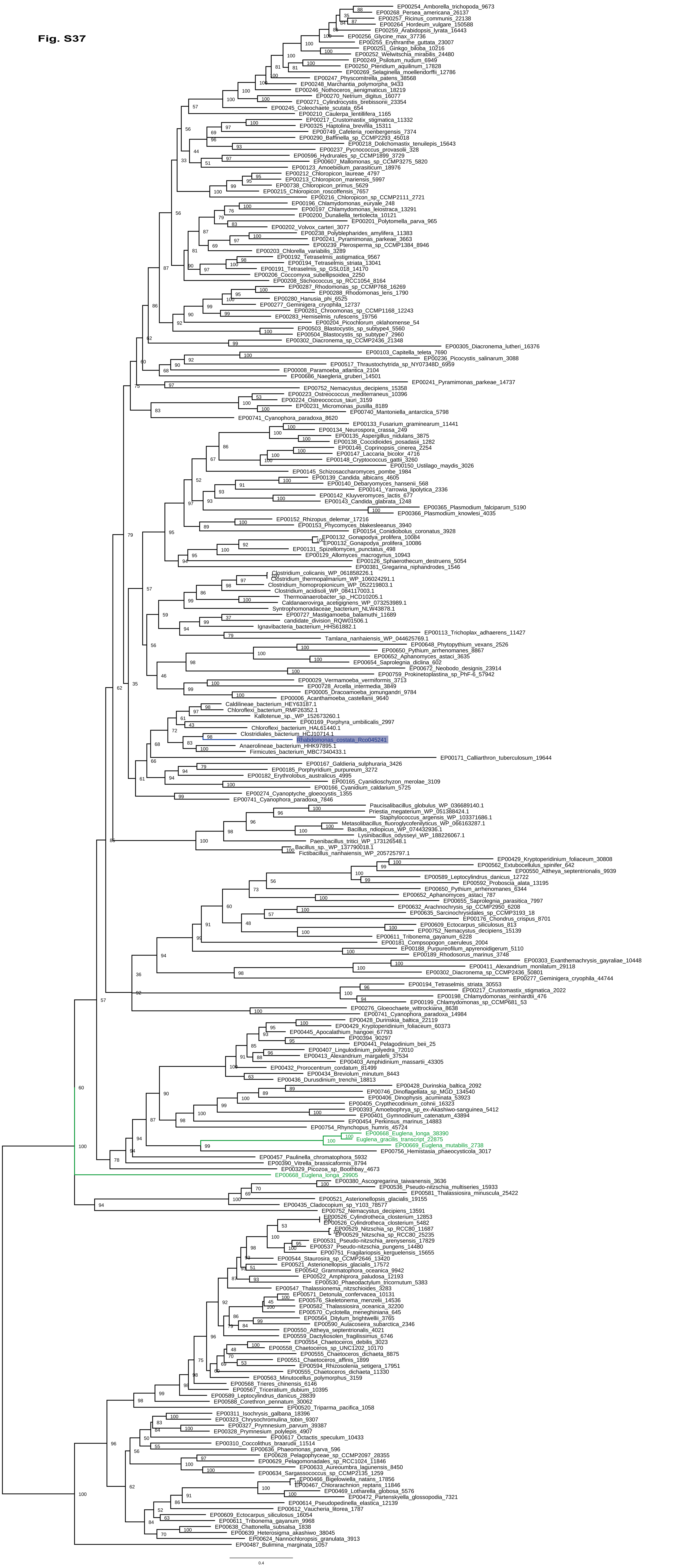

Fig. S38

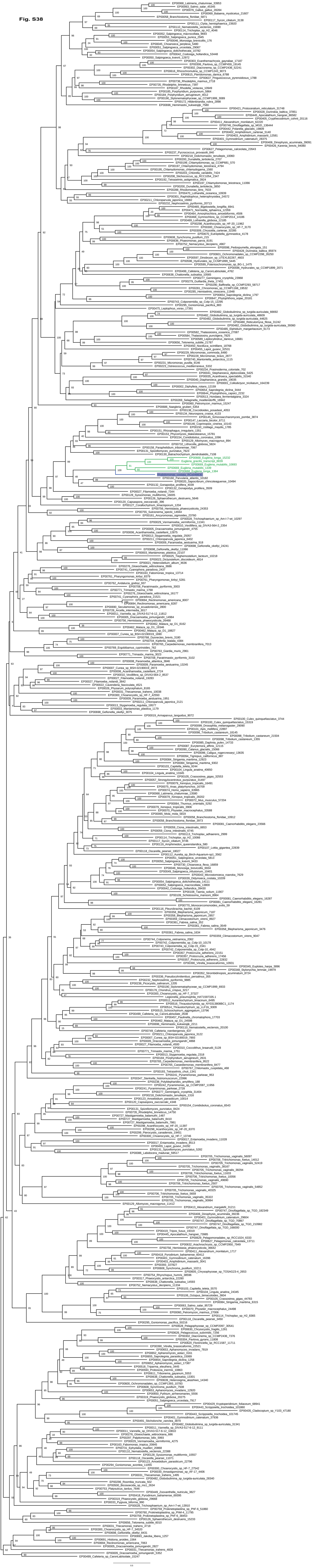

Fig. S39

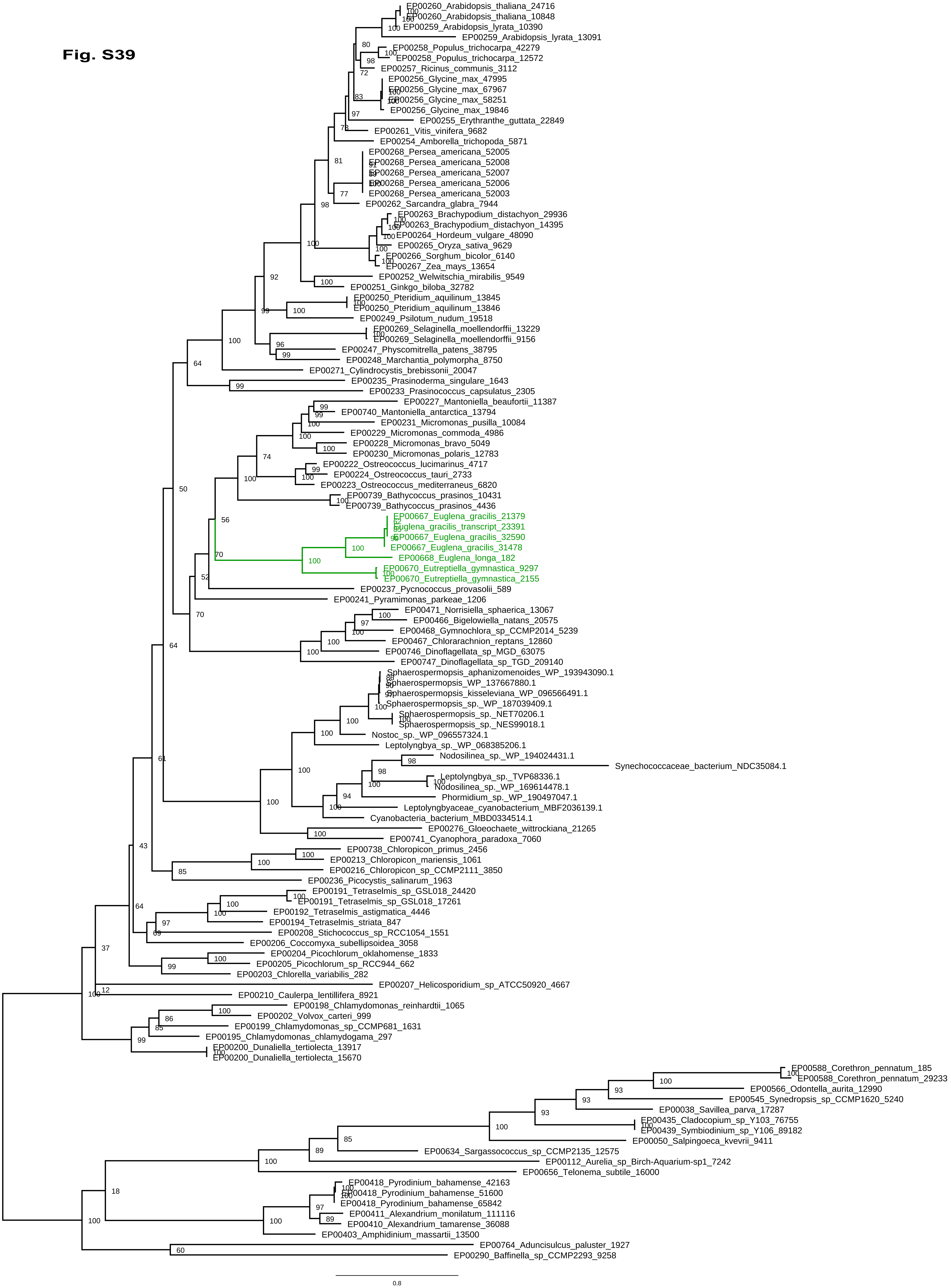
EP00260_Arabidopsis_thaliana_24716
EP00260_Arabidopsis_thaliana_10848
EP00259_Arabidopsis_lyrata_10390
EP00259_Arabidopsis_lyrata_13091
EP00258_Populus_trichocarpa_42279
EP00258_Populus_trichocarpa_12572
EP00257_Ricinus_communis_3112
EP00256_Glycine_max_47995
EP00256_Glycine_max_67967
EP00256_Glycine_max_58251
EP00256_Glycine_max_19846
EP00255_Erythranthe_guttata_22849
EP00261_Vitis_vinifera_9682
EP00254_Amborella_trichopoda_5871
EP00268_Persea_americana_52005
EP00268_Persea_americana_52008
EP00268_Persea_americana_52007
EP00268_Persea_americana_52006
EP00268_Persea_americana_52003
EP00262_Sarcandra_glabra_7944
EP00263_Brachypodium_distachyon_29936
EP00263_Brachypodium_distachyon_14395
EP00264_Hordeum_vulgare_48090
EP00265_Oryza_sativa_9629
EP00266_Sorghum_bicolor_6140
EP00267_Zea_mays_13654
EP00252_Welwitschia_mirabilis_9549
EP00251_Ginkgo_biloba_32782
EP00250_Pteridium_aquilinum_13845
EP00250_Pteridium_aquilinum_13846
EP00249_Psilotum_nudum_19518
EP00269_Selaginella_moellendorffii_13229
EP00269_Selaginella_moellendorffii_9156
EP00247_Physcomitrella_patens_38795
EP00248_Marchantia_polymorpha_8750
EP00271_Cylindrocystis_brebissonii_20047
EP00235_Prasinoderma_singulare_1643
EP00233_Prasinococcus_capsulatus_2305
EP00227_Mantoniella_beaufortii_11387
EP00740_Mantoniella_antarctica_13794
EP00231_Micromonas_pusilla_10084
EP00229_Micromonas_commoda_4986
EP00228_Micromonas_bravo_5049
EP00230_Micromonas_polaris_12783
EP00222_Ostreococcus_lucimarinus_4717
EP00224_Ostreococcus_tauri_2733
EP00223_Ostreococcus_mediterraneus_6820
EP00739_Bathycoccus_prasinos_10431
EP00739_Bathycoccus_prasinos_4436
EP00667_Euglena_gracilis_21379
Euglena_gracilis_transcript_23391
EP00667_Euglena_gracilis_32590
EP00667_Euglena_gracilis_31478
EP00668_Euglena_longa_182
EP00670_Eutreptiella_gymnastica_9297
EP00670_Eutreptiella_gymnastica_2155
EP00237_Pycnococcus_provasolii_589
EP00241_Pyramimonas_parkeae_1206
EP00471_Norrisiella_sphaerica_13067
EP00466_Bigelowiella_natans_20575
EP00468_Gymnochlora_sp_CCMP2014_5239
EP00467_Chlorarachnion_reptans_12860
EP00746_Dinoflagellata_sp_MGD_63075
EP00747_Dinoflagellata_sp_TGD_209140
Sphaerospermopsis_aphanizomenoides_WP_193943090.1
Sphaerospermopsis_WP_137667880.1
Sphaerospermopsis_kisseleviana_WP_096566491.1
Sphaerospermopsis_sp._WP_187039409.1
Sphaerospermopsis_sp._NET70206.1
Sphaerospermopsis_sp._NES99018.1
Nostoc_sp._WP_096557324.1
Leptolyngbya_sp._WP_068385246.1
Nodosilinea_sp._WP_194024431.1
Synechococcaceae_bacterium_NDC35084.1
Leptolyngbya_sp._TVP68336.1
Nodosilinea_sp._WP_169614478.1
Phormidium_sp._WP_190497047.1
Leptolyngbyaceae_cyanobacterium_MBF2036139.1
Cyanobacteria_bacterium_MBD0334514.1
EP00276_Gloeochaete_wittrockiana_21265
EP00741_Cyanophora_paradoxa_7060
EP00738_Chloropicon_primus_2456
EP00213_Chloropicon_mariensis_1061
EP00216_Chloropicon_sp_CCMP2111_3850
EP00236_Picocystis_salinarum_1963
EP00191_Tetraselmis_sp_GSL018_24420
EP00191_Tetraselmis_sp_GSL018_17261
EP00192_Tetraselmis_astigmatica_4446
EP00194_Tetraselmis_striata_847
EP00208_Stichococcus_sp_RCC1054_1551
EP00206_Coccomyxa_subellipsoidea_3058
EP00204_Picochlorum_oklahomense_1833
EP00205_Picochlorum_sp_RCC944_662
EP00203_Chlorella_variabilis_282
EP00207_Helicosporidium_sp_ATCC50920_4667
EP00210_Caulerpa_lentillifera_8921
EP00198_Chlamydomonas_reinhardtii_1065
EP00202_Volvox_carteri_999
EP00199_Chlamydomonas_sp_CCMP681_1631
EP00195_Chlamydomonas_chlamydogama_297
EP00200_Dunaliella_tertiolecta_13917
EP00200_Dunaliella_tertiolecta_15670
EP00588_Corethron_pennatum_185
EP00588_Corethron_pennatum_29233
EP00566_Odontella_aurita_12990
EP00545_Synedropsis_sp_CCMP1620_5240
EP00038_Savillea_parva_17287
EP00435_Cladocopium_sp_Y103_76755
EP00439_Symbiodinium_sp_Y106_89182
EP00050_Salpingoeca_kvevrii_9411
EP00634_Sargassococcus_sp_CCMP2135_12575
EP00112_Aurelia_sp_Birch-Aquarium-sp1_7242
EP00656_Telonema_subtile_16000
EP00418_Pyrodinium_bahamense_42163
EP00418_Pyrodinium_bahamense_51600
EP00418_Pyrodinium_bahamense_65842
EP00411_Alexandrium_monilatum_111116
EP00410_Alexandrium_tamarense_36088
EP00403_Amphidinium_massartii_13500
EP00764_Aduncisulcus_paluster_1927
EP00290_Baffinella_sp_CCMP2293_9258
0.8

Fig. S40

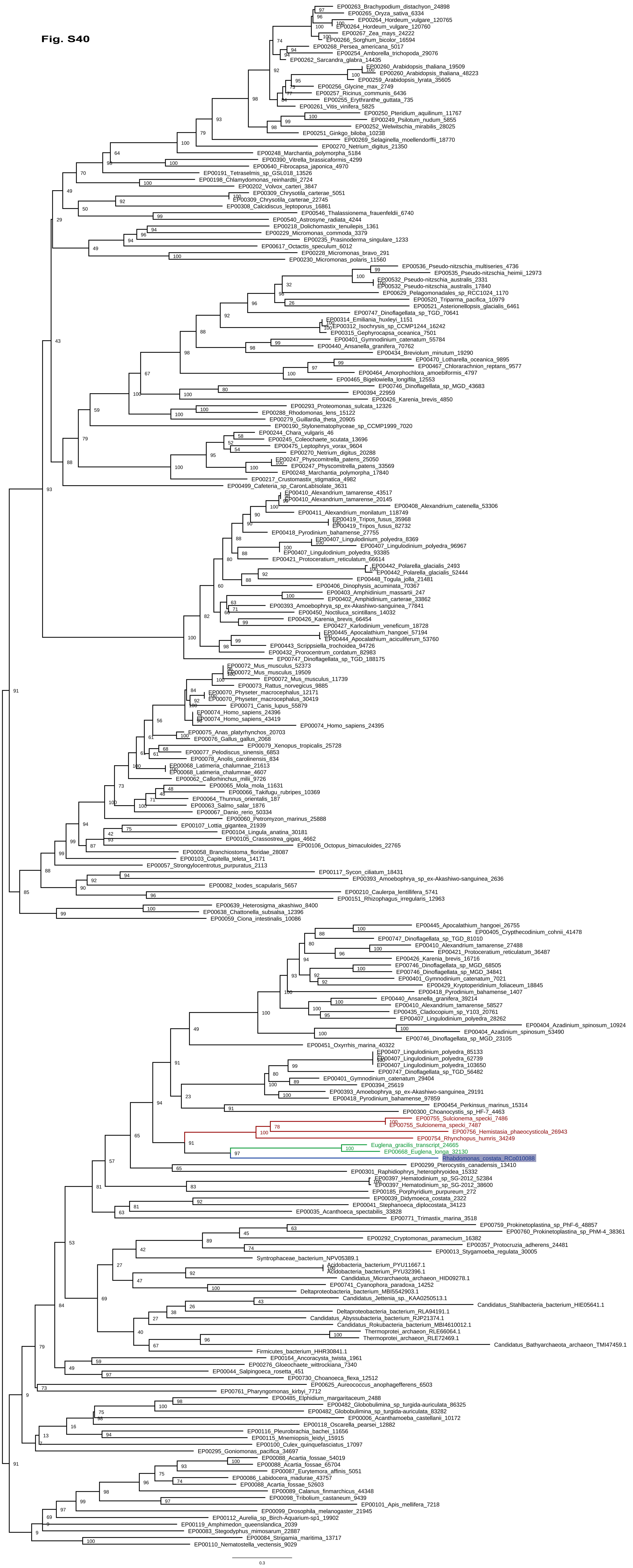
EP00263_Brachypodium_distachyon_24898
EP00265_Oryza_sativa_6334
EP00264_Hordeum_vulgare_120765
EP00264_Hordeum_vulgare_120760
EP00267_Zea_mays_24222
EP00266_Sorghum_bicolor_16594
EP00268_Persea_americana_5017
EP00254_Amborella_trichopoda_29076
EP00262_Sarcandra_glabra_14435
EP00260_Arabidopsis_thaliana_19509
EP00260_Arabidopsis_thaliana_48223
EP00259_Arabidopsis_lyrata_35605
EP00256_Glycine_max_2749
EP00257_Ricinus_communis_6436
EP00255_Erythranthe_guttata_735
EP00261_Vitis_vinifera_5825
EP00250_Pteridium_aquilinum_11767
EP00249_Psilotum_nudum_5855
EP00252_Welwitschia_mirabilis_28025
EP00251_Ginkgo_biloba_10238
EP00269_Selaginella_moellendorffii_18770
EP00270_Netrium_digitus_21350
EP00248_Marchantia_polymorpha_5184
EP00390_Vitrella_brassicaformis_4299
EP00640_Fibrocapsa_japonica_4970
EP00191_Tetraselmis_sp_GSL018_13526
EP00198_Chlamydomonas_reinhardtii_2724
EP00202_Volvox_carteri_3847
EP00309_Chrysotila_carterae_5051
EP00309_Chrysotila_carterae_22745
EP00308_Calcidiscus_leptoporus_16861
EP00546_Thalassionema_frauenfeldii_6740
EP00540_Astrosyne_radiata_4244
EP00218_Dolichomastix_tenuilepis_1361
EP00229_Micromonas_commoda_3379
EP00235_Prasinoderma_singulare_1233
EP00617_Octactis_speculum_6012
EP00228_Micromonas_bravo_291
EP00230_Micromonas_polaris_11560
EP00536_Pseudo-nitzschia_multiseries_4736
EP00535_Pseudo-nitzschia_heimii_12973
EP00532_Pseudo-nitzschia_australis_2331
EP00532_Pseudo-nitzschia_australis_17840
EP00629_Pelagomonadales_sp_RCC1024_1170
EP00520_Triparma_pacifica_10979
EP00521_Asterionellopsis_glacialis_6461
EP00747_Dinoflagellata_sp_TGD_70641
EP00314_Emiliania_huxleyi_1151
EP00312_Isochrysis_sp_CCMP1244_16242
EP00315_Gephyrocapsa_oceanica_7501
EP00401_Gymnodinium_catenatum_55784
EP00440_Ansanella_granifera_70762
EP00434_Breviolum_minutum_19290
EP00470_Lotharella_oceanica_9895
EP00467_Chlorarachnion_reptans_9577
EP00464_Amorphochlora_amoebiformis_4797
EP00465_Bigelowiella_longifila_12553
EP00746_Dinoflagellata_sp_MGD_43683
EP00394_22959
EP00426_Karenia_brevis_4850
EP00293_Proteomonas_sulcata_12326
EP00288_Rhodomonas_lens_15122
EP00279_Guillardia_theta_20905
EP00190_Stylonematophyceae_sp_CCMP1999_7020
EP00244_Chara_vulgaris_46
EP00245_Coleochaete_scutata_13696
EP00475_Leptophrys_vorax_9604
EP00270_Netrium_digitus_20288
EP00247_Physcomitrella_patens_25050
EP00247_Physcomitrella_patens_33569
EP00248_Marchantia_polymorpha_17840
EP00217_Crustomastix_stigmatica_4982
EP00499_Cafeteria_sp_CaronLabIsolate_3631
EP00410_Alexandrium_tamarense_43517
EP00410_Alexandrium_tamarense_20145
EP00408_Alexandrium_catenella_53306
EP00411_Alexandrium_monilatum_118749
EP00419_Tripos_fusus_35968
EP00419_Tripos_fusus_82732
EP00418_Pyrodinium_bahamense_27755
EP00407_Lingulodinium_polyedra_8369
EP00407_Lingulodinium_polyedra_96967
EP00407_Lingulodinium_polyedra_93385
EP00421_Protoceratium_reticulatum_66614
EP00442_Polarella_glacialis_2493
EP00442_Polarella_glacialis_52444
EP00448_Togula_jolla_21481
EP00406_Dinophysis_acuminata_70367
EP00403_Amphidinium_massartii_247
EP00402_Amphidinium_carterae_33862
EP00393_Amoebophrya_sp_ex-Akashiwo-sanguinea_77841
EP00450_Noctiluca_scintillans_14032
EP00426_Karenia_brevis_66454
EP00427_Karlodinium_veneficum_18728
EP00445_Apocalathium_hangoei_57194
EP00444_Apocalathium_aciculiferum_53760
EP00443_Scrippsiella_trochoidea_94726
EP00432_Prorocentrum_cordatum_82983
EP00747_Dinoflagellata_sp_TGD_188175
EP00072_Mus_musculus_52373
EP00072_Mus_musculus_19509
EP00072_Mus_musculus_11739
EP00073_Rattus_norvegicus_9885
EP00070_Physeter_macrocephalus_12171
EP00070_Physeter_macrocephalus_30419
EP00071_Canis_lupus_55879
EP00074_Homo_sapiens_24396
EP00074_Homo_sapiens_43419
EP00074_Homo_sapiens_24395
EP00075_Anas_platyrhynchos_20703
EP00076_Gallus_gallus_2068
EP00079_Xenopus_tropicalis_25728
EP00077_Pelodiscus_sinensis_6853
EP00078_Anolis_carolinensis_834
EP00068_Latimeria_chalumnae_21613
EP00068_Latimeria_chalumnae_4607
EP00062_Callorhinchus_milii_9726
EP00065_Mola_mola_11631
EP00066_Takifugu_rubripes_10369
EP00064_Thunnus_orientalis_187
EP00063_Salmo_salar_1876
EP00067_Danio_rerio_50334
EP00060_Petromyzon_marinus_25888
EP00107_Lottia_gigantea_21939
EP00104_Lingula_anatina_30181
EP00105_Crassostrea_gigas_4662
EP00106_Octopus_bimaculoides_22765
EP00058_Branchiostoma_floridae_28087
EP00103_Capitella_teleta_14171
EP00057_Strongylocentrotus_purpuratus_2113
EP00117_Sycon_ciliatum_18431
EP00393_Amoebophrya_sp_ex-Akashiwo-sanguinea_2636
EP00082_Ixodes_scapularis_5657
EP00210_Caulerpa_lentillifera_5741
EP00151_Rhizophagus_irregularis_12963
EP00639_Heterosigma_akashiwo_8400
EP00638_Chattonella_subsalsa_12396
EP00059_Ciona_intestinalis_10086
EP00445_Apocalathium_hangoei_26755
EP00405_Crypthecodinium_cohnii_41478
EP00747_Dinoflagellata_sp_TGD_81010
EP00410_Alexandrium_tamarense_27488
EP00421_Protoceratium_reticulatum_36487
EP00426_Karenia_brevis_16716
EP00746_Dinoflagellata_sp_MGD_68505
EP00746_Dinoflagellata_sp_MGD_34841
EP00401_Gymnodinium_catenatum_7021
EP00429_Kryptoperidinium_foliaceum_18845
EP00418_Pyrodinium_bahamense_1407
EP00440_Ansanella_granifera_39214
EP00410_Alexandrium_tamarense_58527
EP00435_Cladocopium_sp_Y103_20761
EP00407_Lingulodinium_polyedra_28262
EP00404_Azadinium_spinosum_10924
EP00404_Azadinium_spinosum_53490
EP00746_Dinoflagellata_sp_MGD_23105
EP00451_Oxyrrhis_marina_40322
EP00407_Lingulodinium_polyedra_85133
EP00407_Lingulodinium_polyedra_62739
EP00407_Lingulodinium_polyedra_103650
EP00747_Dinoflagellata_sp_TGD_56482
EP00401_Gymnodinium_catenatum_29404
EP00394_25619
EP00393_Amoebophrya_sp_ex-Akashiwo-sanguinea_29191
EP00418_Pyrodinium_bahamense_97859
EP00454_Perkinsus_marinus_15314
EP00300_Choanocystis_sp_HF-7_4463
EP00755_Sulcionema_specki_7486
EP00755_Sulcionema_specki_7487
EP00756_Hemistasia_phaeocysticola_26943
EP00754_Rhynchopus_humris_34249
Euglena_gracilis_transcript_24665
EP00668_Euglena_longa_32130
Rhabdomonas_costata_RCo010088
EP00299_Pterocystis_canadensis_13410
EP00301_Raphidiophrys_heterophryoidea_15332
EP00397_Hematodinium_sp_SG-2012_52384
EP00397_Hematodinium_sp_SG-2012_38600
EP00185_Porphyridium_purpureum_272
EP00039_Didymoeca_costata_2322
EP00041_Stephanoeca_diplocostata_34123
EP00035_Acanthoeca_spectabilis_33828
EP00771_Trimastix_marina_3518
EP00759_Prokinetoplastina_sp_PhF-6_48857
EP00760_Prokinetoplastina_sp_PhM-4_38361
EP00292_Cryptomonas_paramecium_16382
EP00357_Protocruzia_adherens_24481
EP00013_Stygamoeba_regulata_30005
Syntrophaceae_bacterium_NPV05389.1
Acidobacteria_bacterium_PYU11667.1
Acidobacteria_bacterium_PYU32396.1
Candidatus_Micrarchaeota_archaeon_HID09278.1
EP00741_Cyanophora_paradoxa_14252
Deltaproteobacteria_bacterium_MBI5542903.1
Candidatus_Jettenia_sp._KAA0250513.1
Candidatus_Stahlbacteria_bacterium_HIE05641.1
Deltaproteobacteria_bacterium_RLA94191.1
Candidatus_Abyssubacteria_bacterium_RJP21374.1
Candidatus_Rokubacteria_bacterium_MBI4610012.1
Thermoprotei_archaeon_RLE66064.1
Thermoprotei_archaeon_RLE72469.1
Candidatus_Bathyarchaeota_archaeon_TMI47459.1
Firmicutes_bacterium_HHR30841.1
EP00164_Ancoracysta_twista_1961
EP00276_Gloeochaete_wittrockiana_7340
EP00044_Salpingoeca_rosetta_451
EP00730_Choanoeca_flexa_12512
EP00625_Aureococcus_anophagefferens_6503
EP00761_Pharyngomonas_kirbyi_7712
EP00485_Elphidium_margaritaceum_2488
EP00482_Globobulimina_sp_turgida-auriculata_86325
EP00482_Globobulimina_sp_turgida-auriculata_83282
EP00006_Acanthamoeba_castellanii_10172
EP00118_Oscarella_pearsei_12882
EP00116_Pleurobrachia_bachei_11656
EP00115_Mnemiopsis_leidyi_15915
EP00100_Culex_quinquefasciatus_17097
EP00295_Goniomonas_pacifica_34697
EP00088_Acartia_fossae_54019
EP00088_Acartia_fossae_65704
EP00087_Eurytemora_affinis_5051
EP00086_Labidocera_madurae_43757
EP00088_Acartia_fossae_52603
EP00089_Calanus_finmarchicus_44348
EP00098_Tribolium_castaneum_9439
EP00101_Apis_mellifera_7218
EP00099_Drosophila_melanogaster_21945
EP00112_Aurelia_sp_Birch-Aquarium-sp1_19902
EP00119_Amphimedon_queenslandica_2039
EP00083_Stegodyphus_mimosarum_22887
EP00084_Strigamia_maritima_13717
EP00110_Nematostella_vectensis_9029
0.3

Fig. S41

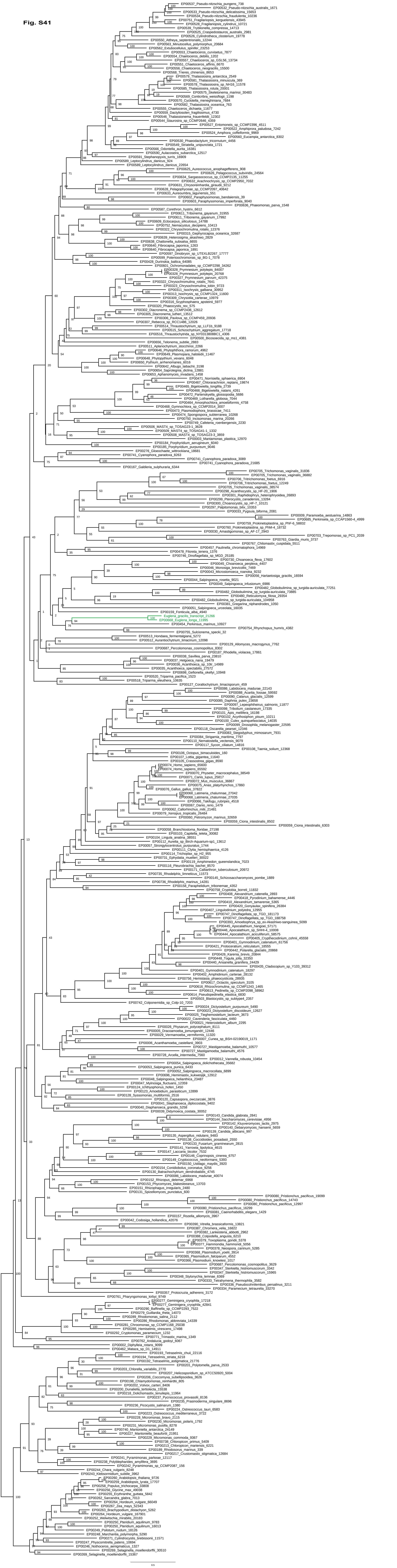

Fig. S42

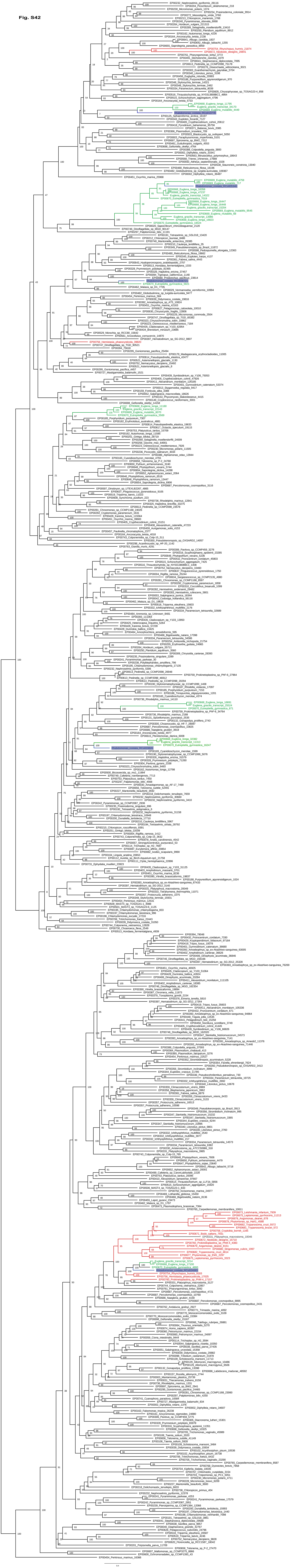

Fig. S43

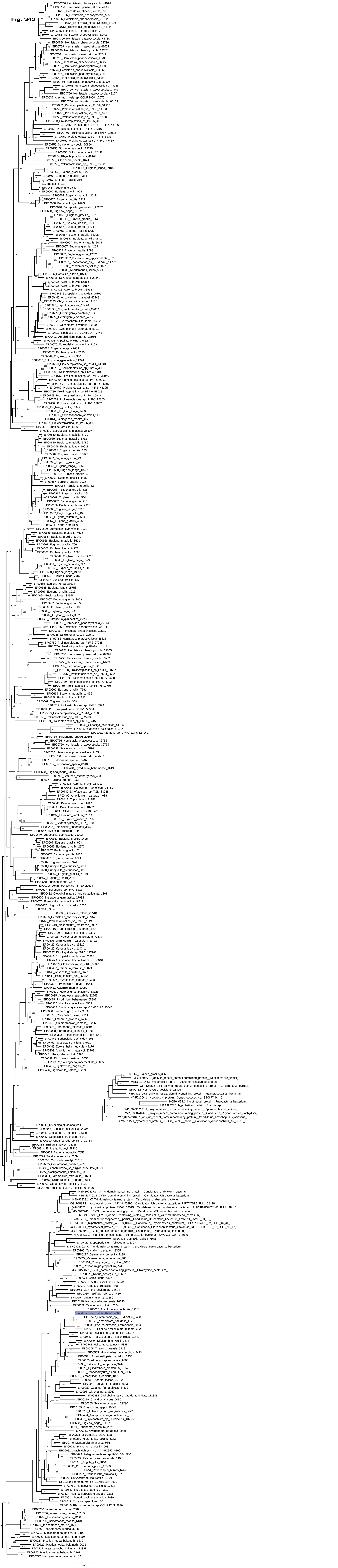

Fig. S44

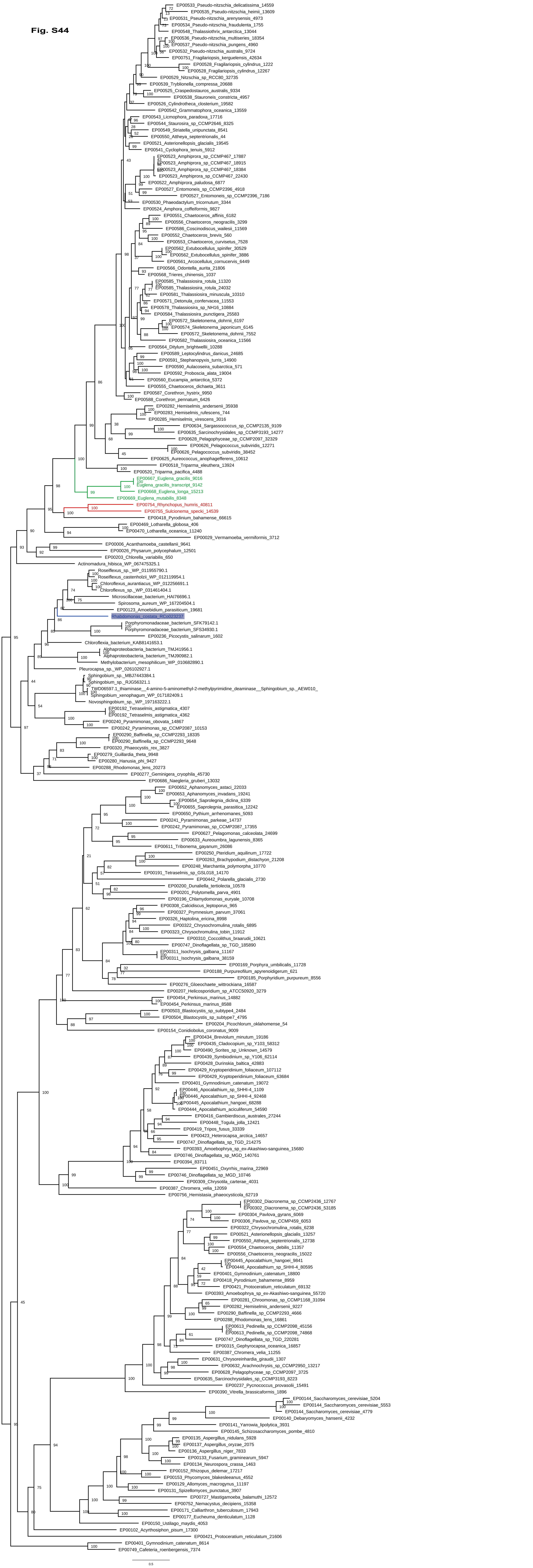

Fig. S45

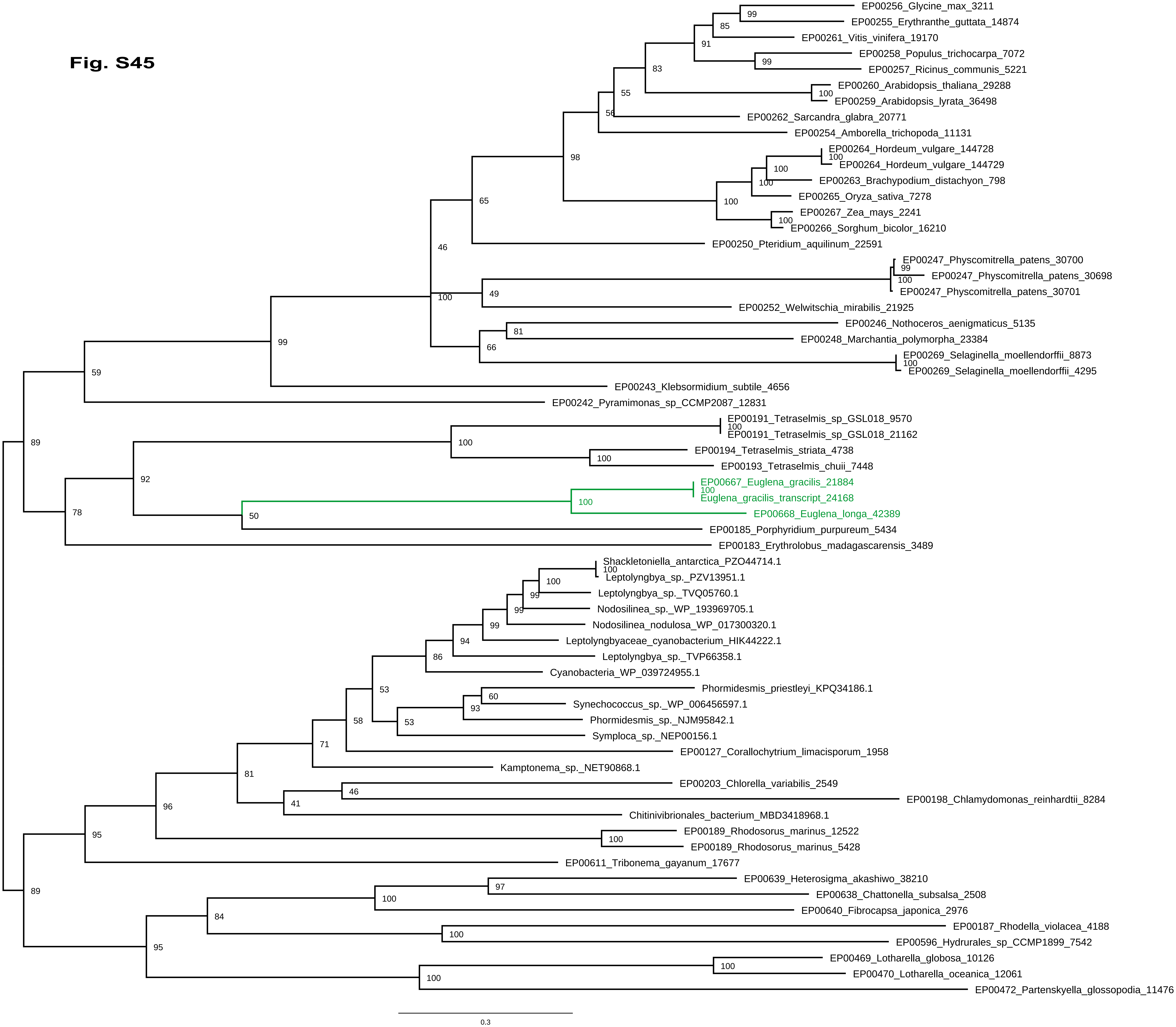
EP00256_Glycine_max_3211
EP00255_Erythranthe_guttata_14874
EP00261_Vitis_vinifera_19170
EP00258_Populus_trichocarpa_7072
EP00257_Ricinus_communis_5221
EP00260_Arabidopsis_thaliana_29288
EP00259_Arabidopsis_lyrata_36498
EP00262_Sarcandra_glabra_20771
EP00254_Amborella_trichopoda_11131
EP00264_Hordeum_vulgare_144728
EP00264_Hordeum_vulgare_144729
EP00263_Brachypodium_distachyon_798
EP00265_Oryza_sativa_7278
EP00267_Zea_mays_2241
EP00266_Sorghum_bicolor_16210
EP00250_Pteridium_aquilinum_22591
EP00247_Physcomitrella_patens_30700
EP00247_Physcomitrella_patens_30698
EP00247_Physcomitrella_patens_30701
EP00252_Welwitschia_mirabilis_21925
EP00246_Nothoceros_aenigmaticus_5135
EP00248_Marchantia_polymorpha_23384
EP00269_Selaginella_moellendorffii_8873
EP00269_Selaginella_moellendorffii_4295
EP00243_Klebsormidium_subtile_4656
EP00242_Pyramimonas_sp_CCMP2087_12831
EP00191_Tetraselmis_sp_GSL018_9570
EP00191_Tetraselmis_sp_GSL018_21162
EP00194_Tetraselmis_striata_4738
EP00193_Tetraselmis_chuii_7448
EP00667_Euglena_gracilis_21884
Euglena_gracilis_transcript_24168
EP00668_Euglena_longa_42389
EP00185_Porphyridium_purpureum_5434
EP00183_Erythrolobus_madagascarensis_3489
Shackletoniella_antarctica_PZO44714.1
Leptolyngbya_sp._PZV13951.1
Leptolyngbya_sp._TVQ05760.1
Nodosilinea_sp._WP_193969705.1
Nodosilinea_nodulosa_WP_017300320.1
Leptolyngbyaceae_cyanobacterium_HIK44222.1
Leptolyngbya_sp._TVP66358.1
Cyanobacteria_WP_039724955.1
Phormidesmis_priestleyi_KPQ34186.1
Synechococcus_sp._WP_006456597.1
Phormidesmis_sp._NJM95842.1
Symploca_sp._NEP00156.1
EP00127_Corallochytrium_limacisporum_1958
Kamptonema_sp._NET90868.1
EP00203_Chlorella_variabilis_2549
EP00198_Chlamydomonas_reinhardtii_8284
Chitinivibrionales_bacterium_MBD3418968.1
EP00189_Rhodosorus_marinus_12522
EP00189_Rhodosorus_marinus_5428
EP00611_Tribonema_gayanum_17677
EP00639_Heterosigma_akashiwo_38210
EP00638_Chattonella_subsalsa_2508
EP00640_Fibrocapsa_japonica_2976
EP00187_Rhodella_violacea_4188
EP00596_Hydrurales_sp_CCMP1899_7542
EP00469_Lotharella_globosa_10126
EP00470_Lotharella_oceanica_12061
EP00472_Partenskyella_glossopodia_11476
0.3
