# Supplementary material for: Heterotrophic euglenid Rhabdomonas costata resembles its phototrophic relatives in many aspects of molecular and cell biology
Source: Sci Rep. 2021 Jun 22;11:13070. doi: 10.1038/s41598-021-92174-3 (PMC8219788; doi:10.1038/s41598-021-92174-3)
Supplement: Supplementary file 5 — Supplementary Figures S46-S53. [file 41598_2021_92174_MOESM5_ESM.pdf]

**Fig. S46: Phylogenetic tree of 5-aminolevulinate synthase (ALAS).** The tree was constructed in IQTree using the LG+R6 model selected by Bayesian information criterion from a trimmed and manuály inspected alignment containing 341 amino acid positions. The values at the nodes represent ultrafast bootstraps from 1,000 repetitions. Euglenophyte sequences are shown in green, *Rhabdomonas costata* sequences in blue. The figure was created in FigTree v 1.4.4.

**Fig. S47: Phylogenetic tree of porphobilinogen synthase (ALAD).** The tree was constructed in IQTree using the LG+R6 model selected by Bayesian information criterion from a trimmed and manually inspected alignment containing 152 amino acid positions. The values 28 at the nodes represent ultrafast bootstraps from 1,000 repetitions. Euglenophyte sequences are shown in green, *Rhabdomonas costata* sequences in blue, other euglenozoa in red. The figure was created in FigTree v 1.4.4.

**Fig. S48: Phylogenetic tree of porphobilinogen deaminase (PBGD).** The tree was constructed in IQTree using the LG+I+G4 model selected by Bayesian information criterion from a trimmed and manually inspected alignment containing 269 amino acid positions. The values at the nodes represent ultrafast bootstraps from 1,000 repetitions. Euglenophyte sequences are shown in green, *Rhabdomonas costata* sequences in blue. The figure was created in FigTree v 1.4.4.

**Fig. S49: Phylogenetic tree of uroporphyrinogen synthase (UROS).** The tree was constructed in IQTree using the site heterogeneous PMSF model from a trimmed and manually inspected alignment 185 amino acid positions. The values at the nodes represent ultrafast bootstraps from 1,000 repetitions. Euglenophyte sequences are shown in green, *Rhabdomonas costata* sequences in blue. The figure was created in FigTree v 1.4.4.

**Fig. S50: Phylogenetic tree of uroporphyrinogen decarboxylase (UROD).** The tree was constructed in IQ-Tree using the LG+R6 model selected by Bayesian information criterion from a trimmed and manually inspected alignment containing 255 amino acid positions. The values at the nodes represent ultrafast bootstraps from 1,000 repetitions. Euglenophyte sequences are shown in green, *Rhabdomonas costata* sequences in blue, other euglenozoa in red. The figure was created in FigTree v 1.4.4.

**Fig. S51: Phylogenetic tree of coproporphyrinogen oxidase (CPOX).** The tree was constructed in IQ-Tree using the LG+F+R6 model selected by Bayesian information criterion from a trimmed and manually inspected alignment containing 237 amino acid positions. The values at the nodes represent ultrafast bootstraps from 1,000 repetitions. Euglenophyte sequences are shown in green, *Rhabdomonas costata* sequences in blue. The figure was created in FigTree v 1.4.4.

**Fig. S52: Phylogenetic tree of protoporphyrinogen oxidase (PPOX).** The tree was constructed in IQ-Tree using the LG+I+G4 model selected by Bayesian information criterion from a trimmed and manually inspected alignment containing 314 amino acid positions. The values at the nodes represent ultrafast bootstraps from 1,000 repetitions. Euglenophyte sequences are shown in green, *Rhabdomonas costata* sequences in blue, other euglenozoa in red. The figure was created in FigTree v 1.4.4.

**Fig. S53: Phylogenetic tree of ferrochelatase (FECH).** The tree was constructed in IQ-Tree using the LG+I+G4 model selected by Bayesian information criterion from a trimmed and manually inspected alignment containing 232 amino acid positions. The values at the nodes represent ultrafast bootstraps from 1,000 repetitions. Euglenophyte sequences are shown in green, *Rhabdomonas costata* sequences in blue, other euglenozoa in red. The figure was created in FigTree v 1.4.4.

**Fig. S46: ALAS**

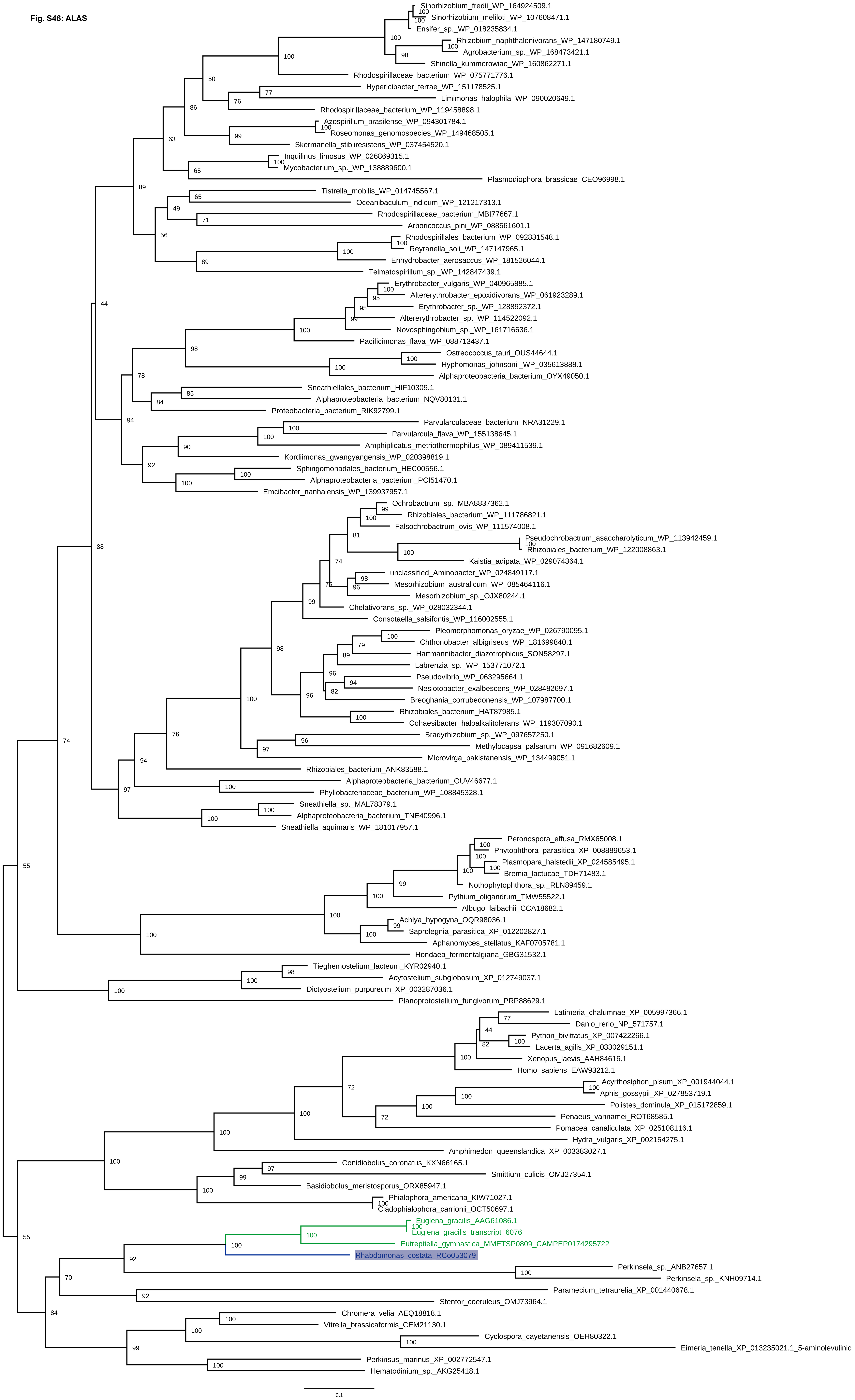

Sinorhizobium_fredii_WP_164924509.1
Sinorhizobium_meliloti_WP_107608471.1
Ensifer_sp._WP_018235834.1
Rhizobium_naphthalenivorans_WP_147180749.1
Agrobacterium_sp._WP_168473421.1
Shinella_kummerowiae_WP_160862271.1
Rhodospirillaceae_bacterium_WP_075771776.1
Hypericibacter_terrae_WP_151178525.1
Limimonas_halophila_WP_090020649.1
Rhodospirillaceae_bacterium_WP_119458898.1
Azospirillum_brasilense_WP_094301784.1
Roseomonas_genomospecies_WP_149468505.1
Skermanella_stibiiresistens_WP_037454520.1
Inquilinus_limosus_WP_026869315.1
Mycobacterium_sp._WP_138889600.1
Plasmodiophora_brassicae_CEO96998.1
Tistrella_mobilis_WP_014745567.1
Oceanibaculum_indicum_WP_121217313.1
Rhodospirillaceae_bacterium_MBI77667.1
Arboricoccus_pini_WP_088561601.1
Rhodospirillales_bacterium_WP_092831548.1
Reyranella_soli_WP_147147965.1
Enhydrobacter_aerosaccus_WP_181526044.1
Telmatospirillum_sp._WP_142847439.1
Erythrobacter_vulgaris_WP_040965885.1
Altererythrobacter_epoxidivorans_WP_061923289.1
Erythrobacter_sp._WP_128892372.1
Altererythrobacter_sp._WP_114522092.1
Novosphingobium_sp._WP_161716636.1
Pacificimonas_flava_WP_088713437.1
Ostreococcus_tauri_OUS44644.1
Hyphomonas_johnsonii_WP_035613888.1
Alphaproteobacteria_bacterium_OYX49050.1
Sneathiellales_bacterium_HIF10309.1
Alphaproteobacteria_bacterium_NQV80131.1
Proteobacteria_bacterium_RIK92799.1
Parvularculaceae_bacterium_NRA31229.1
Parvularcula_flava_WP_155138645.1
Amphiplicatus_metriothermophilus_WP_089411539.1
Kordiimonas_gwangyangensis_WP_020398819.1
Sphingomonadales_bacterium_HEC00556.1
Alphaproteobacteria_bacterium_PCI51470.1
Emcibacter_nanhaiensis_WP_139937957.1
Ochrobactrum_sp._MBA8837362.1
Rhizobiales_bacterium_WP_111786821.1
Falsochrobactrum_ovis_WP_111574008.1
Pseudochrobactrum_asaccharolyticum_WP_113942459.1
Rhizobiales_bacterium_WP_122008863.1
Kaistia_adipata_WP_029074364.1
unclassified_Aminobacter_WP_024849117.1
Mesorhizobium_australicum_WP_085464116.1
Mesorhizobium_sp._OJX80244.1
Chelativorans_sp._WP_028032344.1
Consotaella_salsifontis_WP_116002555.1
Pleomorphomonas_oryzae_WP_026790095.1
Chthonobacter_albigriseus_WP_181699840.1
Hartmannibacter_diazotrophicus_SON58297.1
Labrenzia_sp._WP_153771072.1
Pseudovibrio_WP_063295664.1
Nesiotobacter_exalbescens_WP_028482697.1
Breoghania_corrubedonensis_WP_107987700.1
Rhizobiales_bacterium_HAT87985.1
Cohaesibacter_haloalkalitolerans_WP_119307090.1
Bradyrhizobium_sp._WP_097657250.1
Methylocapsa_palsarum_WP_091682609.1
Microvirga_pakistanensis_WP_134499051.1
Rhizobiales_bacterium_ANK83588.1
Alphaproteobacteria_bacterium_OUV46677.1
Phyllobacteriaceae_bacterium_WP_108845328.1
Sneathiella_sp._MAL78379.1
Alphaproteobacteria_bacterium_TNE40996.1
Sneathiella_aquimaris_WP_181017957.1
Peronospora_effusa_RMX65008.1
Phytophthora_parasitica_XP_008889653.1
Plasmopara_halstedii_XP_024585495.1
Bremia_lactucae_TDH71483.1
Nothophytophthora_sp._RLN89459.1
Pythium_oligandrum_TMW55522.1
Albugo_laibachii_CCA18682.1
Achlya_hypogyna_OQR98036.1
Saprolegnia_parasitica_XP_012202827.1
Aphanomyces_stellatus_KAF0705781.1
Hondaea_fermentalgiana_GBG31532.1
Tieghemostelium_lacteum_KYR02940.1
Acytostelium_subglobosum_XP_012749037.1
Dictyostelium_purpureum_XP_003287036.1
Planoprotostelium_fungivorum_PRP88629.1
Latimeria_chalumnae_XP_005997366.1
Danio_rerio_NP_571757.1
Python_bivittatus_XP_007422266.1
Lacerta_agilis_XP_033029151.1
Xenopus_laevis_AAH84616.1
Homo_sapiens_EAW93212.1
Acyrthosiphon_pisum_XP_001944044.1
Aphis_gossypii_XP_027853719.1
Polistes_dominula_XP_015172859.1
Penaeus_vannamei_ROT68585.1
Pomacea_canaliculata_XP_025108116.1
Hydra_vulgaris_XP_002154275.1
Amphimedon_queenslandica_XP_003383027.1
Conidiobolus_coronatus_KXN66165.1
Smittium_culicis_OMJ27354.1
Basidiobolus_meristosporus_ORX85947.1
Phialophora_americana_KIW71027.1
Cladophialophora_carrionii_OCT50697.1
Euglena_gracilis_AAG61086.1
Euglena_gracilis_transcript_6076
Eutreptiella_gymnastica_MMETSP0809_CAMPEP0174295722
Rhabdomonas_costata_RCo053079
Perkinsela_sp._ANB27657.1
Perkinsela_sp._KNH09714.1
Paramecium_tetraurelia_XP_001440678.1
Stentor_coeruleus_OMJ73964.1
Chromera_velia_AEQ18818.1
Vitrella_brassicaformis_CEM21130.1
Cyclospora_cayetanensis_OEH80322.1
Eimeria_tenella_XP_013235021.1_5-aminolevulinic
Perkinsus_marinus_XP_002772547.1
Hematodinium_sp._AKG25418.1
0.1

Fig. S47: ALAD

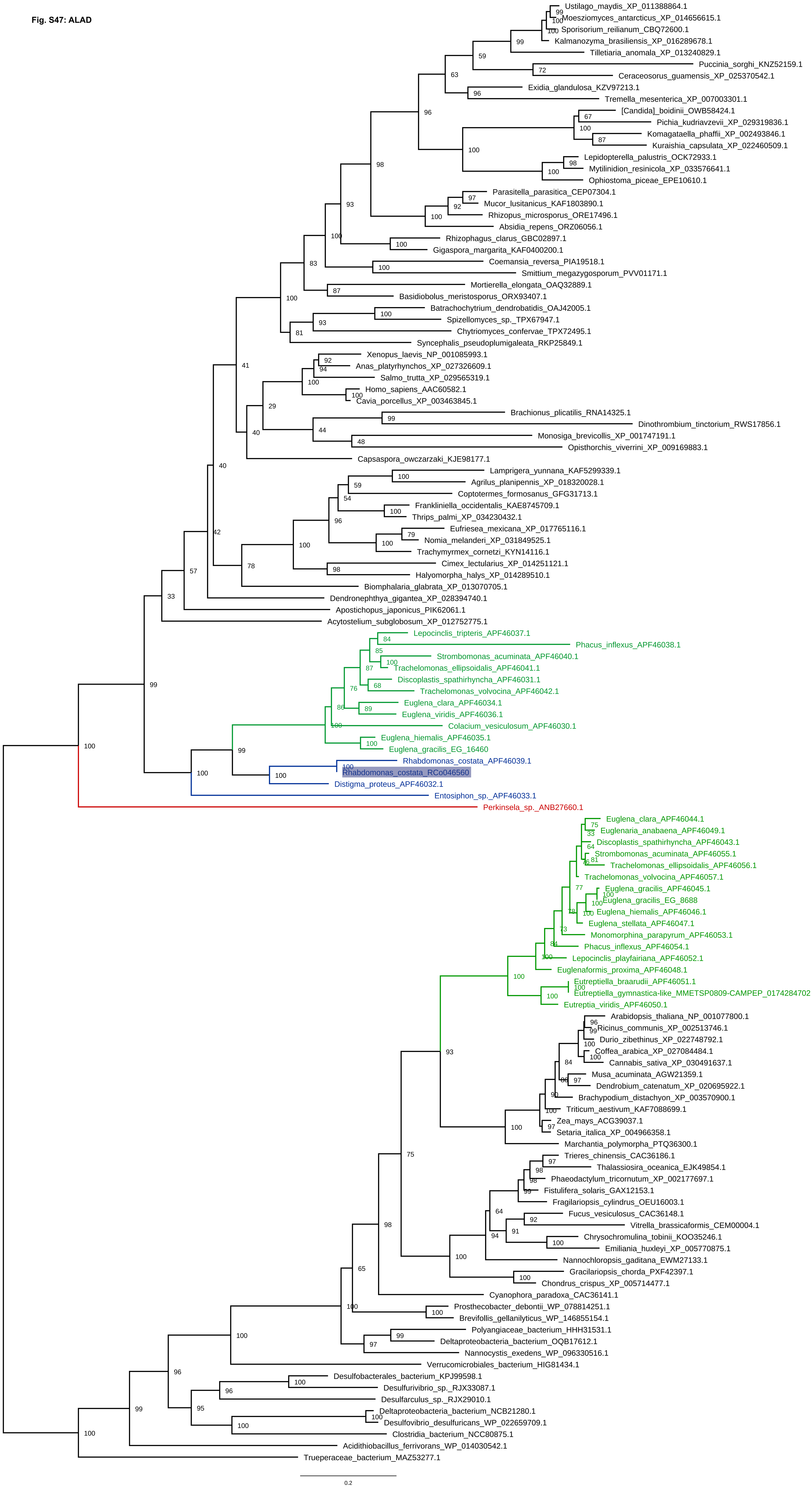

Ustilago_maydis_XP_011388864.1
Moesziomyces_antarcticus_XP_014656615.1
Sporisorium_reilianum_CBQ72600.1
Kalmanozyma_brasiliensis_XP_016289678.1
Tilletiaria_anomala_XP_013240829.1
Puccinia_sorghi_KNZ52159.1
Ceraceosorus_guamensis_XP_025370542.1
Exidia_glandulosa_KZV97213.1
Tremella_mesenterica_XP_007003301.1
[Candida]_boidinii_OWB58424.1
Pichia_kudriavzevii_XP_029319836.1
Komagataella_phaffii_XP_002493846.1
Kuraishia_capsulata_XP_022460509.1
Lepidopterella_palustris_OCK72933.1
Mytilinidion_resinicola_XP_033576641.1
Ophiostoma_piceae_EPE10610.1
Parasitella_parasitica_CEP07304.1
Mucor_lusitanicus_KAF1803890.1
Rhizopus_microsporus_ORE17496.1
Absidia_repens_ORZ06056.1
Rhizophagus_clarus_GBC02897.1
Gigaspora_margarita_KAF0400200.1
Coemansia_reversa_PIA19518.1
Smittium_megazygosporum_PVV01171.1
Mortierella_elongata_OAQ32889.1
Basidiobolus_meristosporus_ORX93407.1
Batrachochytrium_dendrobatidis_OAJ42005.1
Spizellomyces_sp._TPX67947.1
Chytriomyces_confervae_TPX72495.1
Syncephalis_pseudoplumigaleata_RKP25849.1
Xenopus_laevis_NP_001085993.1
Anas_platyrhynchos_XP_027326609.1
Salmo_trutta_XP_029565319.1
Homo_sapiens_AAC60582.1
Cavia_porcellus_XP_003463845.1
Brachionus_plicatilis_RNA14325.1
Dinothrombium_tinctorium_RWS17856.1
Monosiga_brevicollis_XP_001747191.1
Opisthorchis_viverrini_XP_009169883.1
Capsaspora_owczarzaki_KJE98177.1
Lamprigera_yunnana_KAF5299339.1
Agrilus_planipennis_XP_018320028.1
Coptotermes_formosanus_GFG31713.1
Frankliniella_occidentalis_KAE8745709.1
Thrips_palmi_XP_034230432.1
Eufriesea_mexicana_XP_017765116.1
Nomia_melanderi_XP_031849525.1
Trachymyrmex_cornetzi_KYN14116.1
Cimex_lectularius_XP_014251121.1
Halyomorpha_halys_XP_014289510.1
Biomphalaria_glabrata_XP_013070705.1
Dendronephthya_gigantea_XP_028394740.1
Apostichopus_japonicus_PIK62061.1
Acytostelium_subglobosum_XP_012752775.1
Lepocinclis_tripteris_APF46037.1
Phacus_inflexus_APF46038.1
Strombomonas_acuminata_APF46040.1
Trachelomonas_ellipsoidalis_APF46041.1
Discoplastis_spathirhycha_APF46031.1
Trachelomonas_volvocina_APF46042.1
Euglena_clara_APF46034.1
Euglena_viridis_APF46036.1
Colacium_vesiculosum_APF46030.1
Euglena_hiemalis_APF46035.1
Euglena_gracilis_EG_16460
Rhabdomonas_costata_APF46039.1
Rhabdomonas_costata_RCo046560
Distigma_proteus_APF46032.1
Entosiphon_sp._APF46033.1
Perkinsela_sp._ANB27660.1
Euglena_clara_APF46044.1
Euglenaria_anabaena_APF46049.1
Discoplastis_spathirhyncha_APF46043.1
Strombomonas_acuminata_APF46055.1
Trachelomonas_ellipsoidalis_APF46056.1
Trachelomonas_volvocina_APF46057.1
Euglena_gracilis_APF46045.1
Euglena_gracilis_EG_8688
Euglena_hiemalis_APF46046.1
Euglena_stellata_APF46047.1
Monomorphina_parapyrum_APF46053.1
Phacus_inflexus_APF46054.1
Lepocinclis_playfairiana_APF46052.1
Euglenaformis_proxima_APF46048.1
Eutreptiella_braarudii_APF46051.1
Eutreptiella_gymnastica-like_MMETSP0809-CAMPEP_0174284702
Eutreptia_viridis_APF46050.1
Arabidopsis_thaliana_NP_001077800.1
Ricinus_communis_XP_002513746.1
Durio_zibethinus_XP_022748792.1
Coffea_arabica_XP_027084484.1
Cannabis_sativa_XP_030491637.1
Musa_acuminata_AGW21359.1
Dendrobium_catenatum_XP_020695922.1
Brachypodium_distachyon_XP_003570900.1
Triticum_aestivum_KAF7088699.1
Zea_mays_ACG39037.1
Setaria_italica_XP_004966358.1
Marchantia_polymorpha_PTQ36300.1
Trieres_chinensis_CAC36186.1
Thalassiosira_oceanica_EJK49854.1
Phaeodactylum_tricornutum_XP_002177697.1
Fistulifera_solaris_GAX12153.1
Fragilariopsis_cylindrus_OEU16003.1
Fucus_vesiculosus_CAC36148.1
Vitrella_brassicaformis_CEM00004.1
Chrysochromulina_tobinii_KOO35246.1
Emiliania_huxleyi_XP_005770875.1
Nannochloropsis_gaditana_EWM27133.1
Gracilariopsis_chorda_PXF42397.1
Chondrus_crispus_XP_005714477.1
Cyanophora_paradoxa_CAC36141.1
Prosthecobacter_debontii_WP_078814251.1
Brevifollis_gellanilyticus_WP_146855154.1
Polyangiaceae_bacterium_HHH31531.1
Deltaproteobacteria_bacterium_OQB17612.1
Nannocystis_exedens_WP_096330516.1
Verrucomicrobiales_bacterium_HIG81434.1
Desulfobacterales_bacterium_KPJ99598.1
Desulfurivibrio_sp._RJX33087.1
Desulfarculus_sp._RJX29010.1
Deltaproteobacteria_bacterium_NCB21280.1
Desulfovibrio_desulfuricans_WP_022659709.1
Clostridia_bacterium_NCC80875.1
Acidithiobacillus_ferrivorans_WP_014030542.1
Trueperaceae_bacterium_MAZ53277.1
0.2

**Fig. S48: PBGD**

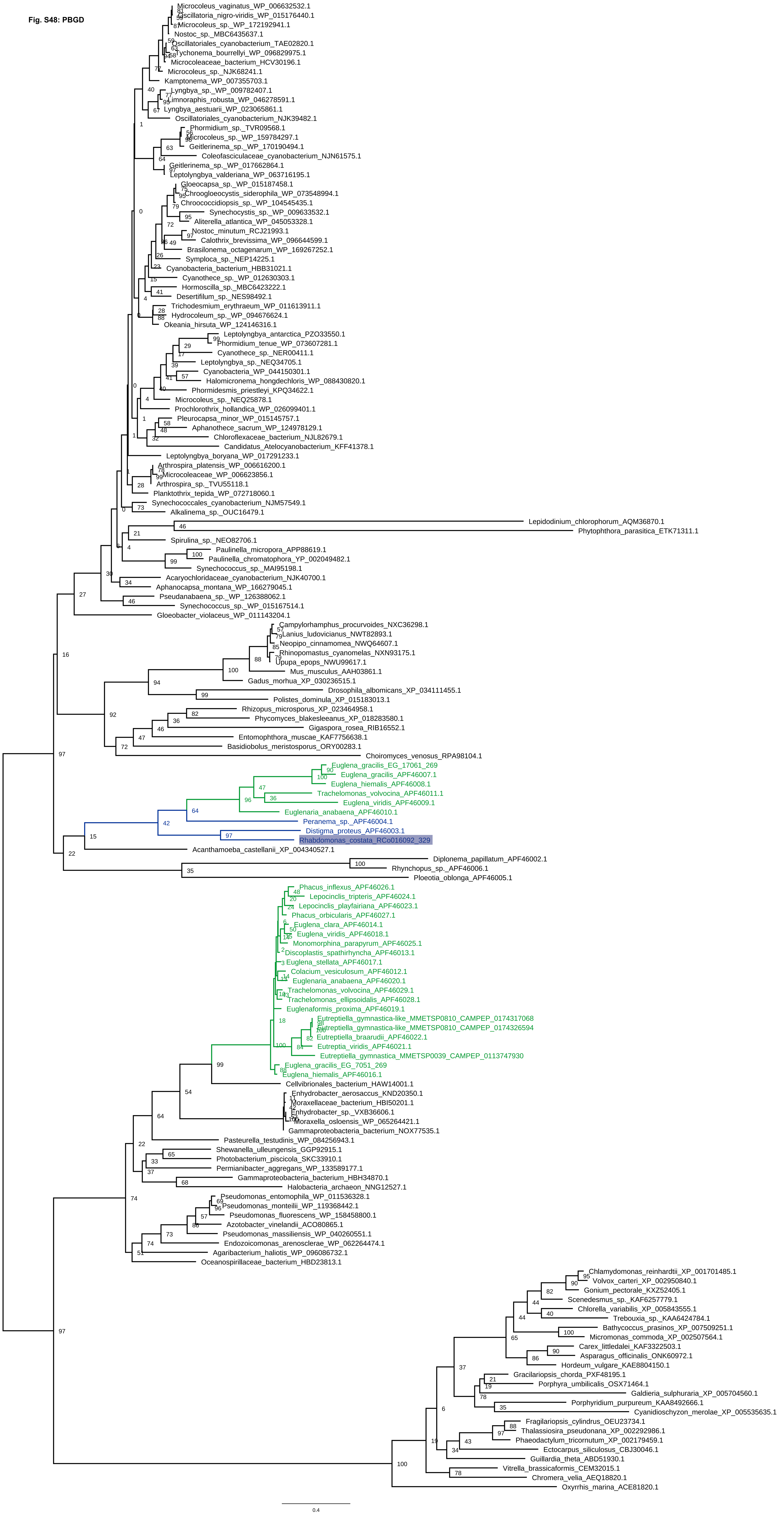
Microcoleus_vaginatus_WP_006632532.1
Oscillatoria_nigro-viridis_WP_015176440.1
Microcoleus_sp._WP_172192941.1
Nostoc_sp._MBC6435637.1
Oscillatoriales_cyanobacterium_TAE02820.1
Tychonema_bourrellyi_WP_096829975.1
Microcoleaceae_bacterium_HCV30196.1
Microcoleus_sp._NJK68241.1
Kamptonema_WP_007355703.1
Lyngbya_sp._WP_009782407.1
Limnoraphis_robusta_WP_046278591.1
Lyngbya_aestuarii_WP_023065861.1
Oscillatoriales_cyanobacterium_NJK39482.1
Phormidium_sp._TVR09568.1
Microcoleus_sp._WP_159784297.1
Geitlerinema_sp._WP_170190494.1
Coleofasciculaceae_cyanobacterium_NJN61575.1
Geitlerinema_sp._WP_017662864.1
Leptolyngbya_valderiana_WP_063716195.1
Gloeocapsa_sp._WP_015187458.1
Chroogloeocystis_siderophila_WP_073548994.1
Chroococcidiopsis_sp._WP_104545435.1
Synechocystis_sp._WP_009633532.1
Aliterella_atlantica_WP_045053328.1
Nostoc_minutum_RCJ21993.1
Calothrix_brevissima_WP_096644599.1
Brasilonema_octagenarum_WP_169267252.1
Symploca_sp._NEP14225.1
Cyanobacteria_bacterium_HBB31021.1
Cyanothece_sp._WP_012630303.1
Hormoscilla_sp._MBC6423222.1
Desertifilum_sp._NES98492.1
Trichodesmium_erythraeum_WP_011613911.1
Hydrocoleum_sp._WP_094676624.1
Okeania_hirsuta_WP_124146316.1
Leptolyngbya_antarctica_PZO33550.1
Phormidium_tenue_WP_073607281.1
Cyanothece_sp._NER00411.1
Leptolyngbya_sp._NEQ34705.1
Cyanobacteria_WP_044150301.1
Halomicronema_hongdechloris_WP_088430820.1
Phormidesmis_priestleyi_KPQ34622.1
Microcoleus_sp._NEQ25878.1
Prochlorothrix_hollandica_WP_026099401.1
Pleurocapsa_minor_WP_015145757.1
Aphanothece_sacrum_WP_124978129.1
Chloroflexaceae_bacterium_NJL82679.1
Candidatus_Atelocyanobacterium_KFF41378.1
Leptolyngbya_boryana_WP_017291233.1
Arthrospira_platensis_WP_006616200.1
Microcoleaceae_WP_006623856.1
Arthrospira_sp._TVU55118.1
Planktothrix_tepida_WP_072718060.1
Synechococcales_cyanobacterium_NJM57549.1
Alkalinema_sp._OUC16479.1
Lepidodinium_chlorophorum_AQM36870.1
Phytophthora_parasitica_ETK71311.1
Spirulina_sp._NEO82706.1
Paulinella_micropora_APP88619.1
Paulinella_chromatophora_YP_002049482.1
Synechococcus_sp._MAI95198.1
Acaryochloridaceae_cyanobacterium_NJK40700.1
Aphanocapsa_montana_WP_166279045.1
Pseudanabaena_sp._WP_126388062.1
Synechococcus_sp._WP_015167514.1
Gloeobacter_violaceus_WP_011143204.1
Campylorhamphus_procurvoides_NXC36298.1
Lanius_ludovicianus_NWT82893.1
Neopipo_cinnamomea_NWQ64607.1
Rhinopomastus_cyanomelas_NXN93175.1
Upupa_epops_NWU99617.1
Mus_musculus_AAH03861.1
Gadus_morhua_XP_030236515.1
Drosophila_albomicans_XP_034111455.1
Polistes_dominula_XP_015183013.1
Rhizopus_microsporus_XP_023464958.1
Phycomyces_blakesleeanus_XP_018283580.1
Gigaspora_rosea_RIB16552.1
Entomophthora_muscae_KAF7756638.1
Basidiobolus_meristosporus_ORY00283.1
Choiromyces_venosus_RPA98104.1
Euglena_gracilis_EG_17061_269
Euglena_gracilis_APF46007.1
Euglena_hiemalis_APF46008.1
Trachelomonas_volvocina_APF46011.1
Euglena_viridis_APF46009.1
Euglenaria_anabaena_APF46010.1
Peranema_sp._APF46004.1
Distigma_proteus_APF46003.1
Rhabdomonas_costata_RCo016092_329
Acanthamoeba_castellanii_XP_004340527.1
Diplonema_papillatum_APF46002.1
Rhynchopus_sp._APF46006.1
Ploeotia_oblonga_APF46005.1
Phacus_inflexus_APF46026.1
Lepocinclis_tripteris_APF46024.1
Lepocinclis_playfairiana_APF46023.1
Phacus_orbicularis_APF46027.1
Euglena_clara_APF46014.1
Euglena_viridis_APF46018.1
Monomorphina_parapyrum_APF46025.1
Discoplastis_spathirhyncha_APF46013.1
Euglena_stellata_APF46017.1
Colacium_vesiculosum_APF46012.1
Euglenaria_anabaena_APF46020.1
Trachelomonas_volvocina_APF46029.1
Trachelomonas_ellipsoidalis_APF46028.1
Euglenaformis_proxima_APF46019.1
Eutreptiella_gymnastica-like_MMETSP0810_CAMPEP_0174317068
Eutreptiella_gymnastica-like_MMETSP0810_CAMPEP_0174326594
Eutreptiella_braarudii_APF46022.1
Eutreptia_viridis_APF46021.1
Eutreptiella_gymnastica_MMETSP0039_CAMPEP_0113747930
Euglena_gracilis_EG_7051_269
Euglena_hiemalis_APF46016.1
Cellvibrionales_bacterium_HAW14001.1
Enhydrobacter_aerosaccus_KND20350.1
Moraxellaceae_bacterium_HBI50201.1
Enhydrobacter_sp._VXB36606.1
Moraxella_osloensis_WP_065264421.1
Gammaproteobacteria_bacterium_NOX77535.1
Pasteurella_testudinis_WP_084256943.1
Shewanella_ulleungensis_GGP92915.1
Photobacterium_piscicola_SKC33910.1
Permianibacter_aggregans_WP_133589177.1
Gammaproteobacteria_bacterium_HBH34870.1
Halobacteria_archaeon_NNG12527.1
Pseudomonas_entomophila_WP_011536328.1
Pseudomonas_monteilii_WP_119368442.1
Pseudomonas_fluorescens_WP_158458800.1
Azotobacter_vinelandii_ACO80865.1
Pseudomonas_massiliensis_WP_040260551.1
Endozoicomonas_arenosclerae_WP_062264474.1
Agaribacterium_haliotis_WP_096086732.1
Oceanospirillaceae_bacterium_HBD23813.1
Chlamydomonas_reinhardtii_XP_001701485.1
Volvox_carteri_XP_002950840.1
Gonium_pectorale_KXZ52405.1
Scenedesmus_sp._KAF6257779.1
Chlorella_variabilis_XP_005843555.1
Trebouxia_sp._KAA6424784.1
Bathycoccus_prasinos_XP_007509251.1
Micromonas_commoda_XP_002507564.1
Carex_littledalei_KAF3322503.1
Asparagus_officinalis_ONK60972.1
Hordeum_vulgare_KAE8804150.1
Gracilariopsis_chorda_PXF48195.1
Porphyra_umbilicalis_OSX71464.1
Galdieria_sulphuraria_XP_005704560.1
Porphyridium_purpureum_KAA8492666.1
Cyanidioschyzon_merolae_XP_005535635.1
Fragilariopsis_cylindrus_OEU23734.1
Thalassiosira_pseudonana_XP_002292986.1
Phaeodactylum_tricornutum_XP_002179459.1
Ectocarpus_siliculosus_CBJ30046.1
Guillardia_theta_ABD51930.1
Vitrella_brassicaformis_CEM32015.1
Chromera_velia_AEQ18820.1
Oxyrrhis_marina_ACE81820.1
0.4

### Fig. S49: UROS

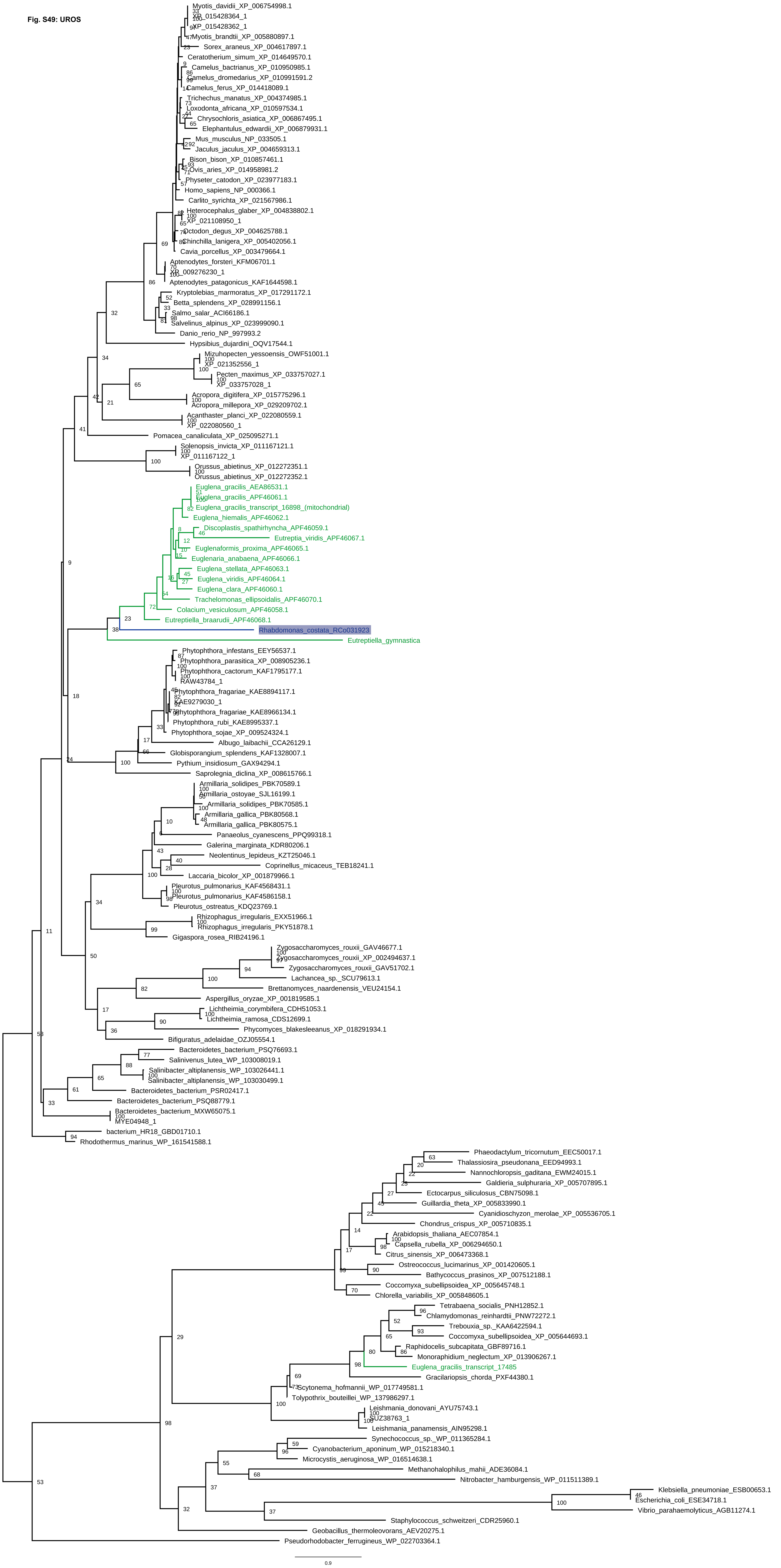

Fig. S50: UROD

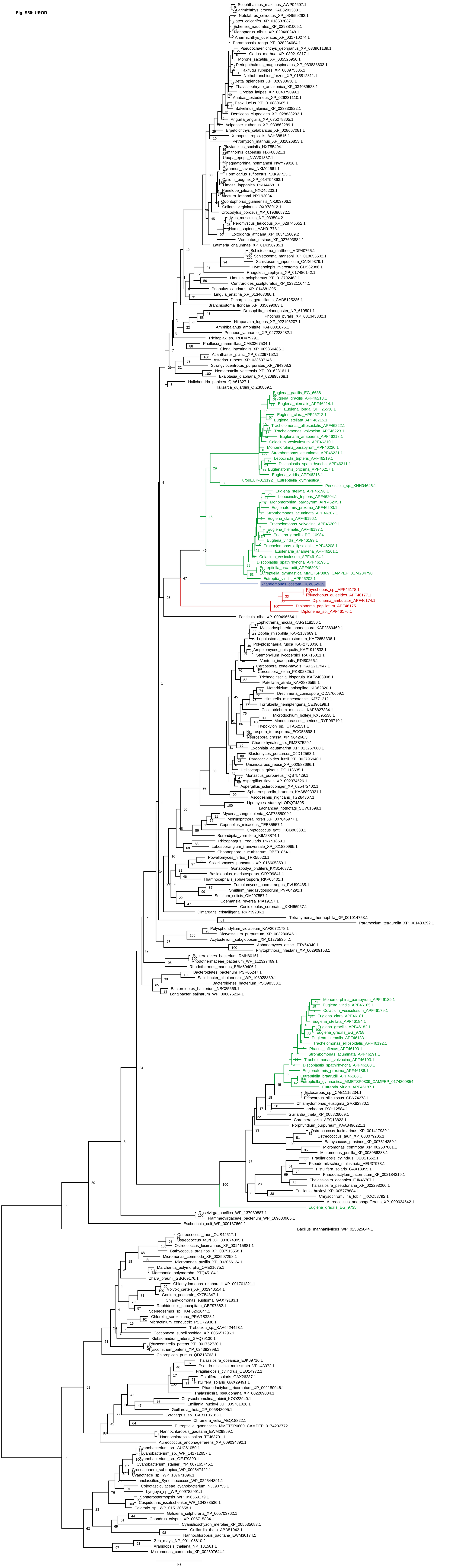

Fig. S51: CPOX

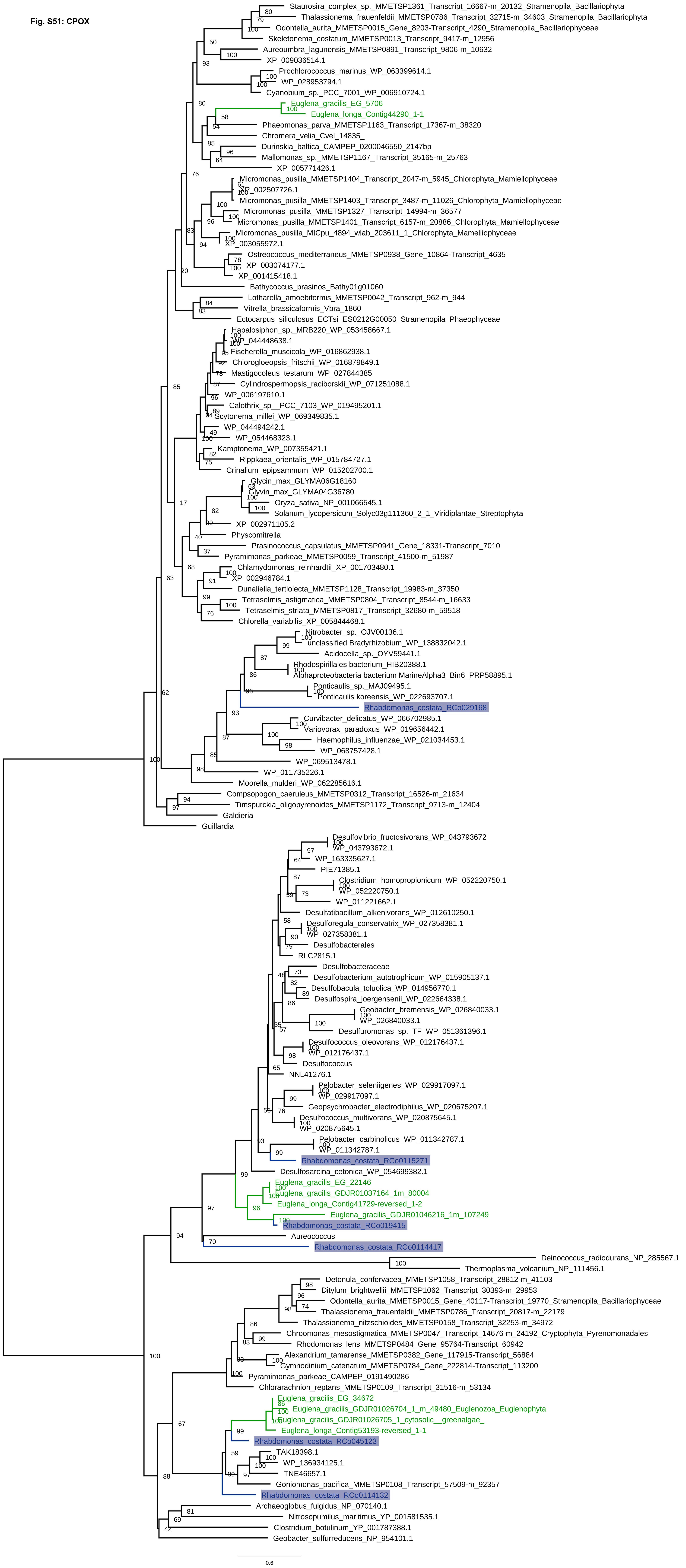

Staurosira_complex_sp._MMETSP1361_Transcript_16667-m_20132_Stramenopila_Bacillariophyta
Thalassionema_frauenfeldii_MMETSP0786_Transcript_32715-m_34603_Stramenopila_Bacillariophyta
Odontella_aurita_MMETSP0015_Gene_8203-Transcript_4290_Stramenopila_Bacillariophyceae
Skeletonema_costatum_MMETSP0013_Transcript_9417-m_12956
Aureoumbra_lagunensis_MMETSP0891_Transcript_9806-m_10632
XP_009036514.1
Prochlorococcus_marinus_WP_063399614.1
WP_028953794.1
Cyanobium_sp._PCC_7001_WP_006910724.1
Euglena_gracilis_EG_5706
Euglena_longa_Contig44290_1-1
Phaeomonas_parva_MMETSP1163_Transcript_17367-m_38320
Chromera_velia_Cvel_14835_
Durinskia_baltica_CAMPEP_0200046550_2147bp
Mallomonas_sp._MMETSP1167_Transcript_35165-m_25763
XP_005771426.1
Micromonas_pusilla_MMETSP1404_Transcript_2047-m_5945_Chlorophyta_Mamiellophyceae
XP_002507726.1
Micromonas_pusilla_MMETSP1403_Transcript_3487-m_11026_Chlorophyta_Mamiellophyceae
Micromonas_pusilla_MMETSP1327_Transcript_14994-m_36577
Micromonas_pusilla_MMETSP1401_Transcript_6157-m_20886_Chlorophyta_Mamiellophyceae
Micromonas_pusilla_MICpu_4894_wlab_203611_1_Chlorophyta_Mamelliophyceae
XP_003055972.1
Ostreococcus_mediterraneus_MMETSP0938_Gene_10864-Transcript_4635
XP_003074177.1
XP_001415418.1
Bathycoccus_prasinos_Bathy01g01060
Lotharella_amoebiformis_MMETSP0042_Transcript_962-m_944
Vitrella_brassicaformis_Vbra_1860
Ectocarpus_siliculosus_ECTsi_ES0212G00050_Stramenopila_Phaeophyceae
Hapalosiphon_sp._MRB220_WP_053458667.1
WP_044448638.1
Fischerella_muscicola_WP_016862938.1
Chlorogloeopsis_fritschii_WP_016879849.1
Mastigocoleus_testarum_WP_027844385
Cylindrospermopsis_raciborskii_WP_071251088.1
WP_006197610.1
Calothrix_sp__PCC_7103_WP_019495201.1
Scytonema_millei_WP_069349835.1
WP_044494242.1
WP_054468323.1
Kamptonema_WP_007355421.1
Rippkaea_orientalis_WP_015784727.1
Crinalium_epipsammum_WP_015202700.1
Glycin_max_GLYMA06G18160
Glyvin_max_GLYMA04G36780
Oryza_sativa_NP_001066545.1
Solanum_lycopersicum_Solyc03g111360_2_1_Viridiplantae_Streptophyta
XP_002971105.2
Physcomitrella
Prasinococcus_capsulatus_MMETSP0941_Gene_18331-Transcript_7010
Pyramimonas_parkeae_MMETSP0059_Transcript_41500-m_51987
Chlamydomonas_reinhardtii_XP_001703480.1
XP_002946784.1
Dunaliella_tertiolecta_MMETSP1128_Transcript_19983-m_37350
Tetraselmis_astigmatica_MMETSP0804_Transcript_8544-m_16633
Tetraselmis_striata_MMETSP0817_Transcript_32680-m_59518
Chlorella_variabilis_XP_005844468.1
Nitrobacter_sp._OJV00136.1
unclassified Bradyrhizobium_WP_138832042.1
Acidocella_sp._OYV59441.1
Rhodospirillales bacterium_HIB20388.1
Alphaproteobacteria bacterium MarineAlpha3_Bin6_PRP58895.1
Ponticaulis_sp._MAJ09495.1
Ponticaulis koreensis_WP_022693707.1
Rhabdomonas_costata_RCo029168
Curvibacter_delicatus_WP_066702985.1
Variovorax_paradoxus_WP_019656442.1
Haemophilus_influenzae_WP_021034453.1
WP_068757428.1
WP_069513478.1
WP_011735226.1
Moorella_mulderi_WP_062285616.1
Compsopogon_caeruleus_MMETSP0312_Transcript_16526-m_21634
Timspurckia_oligopyrenoides_MMETSP1172_Transcript_9713-m_12404
Galdieria
Guillardia
Desulfovibrio_fructosivorans_WP_043793672
WP_043793672.1
WP_163335627.1
PIE71385.1
Clostridium_homopropionicum_WP_052220750.1
WP_052220750.1
WP_011221662.1
Desulfatibacillum_alkenivorans_WP_012610250.1
Desulforegula_conservatrix_WP_027358381.1
WP_027358381.1
Desulfobacterales
RLC2815.1
Desulfobacteraceae
Desulfobacterium_autotrophicum_WP_015905137.1
Desulfobacula_toluolica_WP_014956770.1
Desulfospira_joergensenii_WP_022664338.1
Geobacter_bremensis_WP_026840033.1
WP_026840033.1
Desulfuromonas_sp._TF_WP_051361396.1
Desulfococcus_oleovorans_WP_012176437.1
WP_012176437.1
Desulfococcus
NNL41276.1
Pelobacter_seleniigenes_WP_029917097.1
WP_029917097.1
Geopsychrobacter_electrodiphilus_WP_020675207.1
Desulfococcus_multivorans_WP_020875645.1
WP_020875645.1
Pelobacter_carbinolicus_WP_011342787.1
WP_011342787.1
Rhabdomonas_costata_RCo0115271
Desulfosarcina_cetonica_WP_054699382.1
Euglena_gracilis_EG_22146
Euglena_gracilis_GDJR01037164_1m_80004
Euglena_longa_Contig41729-reversed_1-2
Euglena_gracilis_GDJR01046216_1m_107249
Rhabdomonas_costata_RCo019415
Aureococcus
Rhabdomonas_costata_RCo0114417
Deinococcus_radiodurans_NP_285567.1
Thermoplasma_volcanium_NP_111456.1
Detonula_confervacea_MMETSP1058_Transcript_28812-m_41103
Ditylum_brightwellii_MMETSP1062_Transcript_30393-m_29953
Odontella_aurita_MMETSP0015_Gene_40117-Transcript_19770_Stramenopila_Bacillariophyceae
Thalassionema_frauenfeldii_MMETSP0786_Transcript_20817-m_22179
Thalassionema_nitzschioides_MMETSP0158_Transcript_32253-m_34972
Chroomonas_mesostigmatica_MMETSP0047_Transcript_14676-m_24192_Cryptophyta_Pyrenomonadales
Rhodomonas_lens_MMETSP0484_Gene_95764-Transcript_60942
Alexandrium_tamarense_MMETSP0382_Gene_117915-Transcript_56884
Gymnodinium_catenatum_MMETSP0784_Gene_222814-Transcript_113200
Pyramimonas_parkeae_CAMPEP_0191490286
Chlorarachnion_reptans_MMETSP0109_Transcript_31516-m_53134
Euglena_gracilis_EG_34672
Euglena_gracilis_GDJR01026704_1_m_49480_Euglenozoa_Euglenophyta
Euglena_gracilis_GDJR01026705_1_cytosolic__greenalgae_
Euglena_longa_Contig53193-reversed_1-1
Rhabdomonas_costata_RCo045123
TAK18398.1
WP_136934125.1
TNE46657.1
Goniomonas_pacifica_MMETSP0108_Transcript_57509-m_92357
Rhabdomonas_costata_RCo0114132
Archaeoglobus_fulgidus_NP_070140.1
Nitrosopumilus_maritimus_YP_001581535.1
Clostridium_botulinum_YP_001787388.1
Geobacter_sulfurreducens_NP_954101.1
0.6

Fig. S52: PPOX

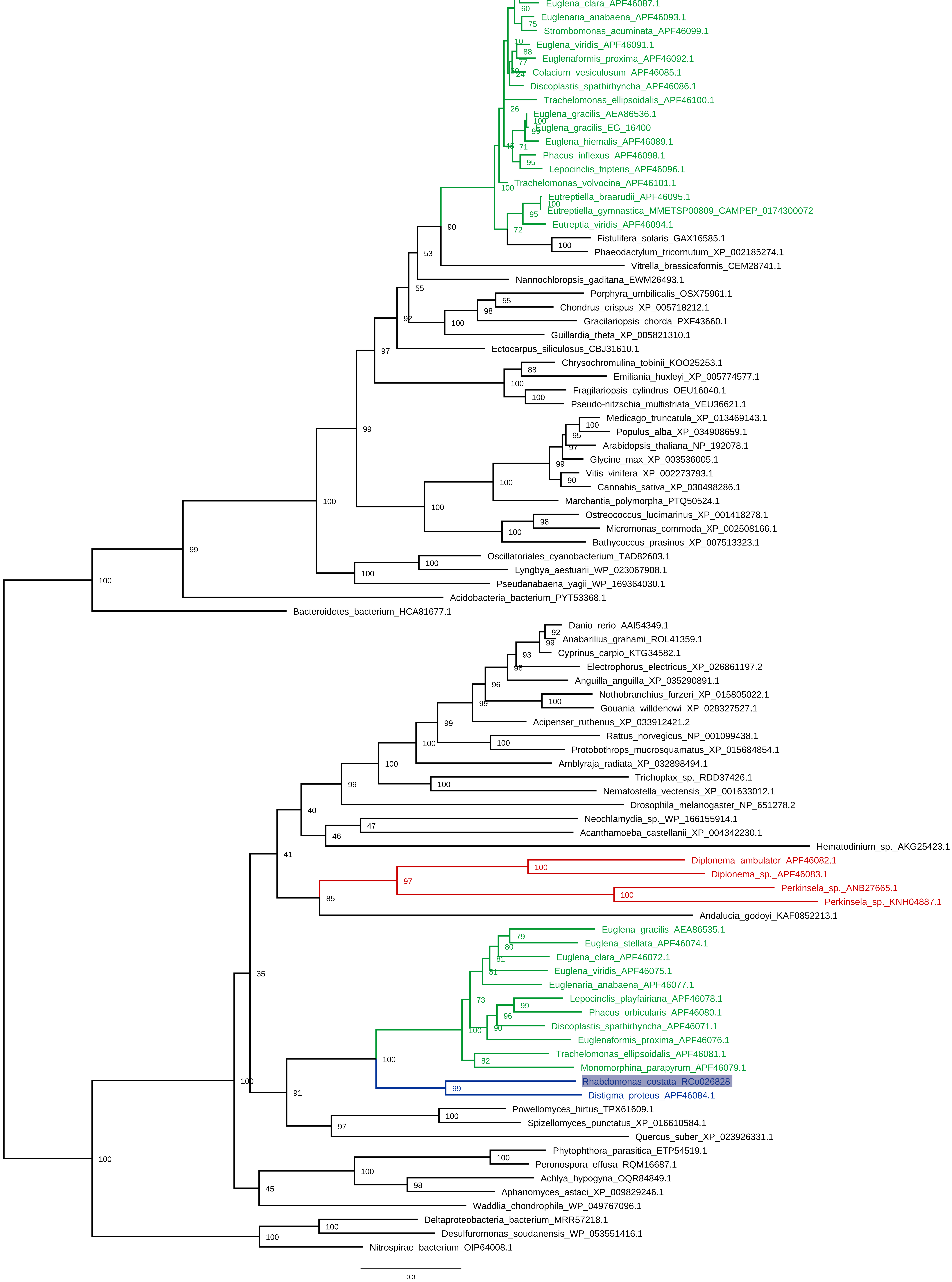
Monomorphina_parapyrum_APF46097.1
Euglena_stellata_APF46090.1
Euglena_clara_APF46087.1
Euglenaria_anabaena_APF46093.1
Strombomonas_acuminata_APF46099.1
Euglena_viridis_APF46091.1
Euglenaformis_proxima_APF46092.1
Colacium_vesiculosum_APF46085.1
Discoplastis_spathirhyncha_APF46086.1
Trachelomonas_ellipsoidalis_APF46100.1
Euglena_gracilis_AEA86536.1
Euglena_gracilis_EG_16400
Euglena_hiemalis_APF46089.1
Phacus_inflexus_APF46098.1
Lepocinclis_tripteris_APF46096.1
Trachelomonas_volvocina_APF46101.1
Eutreptiella_braarudii_APF46095.1
Eutreptiella_gymnastica_MMETSP00809_CAMPEP_0174300072
Eutreptia_viridis_APF46094.1
Fistulifera_solaris_GAX16585.1
Phaeodactylum_tricornutum_XP_002185274.1
Vitrella_brassicaformis_CEM28741.1
Nannochloropsis_gaditana_EWM26493.1
Porphyra_umbilicalis_OSX75961.1
Chondrus_crispus_XP_005718212.1
Gracilariopsis_chorda_PXF43660.1
Guillardia_theta_XP_005821310.1
Ectocarpus_siliculosus_CBJ31610.1
Chrysochromulina_tobinii_KOO25253.1
Emiliania_huxleyi_XP_005774577.1
Fragilariopsis_cylindrus_OEU16040.1
Pseudo-nitzschia_multistriata_VEU36621.1
Medicago_truncatula_XP_013469143.1
Populus_alba_XP_034908659.1
Arabidopsis_thaliana_NP_192078.1
Glycine_max_XP_003536005.1
Vitis_vinifera_XP_002273793.1
Cannabis_sativa_XP_030498286.1
Marchantia_polymorpha_PTQ50524.1
Ostreococcus_lucimarinus_XP_001418278.1
Micromonas_commoda_XP_002508166.1
Bathycoccus_prasinos_XP_007513323.1
Oscillatoriales_cyanobacterium_TAD82603.1
Lyngbya_aestuarii_WP_023067908.1
Pseudanabaena_yagii_WP_169364030.1
Acidobacteria_bacterium_PYT53368.1
Bacteroidetes_bacterium_HCA81677.1
Danio_rerio_AAI54349.1
Anabarilius_grahami_ROL41359.1
Cyprinus_carpio_KTG34582.1
Electrophorus_electricus_XP_026861197.2
Anguilla_anguilla_XP_035290891.1
Nothobranchius_furzeri_XP_015805022.1
Gouania_willdenowi_XP_028327527.1
Acipenser_ruthenus_XP_033912421.2
Rattus_norvegicus_NP_001099438.1
Protobothrops_mucrosquamatus_XP_015684854.1
Amblyraja_radiata_XP_032898494.1
Trichoplax_sp._RDD37426.1
Nematostella_vectensis_XP_001633012.1
Drosophila_melanogaster_NP_651278.2
Neochlamydia_sp._WP_166155914.1
Acanthamoeba_castellanii_XP_004342230.1
Hematodinium_sp._AKG25423.1
Diplonema_ambulator_APF46082.1
Diplonema_sp._APF46083.1
Perkinsela_sp._ANB27665.1
Perkinsela_sp._KNH04887.1
Andalucia_godoyi_KAF0852213.1
Euglena_gracilis_AEA86535.1
Euglena_stellata_APF46074.1
Euglena_clara_APF46072.1
Euglena_viridis_APF46075.1
Euglenaria_anabaena_APF46077.1
Lepocinclis_playfairiana_APF46078.1
Phacus_orbicularis_APF46080.1
Discoplastis_spathirhyncha_APF46071.1
Euglenaformis_proxima_APF46076.1
Trachelomonas_ellipsoidalis_APF46081.1
Monomorphina_parapyrum_APF46079.1
Rhabdomonas_costata_RCo026828
Distigma_proteus_APF46084.1
Powellomyces_hirtus_TPX61609.1
Spizellomyces_punctatus_XP_016610584.1
Quercus_suber_XP_023926331.1
Phytophthora_parasitica_ETP54519.1
Peronospora_effusa_RQM16687.1
Achlya_hypogyna_OQR84849.1
Aphanomyces_astaci_XP_009829246.1
Waddlia_chondrophila_WP_049767096.1
Deltaproteobacteria_bacterium_MRR57218.1
Desulfuromonas_soudanensis_WP_053551416.1
Nitrospirae_bacterium_OIP64008.1
0.3

**Fig. S53: FECH**

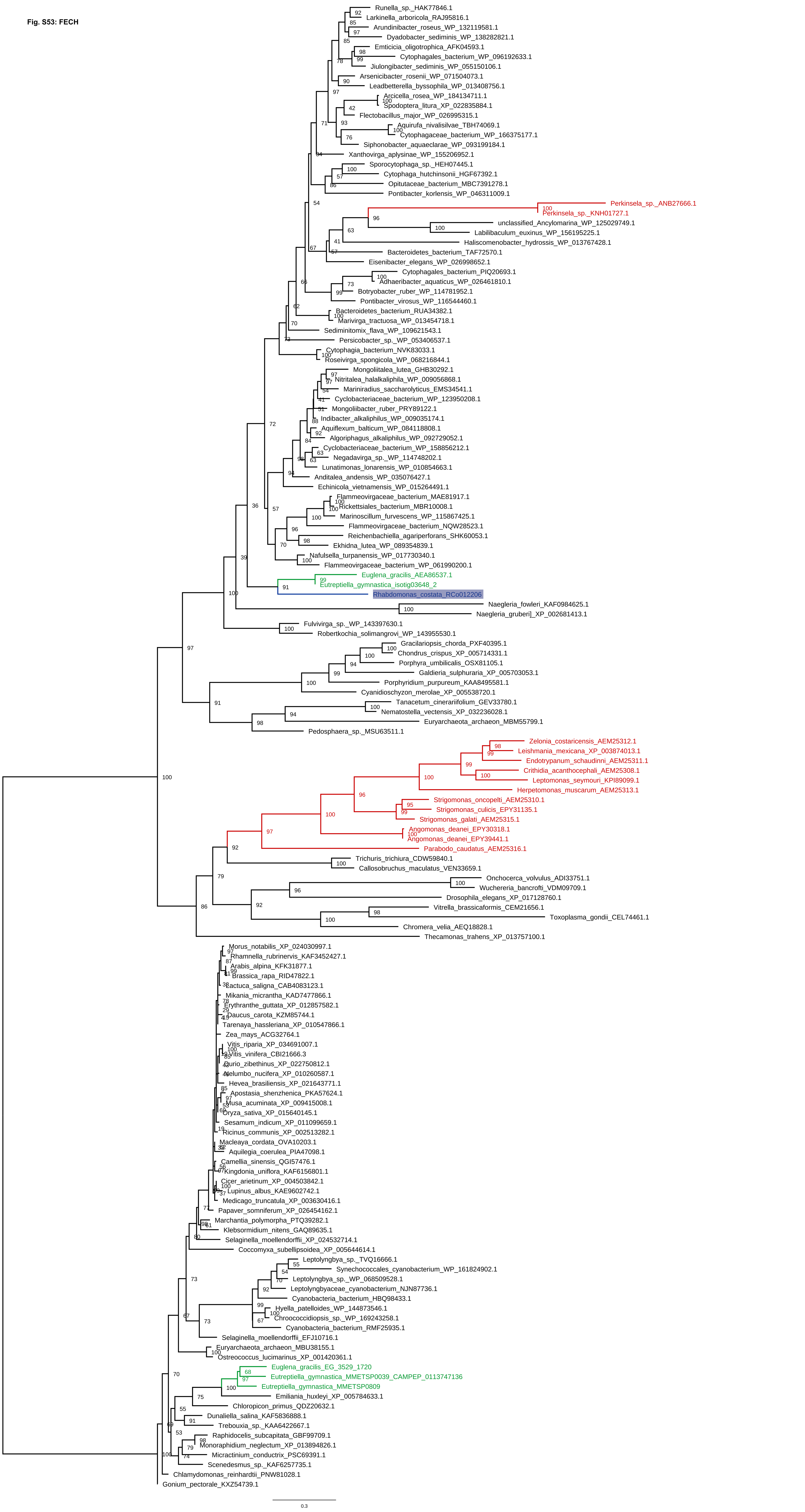

Runella_sp._HAK77846.1
Larkinella_arboricola_RAJ95816.1
Arundinibacter_roseus_WP_132119581.1
Dyadobacter_sediminis_WP_138282821.1
Emticicia_oligotrophica_AFK04593.1
Cytophagales_bacterium_WP_096192633.1
Jiulongibacter_sediminis_WP_055150106.1
Arsenicibacter_rosenii_WP_071504073.1
Leadbetterella_byssophila_WP_013408756.1
Arcicella_rosea_WP_184134711.1
Spodoptera_litura_XP_022835884.1
Flectobacillus_major_WP_026995315.1
Aquirufa_nivalisilvae_TBH74069.1
Cytophagaceae_bacterium_WP_166375177.1
Siphonobacter_aquaeclarae_WP_093199184.1
Xanthovirga_aplysinae_WP_155206952.1
Sporocytophaga_sp._HEH07445.1
Cytophaga_hutchinsonii_HGF67392.1
Opitutaceae_bacterium_MBC7391278.1
Pontibacter_korlensis_WP_046311009.1
Perkinsela_sp._ANB27666.1
Perkinsela_sp._KNH01727.1
unclassified_Ancylomarina_WP_125029749.1
Labilibaculum_euxinus_WP_156195225.1
Haliscomenobacter_hydrossis_WP_013767428.1
Bacteroidetes_bacterium_TAF72570.1
Eisenibacter_elegans_WP_026998652.1
Cytophagales_bacterium_PIQ20693.1
Adhaeribacter_aquaticus_WP_026461810.1
Botryobacter_ruber_WP_114781952.1
Pontibacter_virosus_WP_116544460.1
Bacteroidetes_bacterium_RUA34382.1
Marivirga_tractuosa_WP_013454718.1
Sediminitomix_flava_WP_109621543.1
Persicobacter_sp._WP_053406537.1
Cytophagia_bacterium_NVK83033.1
Roseivirga_spongicola_WP_068216844.1
Mongoliitalea_lutea_GHB30292.1
Nitritalea_halalkaliphila_WP_009056868.1
Mariniradius_saccharolyticus_EMS34541.1
Cyclobacteriaceae_bacterium_WP_123950208.1
Mongoliibacter_ruber_PRY89122.1
Indibacter_alkaliphilus_WP_009035174.1
Aquiflexum_balticum_WP_084118808.1
Algoriphagus_alkaliphilus_WP_092729052.1
Cyclobacteriaceae_bacterium_WP_158856212.1
Negadavirga_sp._WP_114748202.1
Lunatimonas_lonarensis_WP_010854663.1
Anditalea_andensis_WP_035076427.1
Echinicola_vietnamensis_WP_015264491.1
Flammeovirgaceae_bacterium_MAE81917.1
Rickettsiales_bacterium_MBR10008.1
Marinoscillum_furvescens_WP_115867425.1
Flammeovirgaceae_bacterium_NQW28523.1
Reichenbachiella_agariperforans_SHK60053.1
Ekhidna_lutea_WP_089354839.1
Nafulsella_turpanensis_WP_017730340.1
Flammeovirgaceae_bacterium_WP_061990200.1
Euglena_gracilis_AEA86537.1
Eutreptiella_gymnastica_isotig03648_2
Rhabdomonas_costata_RCo012206
Naegleria_fowleri_KAF0984625.1
Naegleria_gruberi]_XP_002681413.1
Fulvivirga_sp._WP_143397630.1
Robertkochia_solimangrovi_WP_143955530.1
Gracilariopsis_chorda_PXF40395.1
Chondrus_crispus_XP_005714331.1
Porphyra_umbilicalis_OSX81105.1
Galdieria_sulphuraria_XP_005703053.1
Porphyridium_purpureum_KAA8495581.1
Cyanidioschyzon_merolae_XP_005538720.1
Tanacetum_cinerariifolium_GEV33780.1
Nematostella_vectensis_XP_032236028.1
Euryarchaeota_archaeon_MBM55799.1
Pedosphaera_sp._MSU63511.1
Zelonia_costaricensis_AEM25312.1
Leishmania_mexicana_XP_003874013.1
Endotrypanum_schaudinni_AEM25311.1
Crithidia_acanthocephali_AEM25308.1
Leptomonas_seymouri_KPI89099.1
Herpetomonas_muscarum_AEM25313.1
Strigomonas_oncopelti_AEM25310.1
Strigomonas_culicis_EPY31135.1
Strigomonas_galati_AEM25315.1
Angomonas_deanei_EPY30318.1
Angomonas_deanei_EPY39441.1
Parabodo_caudatus_AEM25316.1
Trichuris_trichiura_CDW59840.1
Callosobruchus_maculatus_VEN33659.1
Onchocerca_volvulus_ADI33751.1
Wuchereria_bancrofti_VDM09709.1
Drosophila_elegans_XP_017128760.1
Vitrella_brassicaformis_CEM21656.1
Toxoplasma_gondii_CEL74461.1
Chromera_velia_AEQ18828.1
Thecamonas_trahens_XP_013757100.1
Morus_notabilis_XP_024030997.1
Rhamnella_rubrinervis_KAF3452427.1
Arabis_alpina_KFK31877.1
Brassica_rapa_RID47822.1
Lactuca_saligna_CAB4083123.1
Mikania_micrantha_KAD7477866.1
Erythranthe_guttata_XP_012857582.1
Daucus_carota_KZM85744.1
Tarenaya_hassleriana_XP_010547866.1
Zea_mays_ACG32764.1
Vitis_riparia_XP_034691007.1
Vitis_vinifera_CBI21666.3
Durio_zibethinus_XP_022750812.1
Nelumbo_nucifera_XP_010260587.1
Hevea_brasiliensis_XP_021643771.1
Apostasia_shenzhenica_PKA57624.1
Musa_acuminata_XP_009415008.1
Oryza_sativa_XP_015640145.1
Sesamum_indicum_XP_011099659.1
Ricinus_communis_XP_002513282.1
Macleaya_cordata_OVA10203.1
Aquilegia_coerulea_PIA47098.1
Camellia_sinensis_QGI57476.1
Kingdonia_uniflora_KAF6156801.1
Cicer_arietinum_XP_004503842.1
Lupinus_albus_KAE9602742.1
Medicago_truncatula_XP_003630416.1
Papaver_somniferum_XP_026454162.1
Marchantia_polymorpha_PTQ39282.1
Klebsormidium_nitens_GAQ89635.1
Selaginella_moellendorffii_XP_024532714.1
Coccomyxa_subellipsoidea_XP_005644614.1
Leptolyngbya_sp._TVQ16666.1
Synechococcales_cyanobacterium_WP_161824902.1
Leptolyngbya_sp._WP_068509528.1
Leptolyngbyaceae_cyanobacterium_NJN87736.1
Cyanobacteria_bacterium_HBQ98433.1
Hyella_patelloides_WP_144873546.1
Chroococcidiopsis_sp._WP_169243258.1
Cyanobacteria_bacterium_RMF25935.1
Selaginella_moellendorffii_EFJ10716.1
Euryarchaeota_archaeon_MBU38155.1
Ostreococcus_lucimarinus_XP_001420361.1
Euglena_gracilis_EG_3529_1720
Eutreptiella_gymnastica_MMETSP0039_CAMPEP_0113747136
Eutreptiella_gymnastica_MMETSP0809
Emiliania_huxleyi_XP_005784633.1
Chloropicon_primus_QDZ20632.1
Dunaliella_salina_KAF5836888.1
Trebouxia_sp._KAA6422667.1
Raphidocelis_subcapitata_GBF99709.1
Monoraphidium_neglectum_XP_013894826.1
Micractinium_conductrix_PSC69391.1
Scenedesmus_sp._KAF6257735.1
Chlamydomonas_reinhardtii_PNW81028.1
Gonium_pectorale_KXZ54739.1
0.3
