# Supplementary material for: Heterotrophic euglenid Rhabdomonas costata resembles its phototrophic relatives in many aspects of molecular and cell biology
Source: Sci Rep. 2021 Jun 22;11:13070. doi: 10.1038/s41598-021-92174-3 (PMC8219788; doi:10.1038/s41598-021-92174-3)
Supplement: Supplementary file 6 — Supplementary Figures 54-S55. [file 41598_2021_92174_MOESM6_ESM.pdf]

Fig. S54: Histogram of GC content of *Rhabdomonas costata* transcripts with affiliation to eukaryotes (green) and prokaryotes (red). We took advantage of the fact that using phylogenetic pipeline we have selected from the transcriptome sets of 2718 and 441 transcripts with robust eukaryotic and prokaryotic affiliations, respectively. GC content was calculated for the coding sequences of these transcript and compared. Histogram , shows that GC of transcripts with eukaryotic affiliation does not drop below 0.45, unlike those related to prokaryotes which will contain contaminants.

Fig. S55: Histogram of codon adaptation index of *Rhabdomonas costata* transcripts with affiliation to eukaryotes (blue) and prokaryotes (red). We took advantage of the fact that using phylogenetic pipeline we have selected from the transcriptome sets of 2718 and 441 transcripts with robust eukaryotic and prokaryotic affiliations, respectively. CAI (codon adaptation index) for each transcript was evaluated using a reference codon usage table calculated from 2718 transcripts with eukaryotic affiliation. All calculations have been performed on Galaxy platform (usegalagy.org). Histogram shows that CAI of transcripts with eukaryotic affiliation does not drop below 0.45, unlike those related to prokaryotes which will contain contaminants.

Fig. S54

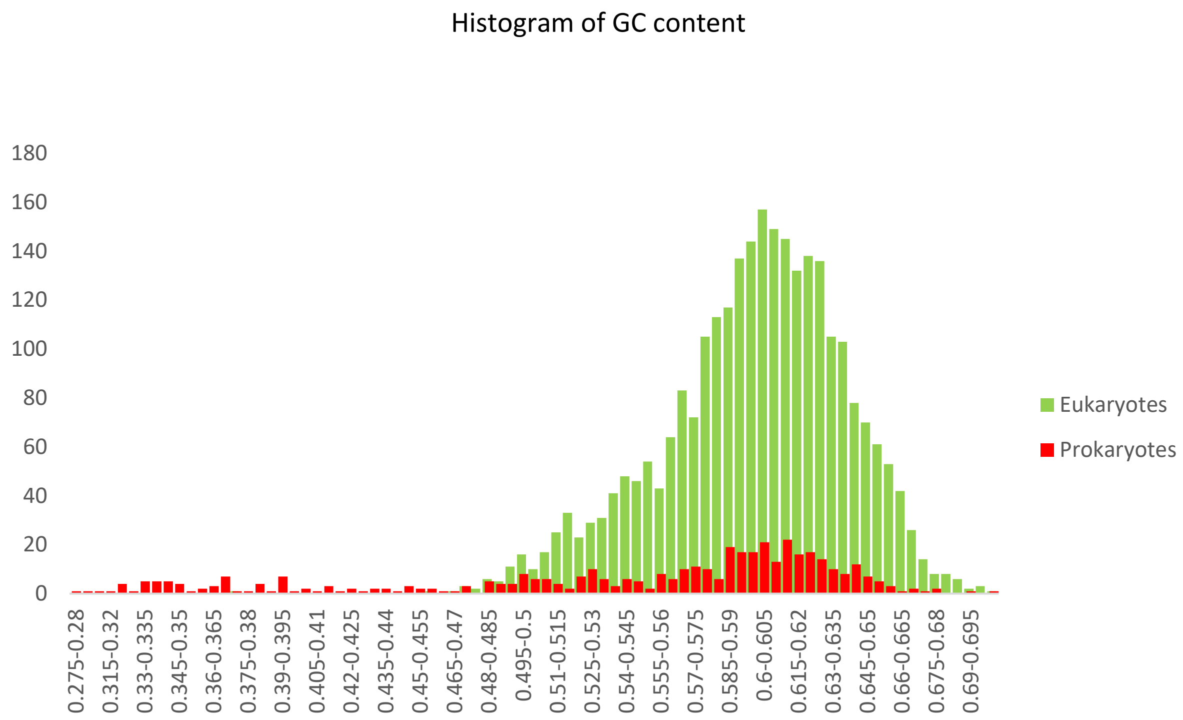

Histogram of GC content
180
160
140
120
100
80
60
40
20
0
0.275-0.28
0.315-0.32
0.33-0.335
0.345-0.35
0.36-0.365
0.375-0.38
0.39-0.395
0.405-0.41
0.42-0.425
0.435-0.44
0.45-0.455
0.465-0.47
0.48-0.485
0.495-0.5
0.51-0.515
0.525-0.53
0.54-0.545
0.555-0.56
0.57-0.575
0.585-0.59
0.6-0.605
0.615-0.62
0.63-0.635
0.645-0.65
0.66-0.665
0.675-0.68
0.69-0.695
Eukaryotes
Prokaryotes

Fig. S55

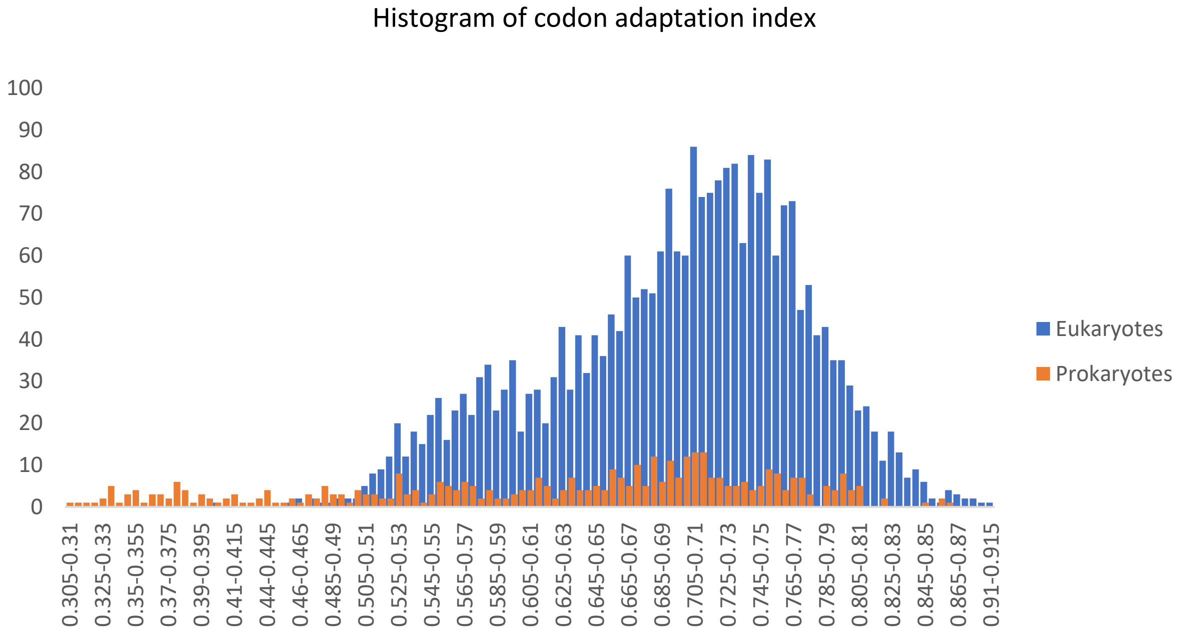
Histogram of codon adaptation index
100
90
80
70
60
50
40
30
20
10
0
0.305-0.31
0.325-0.33
0.35-0.355
0.37-0.375
0.39-0.395
0.41-0.415
0.44-0.445
0.46-0.465
0.485-0.49
0.505-0.51
0.525-0.53
0.545-0.55
0.565-0.57
0.585-0.59
0.605-0.61
0.625-0.63
0.645-0.65
0.665-0.67
0.685-0.69
0.705-0.71
0.725-0.73
0.745-0.75
0.765-0.77
0.785-0.79
0.805-0.81
0.825-0.83
0.845-0.85
0.865-0.87
0.91-0.915
Eukaryotes
Prokaryotes
