# Supplementary material for: Heterotrophic euglenid Rhabdomonas costata resembles its phototrophic relatives in many aspects of molecular and cell biology
Source: Sci Rep. 2021 Jun 22;11:13070. doi: 10.1038/s41598-021-92174-3 (PMC8219788; doi:10.1038/s41598-021-92174-3)
Supplement: Supplementary file 3 — Supplementary Figures S24-S33. [file 41598_2021_92174_MOESM3_ESM.pdf]

Fig. S24: Phylogenetic tree of ester synthases/diacylglycerol acyltransferases (WSD). The tree was constructed in IQ-Tree using the LG+F+R5 model selected by Bayesian information criterion from a trimmed and manually inspected alignment containing 269 amino acid positions. The values at the nodes represent ultrafast bootstraps from 1,000 repetitions. *Euglena gracilis* sequences are shown in green, *Rhabdomonas costata* sequences in blue. The figure was created in FigTree v 1.4.4.

Fig. S25: Phylogenetic tree of mitochondrial carrier family proteins (MCF). The tree was constructed in IQ-Tree using the LG+F+R4 model selected by Bayesian information criterion from a trimmed and manually inspected alignment containing 226 amino acid positions. The values at the nodes represent ultrafast bootstraps from 1,000 repetitions. The figure was created in FigTree v 1.4.4.

Fig. S26: Phylogenetic tree of Hsp70 family proteins (Hsp70). The tree was constructed in IQ-Tree using the LG+I+G4 model selected by Bayesian information criterion from a trimmed and manually inspected alignment containing 439 amino acid positions. The values at the nodes represent ultrafast bootstraps from 1,000 repetitions. The cytosolic, ER and mitochondrial paralogues of Hsp70 are shown in blue, brown and red branches respectively. *Rhabdomonas costata* sequences are indicated by blue names. Based on the position in the tree the transcripts RCo049920 and RCo045932 are considered as mitochondrial. The figure was created in FigTree v 1.4.4.

Fig. S27: Phylogenetic tree of pyruvate dehydrogenase E1 component alpha subunit (PDH).
The tree of *R. costata* and homologues from NCBI and EukProt database (https://www.biorxiv.org/content/10.1101/2020.06.30.180687v1) was constructed in IQ-TREE using the model finder-selected model from a TrimAl-trimmed and manually inspected alignment. The values at the nodes represent ultrafast bootstraps from 1,000 repetitions. Euglenophyte sequences are shown in green, other euglenozoa in red. Please note that the sequence ID numbers starting EP0… are arbitrary and do not correspond to the transcriptome database of respective species. The figure was created in FigTree v 1.4.4.

Fig. S28: Phylogenetic tree of malate synthase part of malate synthase/isocitrate lyase fusion enzyme (MS part).
The tree of *R. costata* and homologues from NCBI and EukProt database (https://www.biorxiv.org/content/10.1101/2020.06.30.180687v1) was constructed in IQ-TREE using the model finder-selected model from a TrimAl-trimmed and manually inspected alignment. The values at the nodes represent ultrafast bootstraps from 1,000 repetitions. Euglenophyte sequences are shown in green, other euglenozoa in red. Please note that the sequence ID numbers starting EP0… are arbitrary and do not correspond to the transcriptome database of respective species. The order of mala synthase and isocitrate lyase parts in taxa with the fusion gene is indicated. The figure was created in FigTree v 1.4.4.

Fig. S29: Phylogenetic tree of isocitrate lyase part of malate synthase/isocitrate lyase fusion enzyme (IL part).
The tree of *R. costata* and homologues from NCBI and EukProt database (https://www.biorxiv.org/content/10.1101/2020.06.30.180687v1) was constructed in IQ-TREE using the model finder-selected model from a TrimAl-trimmed and manually inspected alignment. The values at the nodes represent ultrafast bootstraps from 1,000 repetitions. Euglenophyte sequences are shown in green, other euglenozoa in red. Please note that the sequence ID numbers starting EP0… are arbitrary and do not correspond to the transcriptome database of respective species. The order of mala synthase and isocitrate lyase parts in taxa with the fusion gene is indicated. The figure was created in FigTree v 1.4.4.

Fig. S30: Phylogenetic tree of alternative oxidase (AOX).
The tree of *R. costata* and homologues from NCBI and EukProt database (https://www.biorxiv.org/content/10.1101/2020.06.30.180687v1) was constructed in IQ-TREE using the model finder-selected model from a TrimAl-trimmed and manually inspected alignment. The values at the nodes represent ultrafast bootstraps from 1,000 repetitions. Euglenophyte sequences are shown in green, other euglenozoa in red. Please note that the sequence ID numbers starting EP0… are arbitrary and do not correspond to the transcriptome database of respective species. The figure was created in FigTree v 1.4.4.

Fig. S31: Phylogenetic tree of phosphoadenosine phosphosulfate reductase (cysH).
The tree of *R. costata* and homologues from NCBI and EukProt database (https://www.biorxiv.org/content/10.1101/2020.06.30.180687v1) was constructed in IQ-TREE using the model finder-selected model from a TrimAl-trimmed and manually inspected alignment. The values at the nodes represent ultrafast bootstraps from 1,000 repetitions. Euglenophyte sequences are shown in green, other euglenozoa in red. Please note that the sequence ID numbers starting EP0… are arbitrary and do not correspond to the transcriptome database of respective species. The figure was created in FigTree v 1.4.4.

Fig. S32: Phylogenetic tree of sulfite reductase (NADPH) flavoprotein alpha-component (cysJ).
The tree of *R. costata* and homologues from NCBI and EukProt database (https://www.biorxiv.org/content/10.1101/2020.06.30.180687v1) was constructed in IQ-TREE using the model finder-selected model from a TrimAl-trimmed and manually inspected alignment. The values at the nodes represent ultrafast bootstraps from 1,000 repetitions. Euglenophyte sequences are shown in green, *Rhabdomonas costata* sequences in blue, other euglenozoa in red. Please note that the sequence ID numbers starting EP0… are arbitrary and do not correspond to the transcriptome database of respective species. The figure was created in FigTree v 1.4.4.

Fig. S33: Phylogenetic tree sulfite reductase (NADPH) hemoprotein beta-component (cysI).
The tree of *R. costata* and homologues from NCBI and EukProt database (https://www.biorxiv.org/content/10.1101/2020.06.30.180687v1) was constructed in IQ-TREE using the model finder-selected model from a TrimAl-trimmed and manually inspected alignment. The values at the nodes represent ultrafast bootstraps from 1,000 repetitions. Euglenophyte sequences are shown in green, other euglenozoa in red. Please note that the sequence ID numbers starting EP0… are arbitrary and do not correspond to the transcriptome database of respective species. The figure was created in FigTree v 1.4.4.

Fig. S24: WSD

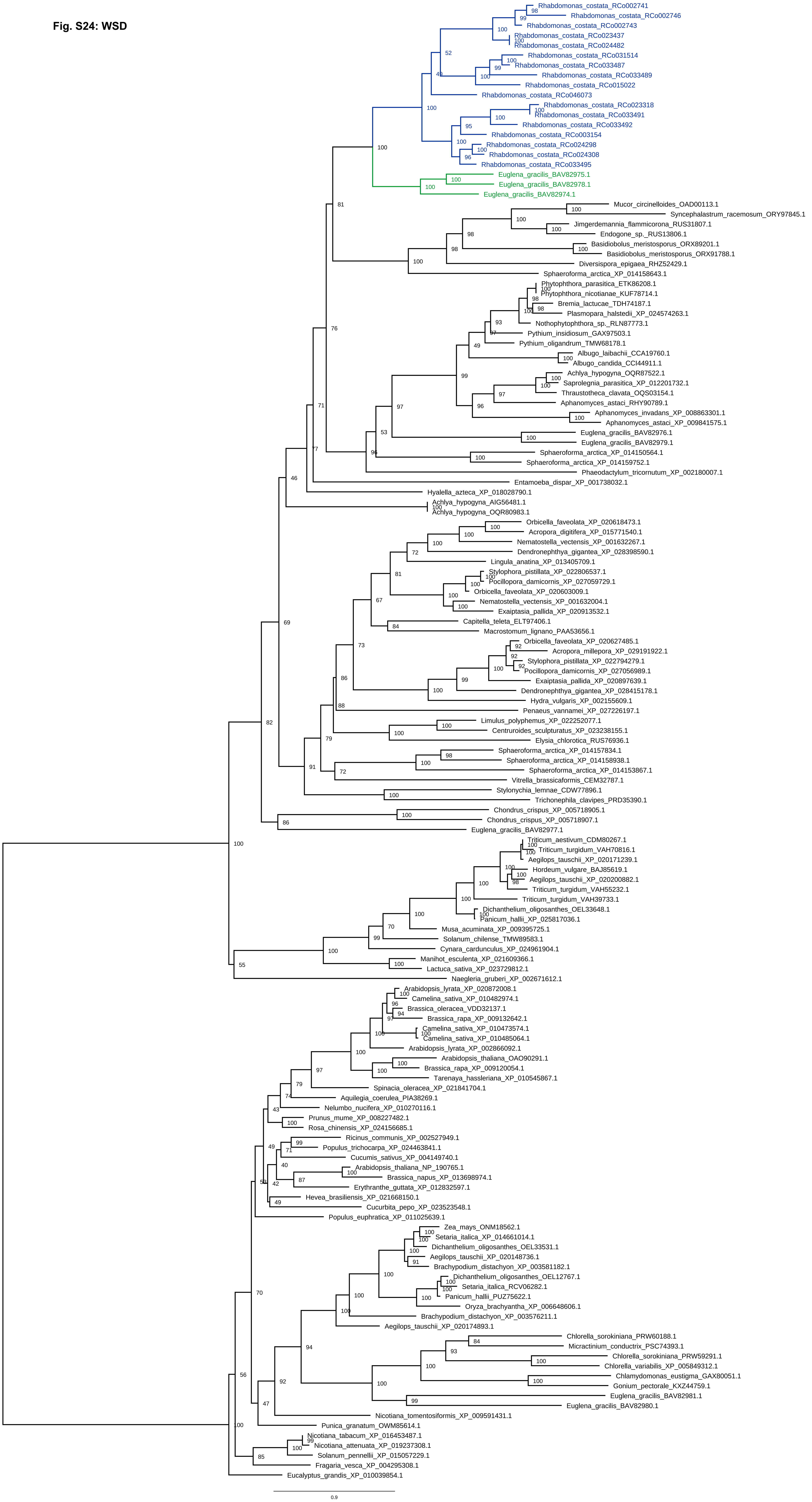
Rhabdomonas_costata_RCo002741
Rhabdomonas_costata_RCo002746
Rhabdomonas_costata_RCo002743
Rhabdomonas_costata_RCo023437
Rhabdomonas_costata_RCo024482
Rhabdomonas_costata_RCo031514
Rhabdomonas_costata_RCo033487
Rhabdomonas_costata_RCo033489
Rhabdomonas_costata_RCo015022
Rhabdomonas_costata_RCo046073
Rhabdomonas_costata_RCo023318
Rhabdomonas_costata_RCo033491
Rhabdomonas_costata_RCo033492
Rhabdomonas_costata_RCo003154
Rhabdomonas_costata_RCo024298
Rhabdomonas_costata_RCo024308
Rhabdomonas_costata_RCo033495
Euglena_gracilis_BAV82975.1
Euglena_gracilis_BAV82978.1
Euglena_gracilis_BAV82974.1
Mucor_circinelloides_OAD00113.1
Syncephalastrum_racemosum_ORY97845.1
Jimgerdemannia_flammicorona_RUS31807.1
Endogone_sp._RUS13806.1
Basidiobolus_meristosporus_ORX89201.1
Basidiobolus_meristosporus_ORX91788.1
Diversispora_epigaea_RHZ52429.1
Sphaeroforma_arctica_XP_014158643.1
Phytophthora_parasitica_ETK86208.1
Phytophthora_nicotianae_KUF78714.1
Bremia_lactucae_TDH74187.1
Plasmopara_halstedii_XP_024574263.1
Nothophytophthora_sp._RLN87773.1
Pythium_insidiosum_GAX97503.1
Pythium_oligandrum_TMW68178.1
Albugo_laibachii_CCA19760.1
Albugo_candida_CCI44911.1
Achlya_hypogyna_OQR87522.1
Saprolegnia_parasitica_XP_012201732.1
Thraustotheca_clavata_OQS03154.1
Aphanomyces_astaci_RHY90789.1
Aphanomyces_invadans_XP_008863301.1
Aphanomyces_astaci_XP_009841575.1
Euglena_gracilis_BAV82976.1
Euglena_gracilis_BAV82979.1
Sphaeroforma_arctica_XP_014150564.1
Sphaeroforma_arctica_XP_014159752.1
Phaeodactylum_tricornutum_XP_002180007.1
Entamoeba_dispar_XP_001738032.1
Hyalella_azteca_XP_018028790.1
Achlya_hypogyna_AIG56481.1
Achlya_hypogyna_OQR80983.1
Orbicella_faveolata_XP_020618473.1
Acropora_digitifera_XP_015771540.1
Nematostella_vectensis_XP_001632267.1
Dendronephthya_gigantea_XP_028398590.1
Lingula_anatina_XP_013405709.1
Stylophora_pistillata_XP_022806537.1
Pocillopora_damicornis_XP_027059729.1
Orbicella_faveolata_XP_020603009.1
Nematostella_vectensis_XP_001632004.1
Exaiptasia_pallida_XP_020913532.1
Capitella_teleta_ELT97406.1
Macrostomum_lignano_PAA53656.1
Orbicella_faveolata_XP_020627485.1
Acropora_millepora_XP_029191922.1
Stylophora_pistillata_XP_022794279.1
Pocillopora_damicornis_XP_027056989.1
Exaiptasia_pallida_XP_020897639.1
Dendronephthya_gigantea_XP_028415178.1
Hydra_vulgaris_XP_002155609.1
Penaeus_vannamei_XP_027226197.1
Limulus_polyphemus_XP_022252077.1
Centruroides_sculpturatus_XP_023238155.1
Elysia_chlorotica_RUS76936.1
Sphaeroforma_arctica_XP_014157834.1
Sphaeroforma_arctica_XP_014158938.1
Sphaeroforma_arctica_XP_014153867.1
Vitrella_brassicaformis_CEM32787.1
Stylonychia_lemnae_CDW77896.1
Trichonephila_clavipes_PRD35390.1
Chondrus_crispus_XP_005718905.1
Chondrus_crispus_XP_005718907.1
Euglena_gracilis_BAV82977.1
Triticum_aestivum_CDM80267.1
Triticum_turgidum_VAH70816.1
Aegilops_tauschii_XP_020171239.1
Hordeum_vulgare_BAJ85619.1
Aegilops_tauschii_XP_020200882.1
Triticum_turgidum_VAH55232.1
Triticum_turgidum_VAH39733.1
Dichanthelium_oligosanthes_OEL33648.1
Panicum_hallii_XP_025817036.1
Musa_acuminata_XP_009395725.1
Solanum_chilense_TMW89583.1
Cynara_cardunculus_XP_024961904.1
Manihot_esculenta_XP_021609366.1
Lactuca_sativa_XP_023729812.1
Naegleria_gruberi_XP_002671612.1
Arabidopsis_lyrata_XP_020872008.1
Camelina_sativa_XP_010482974.1
Brassica_oleracea_VDD32137.1
Brassica_rapa_XP_009132642.1
Camelina_sativa_XP_010473574.1
Camelina_sativa_XP_010485064.1
Arabidopsis_lyrata_XP_002866092.1
Arabidopsis_thaliana_OAO90291.1
Brassica_rapa_XP_009120054.1
Tarenaya_hassleriana_XP_010545867.1
Spinacia_oleracea_XP_021841704.1
Aquilegia_coerulea_PIA38269.1
Nelumbo_nucifera_XP_010270116.1
Prunus_mume_XP_008227482.1
Rosa_chinensis_XP_024156685.1
Ricinus_communis_XP_002527949.1
Populus_trichocarpa_XP_024463841.1
Cucumis_sativus_XP_004149740.1
Arabidopsis_thaliana_NP_190765.1
Brassica_napus_XP_013698974.1
Erythranthe_guttata_XP_012832597.1
Hevea_brasiliensis_XP_021668150.1
Cucurbita_pepo_XP_023523548.1
Populus_euphratica_XP_011025639.1
Zea_mays_ONM18562.1
Setaria_italica_XP_014661014.1
Dichanthelium_oligosanthes_OEL33531.1
Aegilops_tauschii_XP_020148736.1
Brachypodium_distachyon_XP_003581182.1
Dichanthelium_oligosanthes_OEL12767.1
Setaria_italica_RCV06282.1
Panicum_hallii_PUZ75622.1
Oryza_brachyantha_XP_006648606.1
Brachypodium_distachyon_XP_003576211.1
Aegilops_tauschii_XP_020174893.1
Chlorella_sorokiniana_PRW60188.1
Micractinium_conductrix_PSC74393.1
Chlorella_sorokiniana_PRW59291.1
Chlorella_variabilis_XP_005849312.1
Chlamydomonas_eustigma_GAX80051.1
Gonium_pectorale_KXZ44759.1
Euglena_gracilis_BAV82981.1
Euglena_gracilis_BAV82980.1
Nicotiana_tomentosiformis_XP_009591431.1
Punica_granatum_OWM85614.1
Nicotiana_tabacum_XP_016453487.1
Nicotiana_attenuata_XP_019237308.1
Solanum_pennellii_XP_015057229.1
Fragaria_vesca_XP_004295308.1
Eucalyptus_grandis_XP_010039854.1
0.9

Fig. S25: MCF

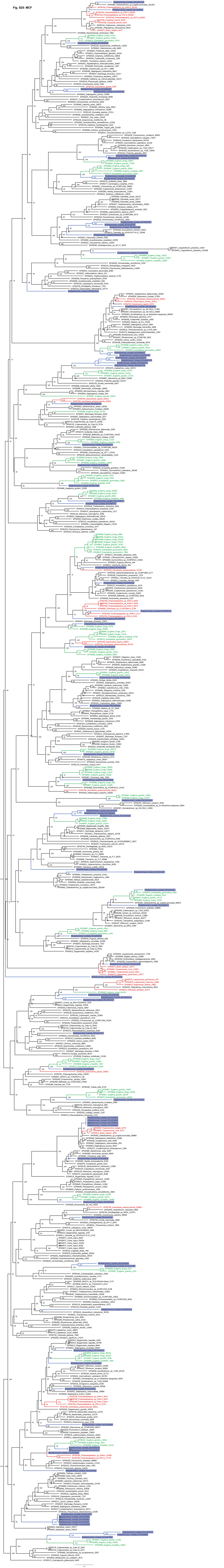

Fig. S26: Hsp70

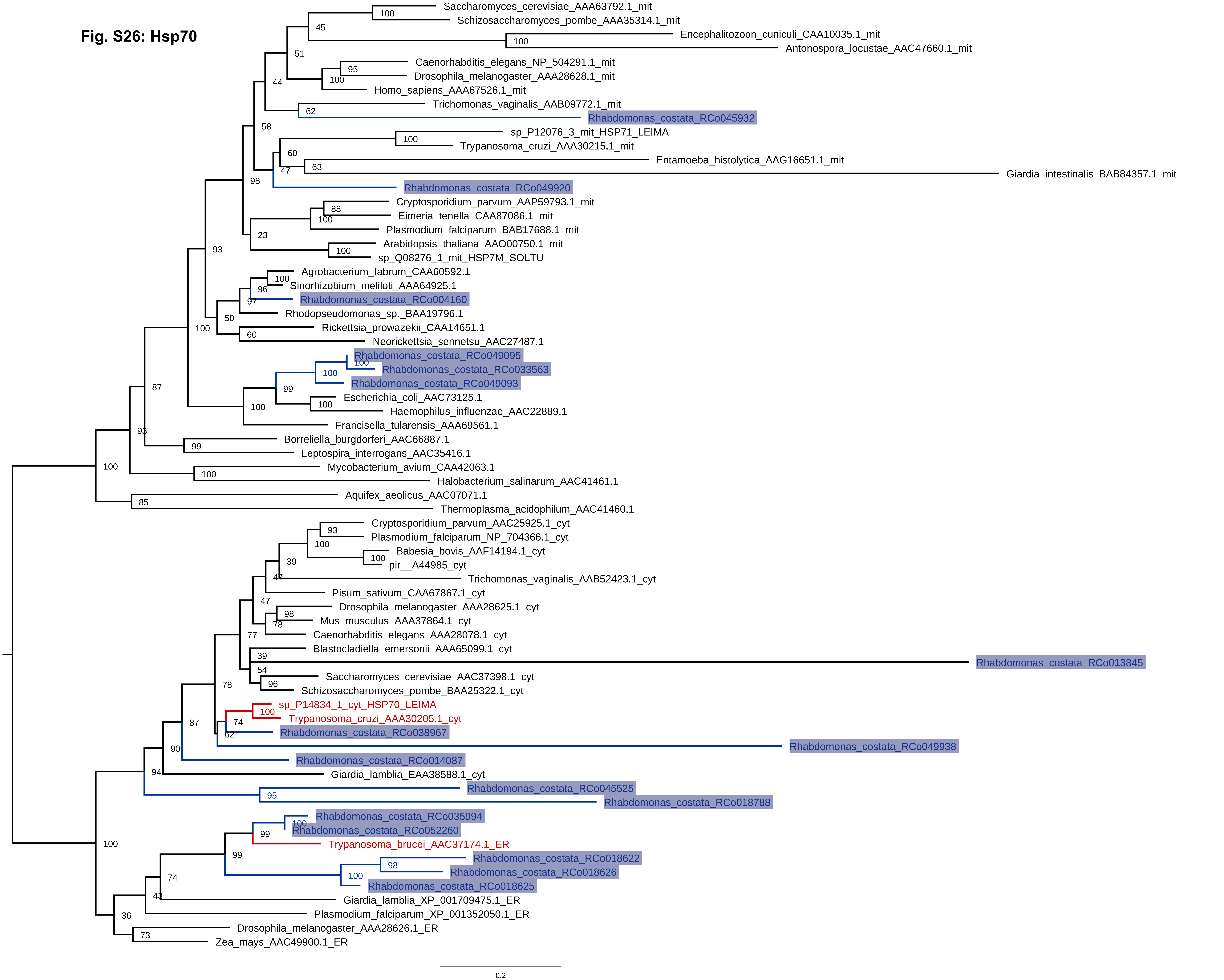

Fig. S27: PDH

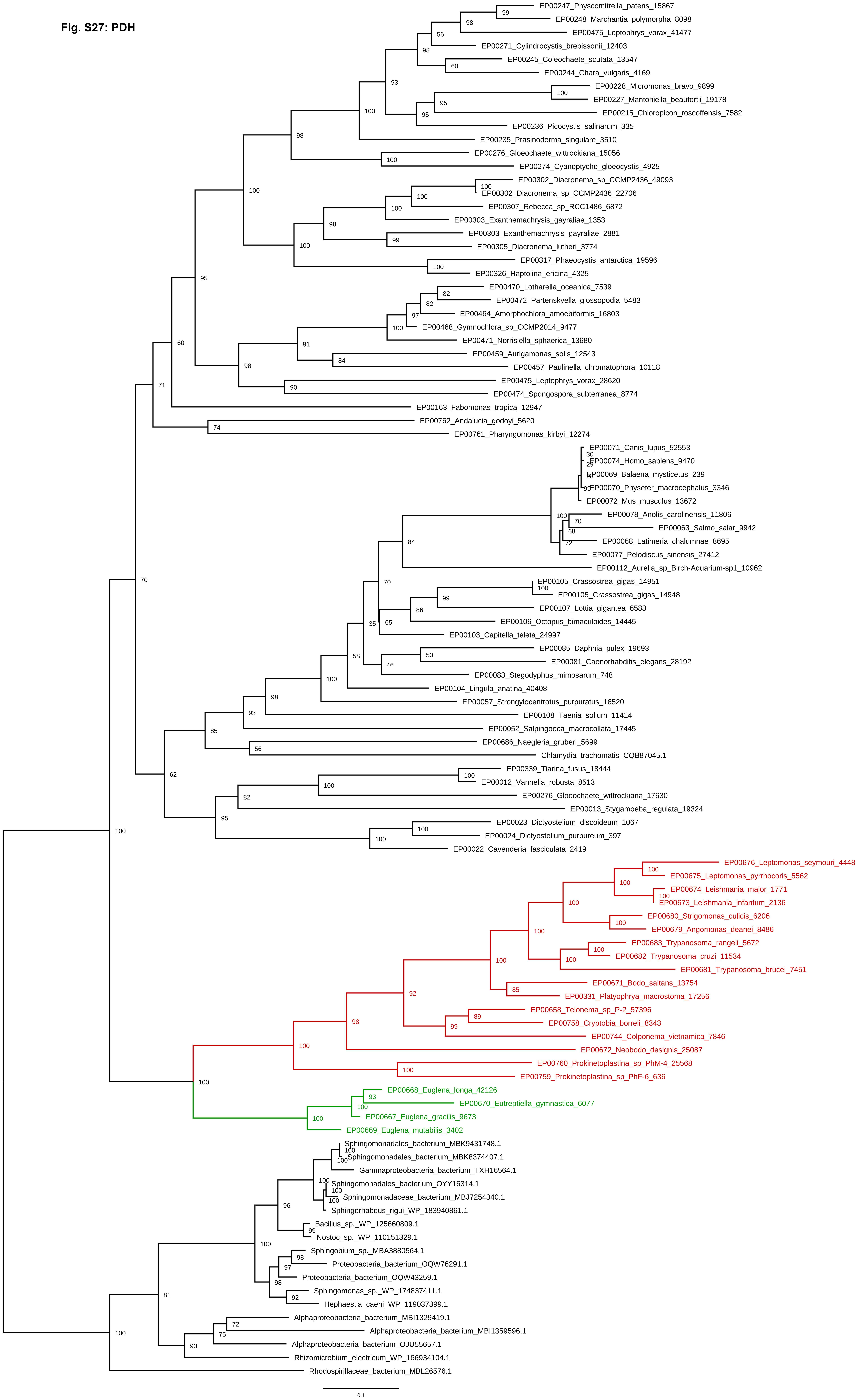
EP00247_Physcomitrella_patens_15867
EP00248_Marchantia_polymorpha_8098
EP00475_Leptophrys_vorax_41477
EP00271_Cylindrocystis_brebissonii_12403
EP00245_Coleochaete_scutata_13547
EP00244_Chara_vulgaris_4169
EP00228_Micromonas_bravo_9899
EP00227_Mantoniella_beaufortii_19178
EP00215_Chloropicon_roscoffensis_7582
EP00236_Picocystis_salinarum_335
EP00235_Prasinoderma_singulare_3510
EP00276_Gloeochaete_wittrockiana_15056
EP00274_Cyanoptyche_gloeocystis_4925
EP00302_Diacronema_sp_CCMP2436_49093
EP00302_Diacronema_sp_CCMP2436_22706
EP00307_Rebecca_sp_RCC1486_6872
EP00303_Exanthemachrysis_gayraliae_1353
EP00303_Exanthemachrysis_gayraliae_2881
EP00305_Diacronema_lutheri_3774
EP00317_Phaeocystis_antarctica_19596
EP00326_Haptolina_ericina_4325
EP00470_Lotharella_oceanica_7539
EP00472_Partenskyella_glossopodia_5483
EP00464_Amorphochlora_amoebiformis_16803
EP00468_Gymnochlora_sp_CCMP2014_9477
EP00471_Norrisiella_sphaerica_13680
EP00459_Aurigamonas_solis_12543
EP00457_Paulinella_chromatophora_10118
EP00475_Leptophrys_vorax_28620
EP00474_Spongospora_subterranea_8774
EP00163_Fabomonas_tropica_12947
EP00762_Andalucia_godoyi_5620
EP00761_Pharyngomonas_kirbyi_12274
EP00071_Canis_lupus_52553
EP00074_Homo_sapiens_9470
EP00069_Balaena_mysticetus_239
EP00070_Physeter_macrocephalus_3346
EP00072_Mus_musculus_13672
EP00078_Anolis_carolinensis_11806
EP00063_Salmo_salar_9942
EP00068_Latimeria_chalumnae_8695
EP00077_Pelodiscus_sinensis_27412
EP00112_Aurelia_sp_Birch-Aquarium-sp1_10962
EP00105_Crassostrea_gigas_14951
EP00105_Crassostrea_gigas_14948
EP00107_Lottia_gigantea_6583
EP00106_Octopus_bimaculoides_14445
EP00103_Capitella_teleta_24997
EP00085_Daphnia_pulex_19693
EP00081_Caenorhabditis_elegans_28192
EP00083_Stegodyphus_mimosarum_748
EP00104_Lingula_anatina_40408
EP00057_Strongylocentrotus_purpuratus_16520
EP00108_Taenia_solium_11414
EP00052_Salpingoeca_macrocollata_17445
EP00686_Naegleria_gruberi_5699
Chlamydia_trachomatis_CQB87045.1
EP00339_Tiarina_fusus_18444
EP00012_Vannella_robusta_8513
EP00276_Gloeochaete_wittrockiana_17630
EP00013_Stygamoeba_regulata_19324
EP00023_Dictyostelium_discoideum_1067
EP00024_Dictyostelium_purpureum_397
EP00022_Cavenderia_fasciculata_2419
EP00676_Leptomonas_seymouri_4448
EP00675_Leptomonas_pyrrhocoris_5562
EP00674_Leishmania_major_1771
EP00673_Leishmania_infantum_2136
EP00680_Strigomonas_culicis_6206
EP00679_Angomonas_deanei_8486
EP00683_Trypanosoma_rangeli_5672
EP00682_Trypanosoma_cruzi_11534
EP00681_Trypanosoma_brucei_7451
EP00671_Bodo_saltans_13754
EP00331_Platyophrya_macrostoma_17256
EP00658_Telonema_sp_P-2_57396
EP00758_Cryptobia_borreli_8343
EP00744_Colponema_vietnamica_7846
EP00672_Neobodo_designis_25087
EP00760_Prokinetoplastina_sp_PhM-4_25568
EP00759_Prokinetoplastina_sp_PhF-6_636
EP00668_Euglena_longa_42126
EP00670_Eutreptiella_gymnastica_6077
EP00667_Euglena_gracilis_9673
EP00669_Euglena_mutabilis_3402
Sphingomonadales_bacterium_MBK9431748.1
Sphingomonadales_bacterium_MBK8374407.1
Gammaproteobacteria_bacterium_TXH16564.1
Sphingomonadales_bacterium_OYY16314.1
Sphingomonadaceae_bacterium_MBJ7254340.1
Sphingorhabdus_rigui_WP_183940861.1
Bacillus_sp._WP_125660809.1
Nostoc_sp._WP_110151329.1
Sphingobium_sp._MBA3880564.1
Proteobacteria_bacterium_OQW76291.1
Proteobacteria_bacterium_OQW43259.1
Sphingomonas_sp._WP_174837411.1
Hephaestia_caeni_WP_119037399.1
Alphaproteobacteria_bacterium_MBI1329419.1
Alphaproteobacteria_bacterium_MBI1359596.1
Alphaproteobacteria_bacterium_OJU55657.1
Rhizomicrobium_electricum_WP_166934104.1
Rhodospirillaceae_bacterium_MBL26576.1
0.1

Fig. S28: MS part

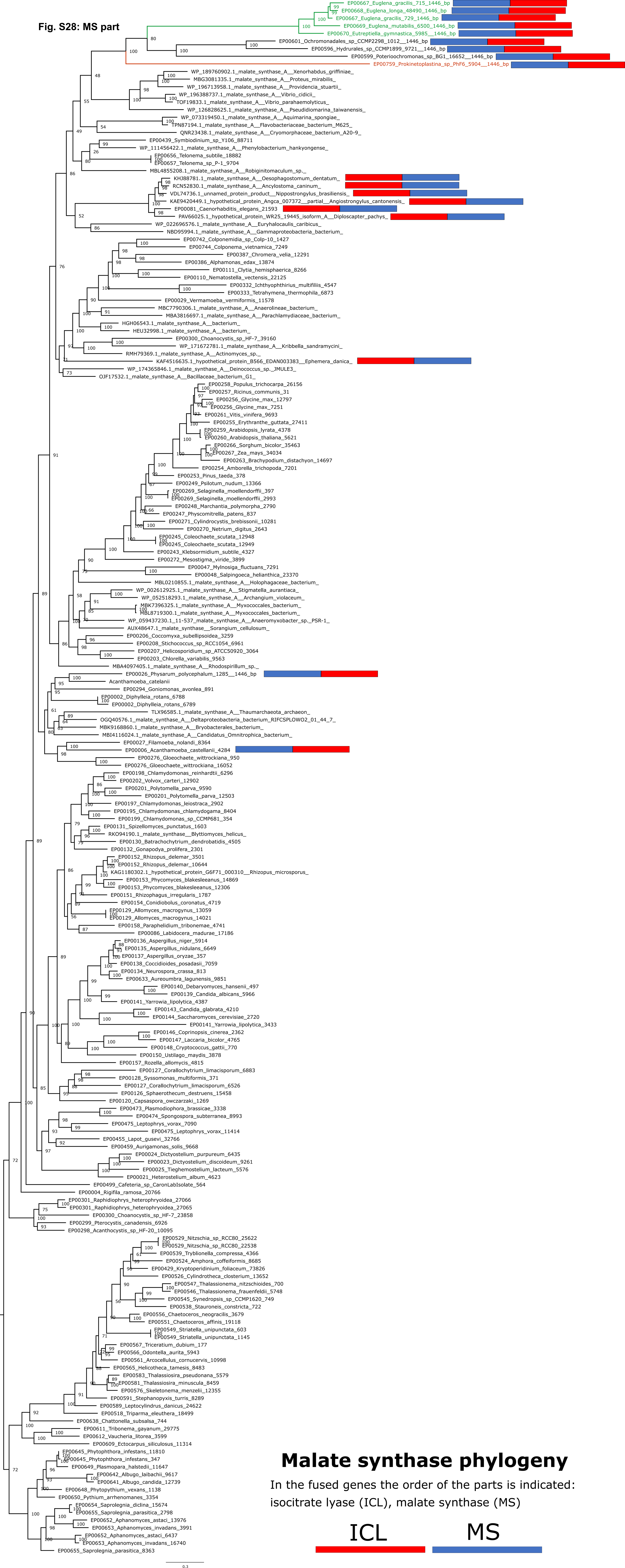

Fig. S29: IL part

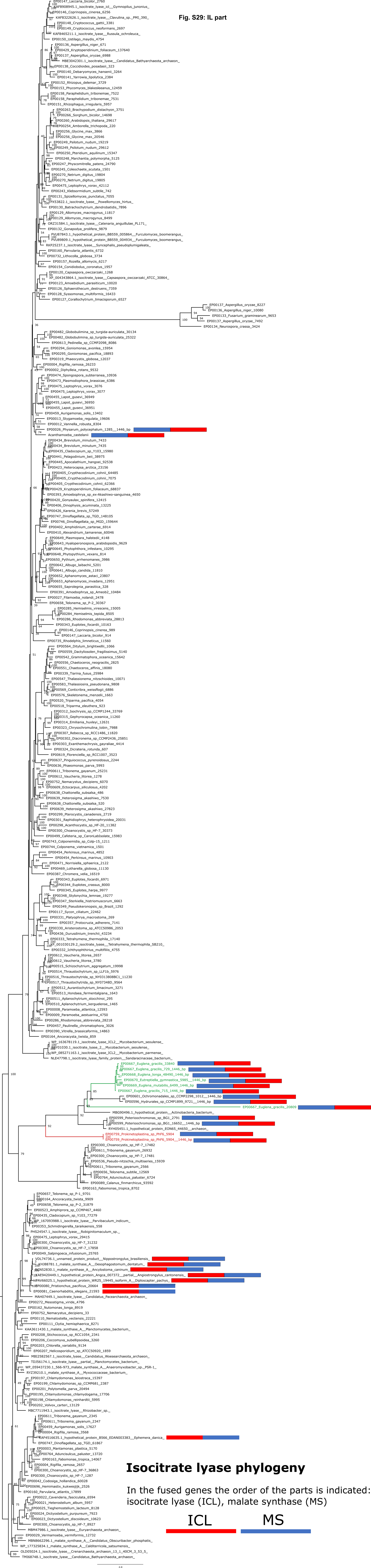

Isocitrate lyase phylogeny

In the fused genes the order of the parts is indicated: isocitrate lyase (ICL), malate synthase (MS)

Fig. S30: AOX

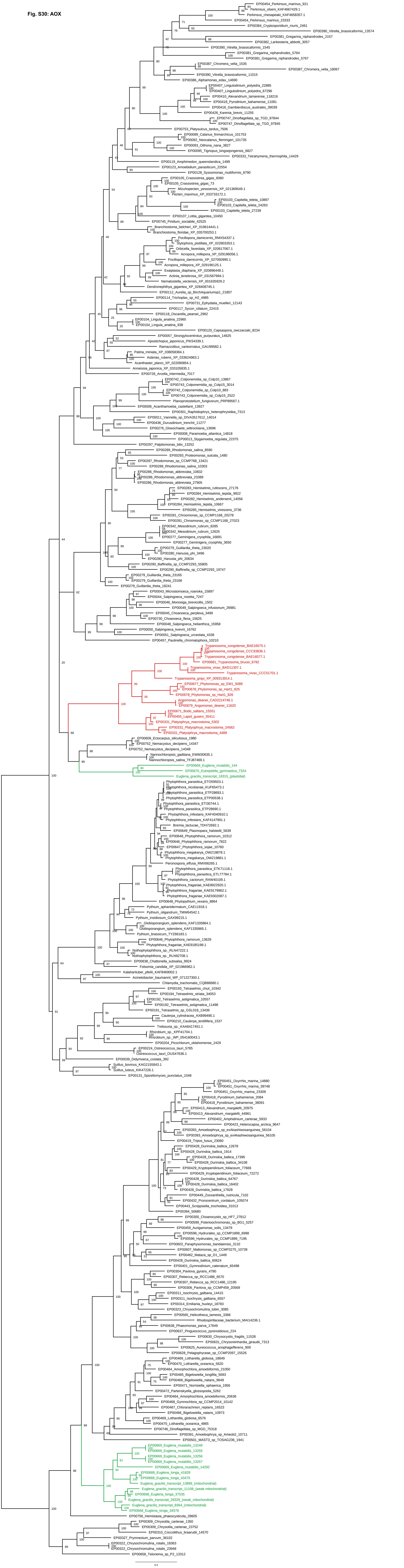

Fig. S31: cysH

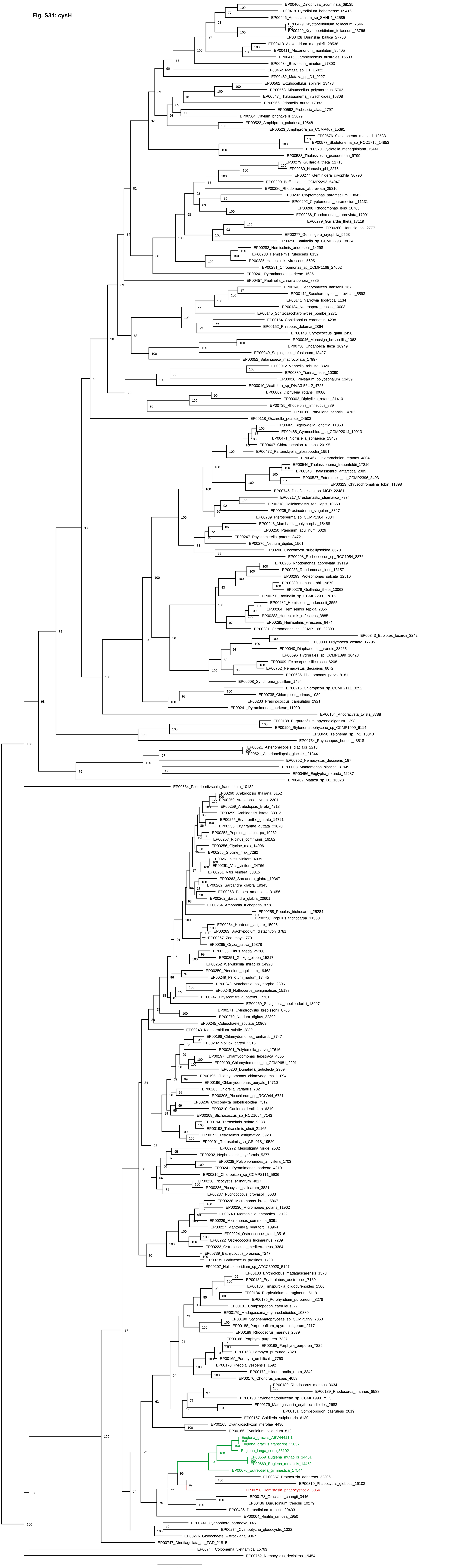
EP00406_Dinophysis_acuminata_68135
EP00418_Pyrodinium_bahamense_65416
EP00446_Apocalathium_sp_SHHI-4_32585
EP00429_Kryptoperidinium_foliaceum_7546
EP00429_Kryptoperidinium_foliaceum_23766
EP00428_Durinskia_baltica_27760
EP00413_Alexandrium_margalefii_28538
EP00411_Alexandrium_monilatum_96405
EP00416_Gambierdiscus_australes_16683
EP00434_Breviolum_minutum_27803
EP00462_Mataza_sp_D1_16022
EP00462_Mataza_sp_D1_9227
EP00562_Extubocellulus_spinifer_13478
EP00563_Minutocellus_polymorphus_5703
EP00547_Thalassionema_nitzschioides_10308
EP00566_Odontella_aurita_17982
EP00592_Proboscia_alata_2797
EP00564_Ditylum_brightwellii_13629
EP00522_Amphiprora_paludosa_10548
EP00523_Amphiprora_sp_CCMP467_15391
EP00576_Skeletonema_menzelii_12588
EP00577_Skeletonema_sp_RCC1716_14853
EP00570_Cyclotella_meneghiniana_15441
EP00583_Thalassiosira_pseudonana_9799
EP00279_Guillardia_theta_11713
EP00280_Hanusia_phi_2275
EP00277_Geminigera_cryophila_30790
EP00290_Baffinella_sp_CCMP2293_54047
EP00286_Rhodomonas_abbreviata_25310
EP00292_Cryptomonas_paramecium_13843
EP00292_Cryptomonas_paramecium_11131
EP00288_Rhodomonas_lens_16763
EP00286_Rhodomonas_abbreviata_17001
EP00279_Guillardia_theta_13119
EP00280_Hanusia_phi_2777
EP00277_Geminigera_cryophila_9563
EP00290_Baffinella_sp_CCMP2293_18634
EP00282_Hemiselmis_andersenii_14298
EP00283_Hemiselmis_rufescens_8132
EP00285_Hemiselmis_virescens_5695
EP00281_Chroomonas_sp_CCMP1168_24002
EP00241_Pyramimonas_parkeae_1686
EP00457_Paulinella_chromatophora_8885
EP00140_Debaryomyces_hansenii_167
EP00144_Saccharomyces_cerevisiae_5593
EP00141_Yarrowia_lipolytica_1134
EP00134_Neurospora_crassa_10003
EP00145_Schizosaccharomyces_pombe_2271
EP00154_Conidiobolus_coronatus_4238
EP00152_Rhizopus_delemar_2864
EP00148_Cryptococcus_gattii_2490
EP00046_Monosiga_brevicollis_1063
EP00730_Choanoeca_flexa_16949
EP00049_Salpingoeca_infusionum_18427
EP00052_Salpingoeca_macrocollata_17997
EP00012_Vannella_robusta_8320
EP00339_Tiarina_fusus_10390
EP00026_Physarum_polycephalum_11459
EP00010_Vexillifera_sp_DIVA3-564-2_4725
EP00002_Diphylleia_rotans_40086
EP00002_Diphylleia_rotans_31410
EP00735_Rhodelphis_limneticus_889
EP00160_Parvularia_atlantis_14703
EP00118_Oscarella_pearsei_24503
EP00465_Bigelowiella_longifila_11863
EP00468_Gymnochlora_sp_CCMP2014_10913
EP00471_Norrisiella_sphaerica_13437
EP00467_Chlorarachnion_reptans_20195
EP00472_Partenskyella_glossopodia_1951
EP00467_Chlorarachnion_reptans_4804
EP00546_Thalassionema_frauenfeldii_17216
EP00548_Thalassiothrix_antarctica_2089
EP00527_Entomoneis_sp_CCMP2396_8493
EP00323_Chrysochromulina_tobin_11898
EP00746_Dinoflagellata_sp_MGD_22481
EP00217_Crustomastix_stigmatica_7374
EP00218_Dolichomastix_tenuilepis_10560
EP00235_Prasinoderma_singulare_3327
EP00239_Pterosperma_sp_CCMP1384_7884
EP00248_Marchantia_polymorpha_15488
EP00250_Pteridium_aquilinum_6029
EP00247_Physcomitrella_patens_34721
EP00270_Netrium_digitus_1561
EP00206_Coccomyxa_subellipsoidea_8870
EP00208_Stichococcus_sp_RCC1054_8876
EP00286_Rhodomonas_abbreviata_19119
EP00288_Rhodomonas_lens_13157
EP00293_Proteomonas_sulcata_12510
EP00280_Hanusia_phi_19870
EP00279_Guillardia_theta_13063
EP00290_Baffinella_sp_CCMP2293_17815
EP00282_Hemiselmis_andersenii_3555
EP00284_Hemiselmis_tepida_2856
EP00283_Hemiselmis_rufescens_3885
EP00285_Hemiselmis_virescens_9474
EP00281_Chroomonas_sp_CCMP1168_22890
EP00343_Euplotes_focardii_3242
EP00039_Didymoeca_costata_17795
EP00040_Diaphanoeca_grandis_38265
EP00596_Hydrurales_sp_CCMP1899_10423
EP00609_Ectocarpus_siliculosus_6208
EP00752_Nemacystus_decipiens_6672
EP00636_Phaeomonas_parva_8181
EP00608_Synchroma_pusillum_1494
EP00216_Chloropicon_sp_CCMP2111_3292
EP00738_Chloropicon_primus_1089
EP00233_Prasinococcus_capsulatus_2921
EP00241_Pyramimonas_parkeae_11020
EP00164_Ancoracysta_twista_8788
EP00188_Purpureofilum_apyrenoidigerum_1398
EP00190_Stylonematophyceae_sp_CCMP1999_6114
EP00658_Telonema_sp_P-2_10040
EP00754_Rhynchopus_humris_43518
EP00521_Asterionellopsis_glacialis_2218
EP00521_Asterionellopsis_glacialis_21344
EP00752_Nemacystus_decipiens_197
EP00003_Mantamonas_plastica_31949
EP00456_Euglypha_rotunda_42287
EP00462_Mataza_sp_D1_16023
EP00534_Pseudo-nitzschia_fraudulenta_10132
EP00260_Arabidopsis_thaliana_6152
EP00259_Arabidopsis_lyrata_2201
EP00259_Arabidopsis_lyrata_4213
EP00259_Arabidopsis_lyrata_38312
EP00255_Erythranthe_guttata_14721
EP00255_Erythranthe_guttata_21870
EP00258_Populus_trichocarpa_19232
EP00257_Ricinus_communis_16182
EP00256_Glycine_max_14996
EP00256_Glycine_max_7282
EP00261_Vitis_vinifera_4039
EP00261_Vitis_vinifera_24766
EP00261_Vitis_vinifera_33015
EP00262_Sarcandra_glabra_19347
EP00262_Sarcandra_glabra_19345
EP00268_Persea_americana_31056
EP00262_Sarcandra_glabra_20601
EP00254_Amborella_trichopoda_8738
EP00258_Populus_trichocarpa_25284
EP00258_Populus_trichocarpa_11550
EP00264_Hordeum_vulgare_15025
EP00263_Brachypodium_distachyon_3781
EP00267_Zea_mays_773
EP00265_Oryza_sativa_15878
EP00253_Pinus_taeda_25380
EP00251_Ginkgo_biloba_15317
EP00252_Welwitschia_mirabilis_14928
EP00250_Pteridium_aquilinum_19468
EP00249_Psilotum_nudum_17445
EP00248_Marchantia_polymorpha_2805
EP00246_Nothoceros_aenigmaticus_15188
EP00247_Physcomitrella_patens_17701
EP00269_Selaginella_moellendorffii_13907
EP00271_Cylindrocystis_brebissonii_8706
EP00270_Netrium_digitus_22302
EP00245_Coleochaete_scutata_10963
EP00243_Klebsormidium_subtile_2830
EP00198_Chlamydomonas_reinhardtii_7747
EP00202_Volvox_carteri_2315
EP00201_Polytomella_parva_17616
EP00197_Chlamydomonas_leiostraca_4655
EP00199_Chlamydomonas_sp_CCMP681_2201
EP00200_Dunaliella_tertiolecta_2909
EP00195_Chlamydomonas_chlamydogama_11094
EP00196_Chlamydomonas_euryale_14710
EP00203_Chlorella_variabilis_732
EP00205_Picochlorum_sp_RCC944_6781
EP00206_Coccomyxa_subellipsoidea_7312
EP00210_Caulerpa_lentillifera_6319
EP00208_Stichococcus_sp_RCC1054_7143
EP00194_Tetraselmis_striata_9383
EP00193_Tetraselmis_chuii_21165
EP00192_Tetraselmis_astigmatica_3928
EP00191_Tetraselmis_sp_GSL018_19520
EP00272_Mesostigma_viride_2532
EP00232_Nephroselmis_pyriformis_5277
EP00238_Polyblepharides_amylifera_1703
EP00241_Pyramimonas_parkeae_4210
EP00216_Chloropicon_sp_CCMP2111_5936
EP00236_Picocystis_salinarum_4817
EP00236_Picocystis_salinarum_3821
EP00237_Pycnococcus_provasolii_6633
EP00228_Micromonas_bravo_5867
EP00230_Micromonas_polaris_11962
EP00740_Mantoniella_antarctica_13122
EP00229_Micromonas_commoda_6391
EP00227_Mantoniella_beaufortii_10964
EP00224_Ostreococcus_tauri_3516
EP00222_Ostreococcus_lucimarinus_7289
EP00223_Ostreococcus_mediterraneus_3384
EP00739_Bathycoccus_prasinos_7247
EP00739_Bathycoccus_prasinos_1790
EP00207_Helicosporidium_sp_ATCC50920_5197
EP00183_Erythrolobus_madagascarensis_1378
EP00182_Erythrolobus_australicus_7180
EP00186_Timspurckia_oligopyrenoides_1506
EP00184_Porphyridium_aerugineum_5119
EP00185_Porphyridium_purpureum_8278
EP00181_Compsopogon_caeruleus_72
EP00179_Madagascaria_erythrocladioides_10380
EP00190_Stylonematophyceae_sp_CCMP1999_7060
EP00188_Purpureofilum_apyrenoidigerum_2717
EP00189_Rhodosorus_marinus_2679
EP00168_Porphyra_purpurea_7327
EP00168_Porphyra_purpurea_7329
EP00168_Porphyra_purpurea_7328
EP00169_Porphyra_umbilicalis_7760
EP00170_Pyropia_yezoensis_1592
EP00172_Hildenbrandia_rubra_3349
EP00176_Chondrus_crispus_4053
EP00189_Rhodosorus_marinus_3634
EP00189_Rhodosorus_marinus_8588
EP00190_Stylonematophyceae_sp_CCMP1999_7525
EP00179_Madagascaria_erythrocladioides_2683
EP00181_Compsopogon_caeruleus_2019
EP00167_Galdieria_sulphuraria_6130
EP00165_Cyanidioschyzon_merolae_4430
EP00166_Cyanidium_caldarium_812
Euglena_gracilis_ABV44411.1
Euglena_gracilis_transcript_13057
Euglena_longa_contig36192
EP00669_Euglena_mutabilis_14451
EP00669_Euglena_mutabilis_14452
EP00670_Eutreptiella_gymnastica_17544
EP00357_Protocruzia_adherens_32306
EP00319_Phaeocystis_globosa_16103
EP00756_Hemistasia_phaeocysticola_3054
EP00178_Gracilaria_changii_3446
EP00436_Durusdinium_trenchii_10279
EP00436_Durusdinium_trenchii_20433
EP00004_Rigifilia_ramosa_2950
EP00741_Cyanophora_paradoxa_146
EP00274_Cyanoptyche_gloeocystis_1332
EP00276_Gloeochaete_wittrockiana_9367
EP00747_Dinoflagellata_sp_TGD_21815
EP00744_Colponema_vietnamica_15763
EP00752_Nemacystus_decipiens_19454
0.4

Fig. S32: cysJ

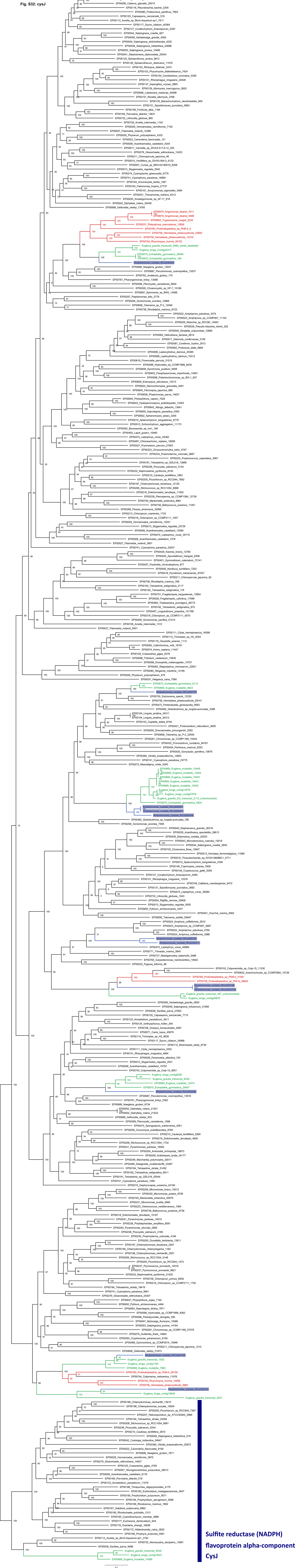
Sulfite reductase (NADPH)
flavoprotein alpha-component
CysJ

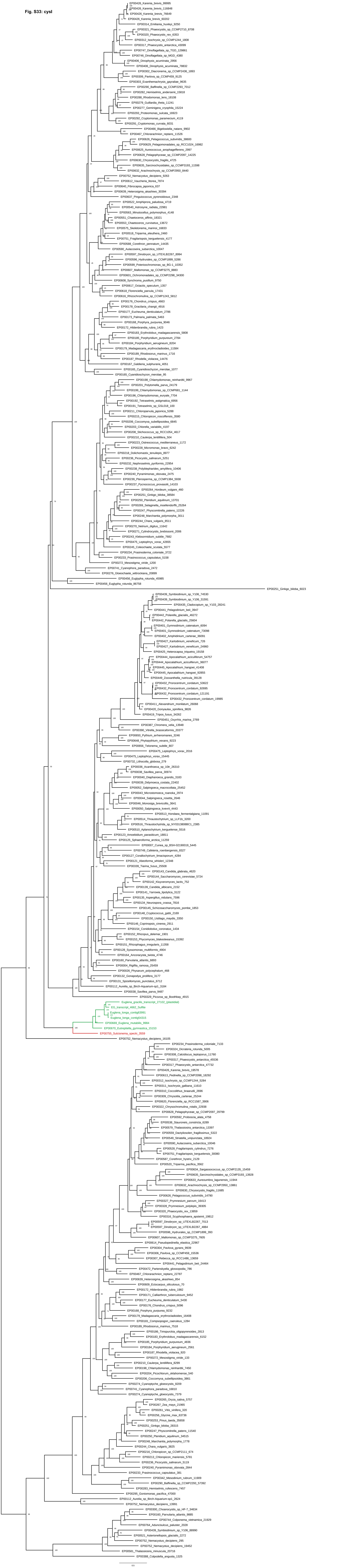

Fig. S33: cysI
